# Systematic review of NTRK 1/2/3 fusion prevalence pan-cancer and across solid tumours

**DOI:** 10.1038/s41598-023-31055-3

**Published:** 2023-03-13

**Authors:** Sophie O’Haire, Fanny Franchini, Yoon-Jung Kang, Julia Steinberg, Karen Canfell, Jayesh Desai, Stephen Fox, Maarten IJzerman

**Affiliations:** 1 Cancer Health Services Research, Centre for Health Policy, Melbourne School of Population and Global Health, Faculty of Medicine, Dentistry and Health Sciences, The University of Melbourne, Melbourne, Australia; 2 Sir Peter MacCallum Department of Oncology, Faculty of Medicine, Dentistry and Health Sciences, The University of Melbourne, Melbourne, Australia; 3 The Daffodil Centre, The University of Sydney, a Joint Venture with Cancer Council New South Wales, Sydney, Australia; 4 Department of Medical Oncology, Peter MacCallum Cancer Centre, Melbourne, Australia; 5 Department of Pathology, Peter MacCallum Cancer Centre, Melbourne, Australia; 6 Erasmus School of Health Policy and Management, Rotterdam, The Netherlands

**Keywords:** Epidemiology, Cancer, Genetic testing

## Abstract

*NTRK* gene fusions are rare somatic mutations found across cancer types with promising targeted therapies emerging. Healthcare systems face significant challenges in integrating these treatments, with uncertainty in prevalence and optimal testing methods to identify eligible patients. We performed a systematic review of *NTRK* fusion prevalence to inform efficient diagnostic screening and scale of therapeutic uptake. We searched Medline, Embase and Cochrane databases on 31/03/2021. Inclusion criteria were studies reporting fusion rates in solid tumours, English language, post-2010 publication and minimum sample size. Critical appraisal was performed using a custom 11-item checklist*.* Rates were collated by cancer type and pooled if additional synthesis criteria were met. 160 studies were included, with estimates for 15 pan-cancer and 429 specific cancer types (63 paediatric). Adult pan-cancer estimates ranged 0.03–0.70%, with higher rates found in RNA-based assays*.* In common cancers, rates were consistently below 0.5%. Rare morphological subtypes, colorectal microsatellite instability, and driver mutation exclusion cancers had higher rates. Only 35.6% of extracted estimates used appropriate methods and sample size to identify *NTRK* fusions. *NTRK* fusion-positive cancers are rare and widely distributed across solid tumours. Small-scale, heterogeneous data confound prevalence prediction. Further large-scale, standardised genomic data are needed to characterise *NTRK* fusion epidemiology.

## Introduction

### Rationale

Genomics enabled precision oncology continues to drive improved and additional treatment options for patients through the development of targeted therapies designed for cancers harbouring specific biomarkers. A significant advancement of this approach is the emergence of ‘Pan Cancer’ or ‘histology independent’ therapies where biomarker positive patients receive a biomarker targeted therapy, irrespective of the physical site of origin of a tumour. Cancer patients whose tumours harbour a gene fusion in the Neurotrophic Tyrosine Receptor Kinase genes 1, 2 or 3 (*NTRK1*, *NTRK* or *NTRK3*) are clinically recommended for treatment in the advanced and refractory setting with targeted therapy in the form of Tyrosine Receptor Kinase (*TRK*) inhibitors^1^. These drugs demonstrated impressive response rates across cancer types in phase 3 clinical trials and both Larotrectinib and Entrectinib received regulatory approvals in recent years with the Food and Drug Administration in the United States, and conditional approval with the European Medicines Agency^2,3^.

Despite growing clinical support for use of *TRK* inhibitors^1^, integration of these drugs into public health systems presents significant challenges for health technology assessment (HTA) agencies who must consider the health economic impact of providing public access to *TRK* inhibitors in a setting of limited resources and growing cancer disease burden^4^. Novel targeted drugs are relatively costly and accurate prevalence estimates are required to inform the relevant cost-effectiveness analyses and health system budget impact of identifying and treating these rare patient groups. Though commonly cited at a prevalence of ‘up to 1% of all cancers’^2^, epidemiological data is extremely limited due to only recent interest in *NTRK* fusions and limited large scale genomic studies using next generation sequencing (NGS) technologies. Specific *NTRK* fusions have been found at a high prevalence in a handful of rare cancer types, but otherwise are widely dispersed and uncommon across other cancer types. It is therefore important to establish the histological types of cancer which are associated with fusion prevalence^5,6^. The rarity of *NTRK* fusions complicates health economic evaluations as uncertainty in the estimated prevalence (precision interval) is greater and minor variations in estimates can substantially impact on projections of drug uptake and cost. Importantly, biomarker testing, often approved as part of access to targeted therapies, requires screening of an extremely large population for a small yield, with impacts to the cost-effectiveness of providing *NTRK* fusion targeted treatment.

Estimation of population prevalence is further confounded by the use of multiple molecular diagnostic tests for identification, with varying diagnostic accuracy, accessibility and cost^5,7,8^. Briefly, *NTRK* fusions occur when the *NTRK* 1, 2 or 3 genes form a chromosomal rearrangement with one of many different genes (fusion partner) and result in the oncogenic expression of a *TRK* fusion protein that drives cancer growth^9,10^. An international expert review recommended DNA or RNA NGS testing for identifying *NTRK* fusions outside of high prevalence cancer types as these broad assays can identify a variety of known and novel fusion partners with high confidence^1^. Alternative molecular tests such as fluorescence in situ hybridization (FISH) and reverse transcription polymerase chain reaction (RT-PCR) are more limited in performance and the range of fusions identifiable, and a Pan-TRK immunohistochemistry antibody is increasingly used as an efficient (cheaper) screen for aberrant TRK protein expression, which would suggest a fusion is present, but has shown poor sensitivity and specificity that varies by cancer type relative to NGS and is not recommended as a sufficient diagnostic method^1,10^. Currently consensus is lacking clinically, and by extension at the regulatory level, for optimal testing and screening algorithms as different approaches to who, how and when to test for these fusions present trade-offs between cost, accessibility and accuracy of various methods^1,11–14^. Given diagnostics with lower sensitivity may underestimate prevalence in a population, this issue is an important consideration for mapping prevalence data.

To date, the most comprehensive estimates for *NTRK* fusion prevalence include those sourced from the largest single genomic testing cohort (Foundation Medicine) with a recent publication interrogating over 200,000 tested patients for *NTRK* fusions^15^. However, there are several potential sources of bias in this cohort such as the selected and likely enriched advanced stage of patients referred for testing. Additionally, the significant majority (~ 95%) of this cohort was tested with a DNA panel, which may underestimate *NTRK* fusion prevalence due to low sensitivity for *NTRK* 2 and 3 fusions^7,10^. Most reviews detailing *NTRK* fusion prevalence cite a convenience selection of one or two data sources, with smaller cancer cohorts of several hundred patients which are not large enough to detect a prevalence < 1% with confidence^16,17^. Only one systematic review of *NTRK* fusion prevalence was identified^18^ which reported prevalence across solid tumours but this search only covered literature up to 2019 and synthesised cancer type cohorts tested for *NTRK* fusions using any method, with a minimum of 20 patients. Little detail was reported to explore the variation and bias in *NTRK* fusion epidemiology, which was noted in discussing the limitations of the current evidence. An exploration of bias and sources of variation in prevalence estimates along with an updated inclusion of more recent data can provide much needed nuanced data to inform health economic evaluations and translation of *NTRK* fusion treatments into routine care.

### Objective

The objective of this review was to extract, synthesise and critically evaluate the prevalence of *NTRK* 1/2/3 fusions in adult and paediatric solid tumour cohorts through a broad review of studies reporting rates Pan-Cancer and across cancer types. Due to the rarity and complexity in identification of these biomarkers, an additional aim was to generate robust point estimates for *NTRK* fusion prevalence in specific cancer type populations through a meta-analysis of rates deemed optimal for pooling. These results hope to inform the significant public health challenge of how to screen a solid-tumour population for *NTRK* fusions and predict rates of patients potentially eligible for treatment with TRK inhibitors.

## Methods

We performed a systematic review adhering to the Preferred Reporting Items for Systematic Reviews and Meta-Analyses (PRISMA) Guidelines^19^ with checklist available in Appendix supplementary table S1 and a protocol was registered on the PROSPERO database on 30/04/2021.

### Search strategy

**Figure 1:**
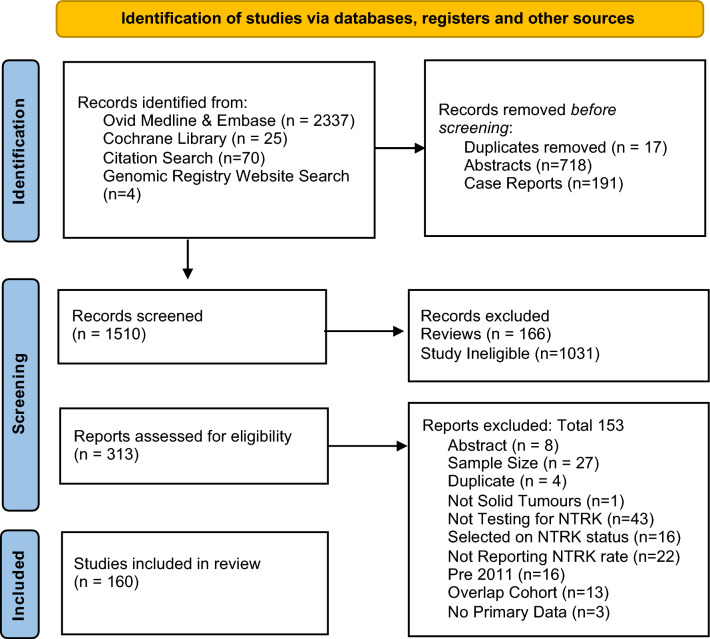
Systematic review workflow.

A combined search of Medline and Embase via Ovid was conducted on 31/03/2021 that involved two components. The principal search was structured to identify articles that mention ‘NTRK’ or ‘TRK’ and terms related to ‘cancer’ and ‘fusions’. To address the increasing number of cohort studies reporting results of genomic testing including targetable fusions, without explicitly referencing ‘NTRK’ as a keyword, we added a supplemental search with keyword ‘genomic’ in conjunction with the most relevant cancer types for this review to identify additional large-scale ‘genomic landscape’ studies. The supplemental search was restricted from 1/1/2020 to 31/3/2021. All identified reviews in the principal search were scanned for relevant citations and this was considered likely to identify relevant ‘genomic landscape’ studies prior to 2020. The Cochrane library was also broadly searched for the keyword ‘NTRK’. Duplications, case reports and abstracts were removed. The review workflow is outlined in Fig. 1 and the detailed search strategy is available in Appendix, supplementary tables S2 and S3.

### Screening and selection criteria

Title and abstract screening was performed by one reviewer (SOH) with independent second review of all records by one of the review team (FF, YJK, JS). Full text review was performed independently by two reviewers (SOH, FF) to ensure studies met selection criteria. In both stages, discrepancies were resolved through discussion. Studies testing for *NTRK* fusions in solid tumours were included if they reported at least one cohort above the minimum sample size for inclusion. Studies from 2010 or earlier were excluded due to outdated test methods and small cohorts limited to thyroid cancer. Studies where *NTRK* fusion status was known before testing or where the cancer type was defined by *NTRK* status were excluded. A detailed table of exclusion and inclusion criteria are available in Appendix, supplementary table S4.

### Data collection

Study characteristics including population demographics, country of origin, study design and assay were extracted for each included study. Fusion prevalence, including relative frequency of *NTRK*1/2/3 were extracted for specific cancer type cohorts reported within an eligible study. Numerators and denominators were extracted to match unique patients, not samples. Only cancer types with cohorts above an absolute minimum sample size (50 common cancer type, 20 rare cancers) were extracted. Pan Cancer estimates were extracted where studies reported rates in cohorts in over ten cancer types. Specific cancer type prevalence rates were extracted as reported, with morphology as detailed as per the article, including those rates within Pan Cancer cohorts if fusion rates were reported at both Pan Cancer and cancer type levels. These cancer type estimates were allocated to broad tumour group categories for collation. Paediatric cohorts were extracted separately wherever possible. Cohorts defined entirely by a molecular subtype of a cancer were extracted as a unique cancer type to explore potential enrichment for *NTRK* fusions. Further information on data extraction and categorisation is available in Appendix section 4. Data extraction and supplementary tables S5, S6 and S7.

### Critical appraisal

A checklist for assessing potential sources of bias and study quality in a cancer profiling context was developed through adapting items from two published checklists identified in a recent systematic review of tools for evaluation of prevalence studies and their ability to assess three domains of external validity, internal validity and statistical/reporting quality^20^. We utilised the Joanna Briggs Critical Appraisal Checklist for Prevalence Studies^21^ and the Risk of Bias in Prevalence Studies Tool^22^. We contextualised items to reflect corresponding practical scenarios of cancer genomic studies as opposed to the more conventional epidemiological survey nature of studies that these tools were designed to evaluate. Several items were synonymous and consolidated into an 11-item appraisal tool for *NTRK* fusion prevalence studies (Section 5. Study appraisal and supplementary table S8, Appendix). With regards to external validity, studies were appraised for their design and recruitment in terms of representing a national cancer type population. Further selection bias issues that would limit generalisability of an estimate such as referral or restrictive eligibility for testing versus a series or registry-based cohort were also assessed. Factors potentially influencing the internal validity or accuracy of *NTRK* fusion rates were assessed including the failure rate of testing, the definition of *NTRK* fusions (only looking for *NTRK1* fusions versus all three *NTRK* genes), and the consistency and accuracy (in terms of existing sensitivity and specificity data) of testing platforms used. Finally, the statistical and reporting quality was considered including the sample size for each rate being sufficiently powered to detect a rare biomarker, clarity in the timeframe of recruitment and the fraction reported positive, and reporting of key clinical demographics (e.g., age, sex, histology) for a tested cohort. Appraisal was performed once per study except for sample size assessment (Item 10) which was performed for each extracted specific cancer rate within a study as this was cancer type dependent. Items had binary ratings (Ideal vs Not Ideal) except for testing methods and sample size which could be classified further in to ‘Ideal’, ‘Okay’ and ‘Poor’ rankings, with thresholds detailed in Appendix, supplementary table S9.

Data extraction and critical appraisal were performed independently by two reviewers (SOH and FF) to confirm consistency and discrepancies resolved through discussion.

### Data synthesis

To capture the broad evidence base and provide robust summary estimates, the synthesis of data was done in two stages: a broad narrative summary and a meta-analysis of high-quality estimates. All included studies with prevalence data extracted were combined into a narrative summary of estimates for the Pan Cancer category and specific cancer cohorts which were collated within tumour groups to explore relative enrichment/variation of fusions in specific cancer types. This narrative summary allowed for summation of key study characteristics while facilitating visual exploration of variation in prevalence estimates within cancer groups and specific types.

In addition to the narrative summary, we aimed to derive robust *NTRK* fusion population prevalence estimates for each unique cancer type using only those rates that met additional synthesis criteria. Specific cancer cohort rates within each tumour group were further collated into ‘unique’ cancer type categories where the name was considered a synonymous type (e.g., Breast Cancer and Breast Carcinoma). Rates for a unique cancer type with multiple estimates were only eligible for meta-analysis if each estimate was from a unique cohort (not overlap), explicitly reported *NTRK* fusion rates, and did not have a ‘poor’ ranking for the methods and sample size ranking as per quality assessment. These two items from the assessment were used for criteria as they provided a quantifiable classification to address two key factors that could lead to inaccuracy (particularly underestimation) of *NTRK* fusion prevalence in a cancer type. We used a generalised linear mixed model with random effects to calculate pooled prevalence if more than one rate met the synthesis criteria per cancer type^23^. Where only one estimate met criteria, this is the prevalence point estimate presented. In both cases these estimates are considered optimal and highlighted in bold in Table 1. For cancer types where no rates were eligible for pooling but fusions were identified, then the study with largest sample size is presented as a less robust estimate (not in bold) to demonstrate the presence of *NTRK* fusions in that cancer type. Cancer types with no rates meeting synthesis criteria and no fusions identified are reported separately in Appendix supplementary table S10, as these studies reported a prevalence rate of 0% but there is less confidence these are reliable null findings. 95% confidence intervals for single study rates were calculated using Clopper Pearson exact method, with calculations and meta-analysis performed using R package ‘*metaprop*’. Pooling was not considered for estimates for Pan Cancer cohorts, paediatric cancers or for cancer types in the ‘Other’ category due to the heterogeneity in cancer type distribution expected across these cohorts. A schema of data synthesis is available in the Appendix supplementary figure S1.

**Table 1:** Summary of studies, rates and synthesis by cancer group and type.

Cancer type	*NTRK* fusion prevalence point estimate	95% Confidence interval	Heterogeneity (I^2^)	Denominator
**Group—Brain/CNS**				
Glioma	**0.51**	**0.20–1.30%**	**I**^**2**^** = 0.90**	**7889**
Glioblastoma multiforme	**1.02**	**0.45–2.28%**	**I**^**2**^** = 0.55**	**1095**
Low grade glioma	**0.94**	**0.31–2.17%**	**–**	**534**
Astrocytoma	**2.22**	**0.90–4.51%**	**–**	**316**
Anaplastic astrocytoma	**0.00**	**0.00–2.74%**	**–**	**133**
Diffuse astrocytoma	**0.00**	**0.00–3.49%**	**–**	**104**
Oligodendroglioma	**0.00**	**0.00–4.12%**	**–**	**88**
Glioma NOS	2.44	0.06–12.86%	–	41
Glioma/neuroepithelial tumour	**0.55**	**0.24–1.07%**	**–**	**1465**
Diffuse leptomeningeal glioneuronal tumour	10.00	2.11–26.53%	–	30
Unknown neurological primary	**0.22**	**0.05–0.63%**	**–**	**1386**
**Group—Breast Cancer**				
Breast cancer	**0.21**	**0.16–0.27%**	**I**^**2**^** = 0.00**	**25,370**
Breast cancer (excludes secretory breast)	**0.08**	**0.02–0.21%**	**–**	**4854**
Secretory carcinoma of the breast	88.64	75.44–96.21%	–	44
**Group—CUP**				
Cancer of unknown primary (CUP)	**0.14**	**0.08–0.23%**	**–**	**10,636**
**Group—Colorectal**				
Appendiceal adenocarcinoma	0.48	0.01–2.65%	–	208
Appendiceal cancer	1.27	0.03–6.85%	–	79
Colon adenocarcinoma	0.23	0.13–0.37%	–	7008
Colon cancer	**0.35**	**0.15–0.68%**	**–**	**2306**
Colorectal adenocarcinoma	0.20	0.09–0.37%	–	4569
Colorectal cancer	**0.22**	**0.18–0.28%**	**I**^**2**^** = 0.02**	**29,578**
MSI-high colorectal carcinoma	3.25	NA*	–	–
MSI-stable colorectal carcinoma	0.18	NA*	–	–
Deficient MMR colorectal cancer	5.60	2.28–11.20%	–	125
RAS/BRAF WT colorectal cancer	0.50	0.01–2.77%	–	199
RAS/BRAF WT, anti-EGFR resistant colorectal cancer	4.26	0.52–14.5%	–	47
**Group—Genitourinary**				
Prostate cancer	**0.14**	**0.08–0.25%**	**I**^**2**^** = 0.00**	**7845**
Prostate adenocarcinoma	**0.00**	**0.00–0.73%**	**–**	**502**
Bladder cancer	**0.21**	**0.10–0.42%**	**I**^**2**^** = 0.00**	**3831**
Bladder urothelial carcinoma	**0.00**	**0.00–0.89%**	**–**	**414**
Renal clear cell carcinoma	**0.00**	**0.00–0.68%**	**–**	**541**
Metanephric adenoma	10.00	1.23–31.70%	–	20
**Group—Gynaecological**				
Cervical cancer	0.33	0.00–1.81%	–	
Uterine	**0.19**	**0.02–0.67%**	**–**	**1080**
Uterine sarcoma	1.15	0.14–4.09%	–	174
Fallopian tube	0.28	0.06–0.81%	–	1078
Ovarian cancer	**0.18**	**0.11–0.28%**	**–**	**11,590**
Ovarian serous cystadenocarcinoma	**0.00**	**0.00–0.86%**	**–**	**428**
**Group—Head and Neck**				
Head and neck (excluding salivary gland cancers)	**0.10**	**0.02–0.28%**	**–**	**3145**
Head and neck squamous cell carcinoma	**0.38**	**0.04–1.38%**	**–**	**522**
Salivary gland cancer (includes secretory carcinoma)	**2.50**	**1.60–3.69%**	**–**	**962**
Secretory carcinoma of the salivary gland	83.33	69.78–92.52%	–	48
**Group—Lung**				
Lung	**0.06**	**0.03–0.10%**	**–**	**21,115**
Non-small cell lung cancer	**0.19**	**0.11–0.33%**	**I**^**2**^** = 0.88**	**60,272**
Non-squamous non-small lung cancer	**0.00**	**0.00–0.40%**	**–**	**909**
Lung adenocarcinoma	**0.09**	**0.03–0.31%**	**I**^**2**^** = 0.32**	**8982**
Lung squamous cell carcinoma	**0.00**	**0.00–0.73%**	**–**	**502**
Large cell neuroendocrine carcinoma	1.45	0.04–7.81%	–	69
Mucinous adenocarcinoma	1.39	0.03–7.50%	–	72
Small cell lung cancer	1.64	0.20–5.80%	–	122
EGFR, ALK, KRAS, ROS1 WT lung adenocarcinoma	3.30	0.69–9.33%	–	91
BRAF, KRAS, EGFR WT lung adenocarcinoma	0.27	0.01–1.69%	–	327
EGFR T790M 2nd Gen TKI resistant non-small cell lung cancer	1.79	0.04–9.55%	–	56
EGFRm post TKI non-small cell lung cancer	**0.10**	**0.03–0.26%**	**–**	**3873**
**Group—Sarcoma**				
Sarcoma	**0.68**	**0.36–1.16%**	**–**	**1915**
Bone sarcoma	**0.16**	**0.00–0.90%**	**–**	**616**
Osteosarcoma	2.65**	0.55–7.56%	–	113
Soft tissue sarcoma	**0.69**	**0.26–1.85%**	**I**^**2**^** = 0.48**	**5080**
Sarcoma NOS (excludes uterine)	**1.17**	**0.54–2.21%**	**–**	**770**
Gastrointestinal stromal tumours	**0.59**	**0.22–1.29%**	**–**	**1009**
Thoracic inflammatory myofibroblastic tumours	9.09	1.92–24.33%	–	33
Leiomyosarcoma	**0.27**	**0.09–0.62%**	**–**	**1865**
**Group—Skin and Melanoma**				
Melanoma	**0.19**	**0.12–0.33%**	**I**^**2**^** = 0.00**	**7203**
Cutaneous melanoma	**0.76**	**0.16–2.20%**	**–**	**395**
Spitzoid neoplasms	**12.50**	**7.32–19.50%**	**–**	**128**
Spitzoid melanoma	4.00	0.10–20.35%	–	25
Mucosal/paramucosal melanoma	0.89	0.02–4.83%	–	113
Pigmented spindle cell nevus of reed	56.52	34.49–76.81%	–	23
**Group—Thyroid**				
Thyroid cancer	**1.68**	**1.08–2.63%**	**I**^**2**^** = 0.67**	**2679**
Papillary thyroid cancer	**2.04**	**1.32–3.13%**	**I**^**2**^** = 0.40**	**1552**
Classic papillary thyroid cancer	0.35	0.04–1.25%	–	575
Classic papillary thyroid cancer (iodine refractory)	5.08	1.06–14.15%	–	59
Tall cell variant papillary thyroid cancer	2.56	0.06–13.48%	–	39
Anaplastic thyroid cancer	1.02	0.12–3.64%	–	196
Hurtle cell carcinoma	2.86	0.07–14.92%	–	35
Poorly differentiated thyroid cancer	2.44	0.06–12.86%	–	41
Malignant thyroid nodule	0.48	0.01–2.64%	–	209
**Group—Upper Gastrointestinal**				
Gastric cancer	**0.14**	**0.05–0.33%**	**–**	**3558**
Small bowel cancer	**0.10**	**0.00–0.54%**	**–**	**1027**
Liver	**0.19**	**0.02–0.68%**	**–**	**1064**
Pancreatic cancer	**0.14**	**0.09–0.22%**	**I**^**2**^** = 0.60**	**13,304**
Pancreatic adenocarcinoma	**0.34**	**0.08–0.78%**	**–**	**1492**
Biliary tract cancer	**0.22**	**0.10–0.49%**	**I**^**2**^** = 0.00**	**2735**
Cholangiocarcinoma	**0.17**	**0.09–0.34%**	**I**^**2**^** = 0.00**	**4692**
Intrahepatic cholangiocarcinoma	3.57	0.09–18.35%	–	28

*ALK* anaplastic lymphoma kinase, *BRAF* v-Raf murine sarcoma viral oncogene homolog B, *CNS* central nervous system, *EGFR* epidermal growth factor receptor, *KRAS* Kirsten rat sarcoma viral oncogene homolog, *mEGFR* metastatic-EGFR, *NOS* not otherwise specified, *RAS* rat sarcoma, *ROS1* c-ros oncogene 1, *TKI* tyrosine kinase inhibitor.

*NA; Not Applicable, rates reported without denominator. **3 fusions identified were novel and confirmed non-functional for expressing TRK protein.

## Results

**Figure 2:**
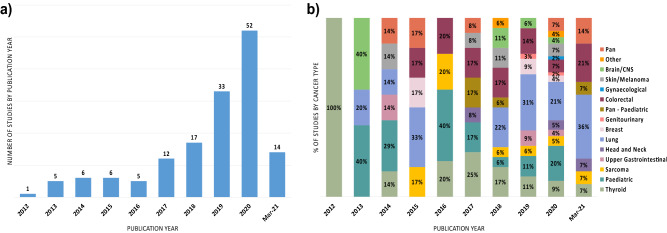
Landscape of included studies. (**a**) Number of studies by publication year; (**b**) relative proportion of studies by cancer type investigated. Mar-2021—number of studies from 01/01/2021 until 31/03/2021.

160 studies were included in the systematic review after full text screening. Between 01/01/2011 and 31/03/2021, no studies met the inclusion criteria for the year 2011, and most studies (62%) were published from 2019 onwards (Fig. 2). 31% of studies (n = 51) were from the United States of America, followed by China and International studies (both 14%). Studies were more commonly retrospective (70%), and approximately one third of studies involved analysis of existing data from genomic data repositories, as opposed to retrospective testing of samples from an identified clinical patient cohort. Most studies (62%) identified *NTRK* fusions through RNA and DNA targeted panels, with DNA panel being the most utilised (n = 45). Overall, 69% of studies performed at least one next generation sequencing assay (n = 114), while the use of histology-based assays remained constant over the period considered and was often used as a primary identification method of fusions, followed by orthogonal validation (n = 26).

*NTRK* Fusion prevalence rates were extracted for 15 Pan Cancer (including 5 Paediatric) cohorts and additionally across 429 specific cancer type cohorts that were allocated to one of fourteen tumour groups. Lung, paediatric, colorectal, and thyroid cancers represented the most analysed cancer types, with respectively 36, 24, 19, and 19 studies, accounting for 61% of all included studies (Fig. 2).

### Narrative summary

**Figure 3:**
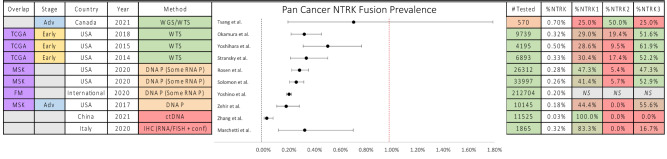
Summary of prevalence of *NTRK* fusions in pan-cancer studies (adult and combined). *TCGA* The Cancer Genome Atlas, *MSK* Memorial Sloan Kettering, *FM* Foundation Medicine, *Adv* Advanced or Metastatic, *USA* United States of America, *DNA/RNA P* DNA/RNA Panel, *+ conf* confirmation of positive expression.

**Figure 4:**
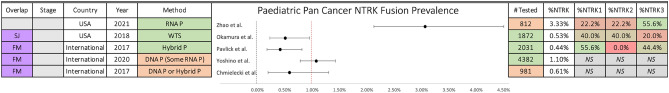
Summary of prevalence of *NTRK* fusions in paediatric pan-cancer studies. *SJ* Saint Jude Children’s Research Hospital, *FM* Foundation Medicine, *USA* United States of America, *DNA/RNA/Hybrid P* DNA/RNA/Hybrid Panel.

The details of the Pan Cancer prevalence estimates are displayed in Figs. 3 and 4. For Adult or mixed age cohorts, these were all below 1%, ranging from 0.03 to 0.70%. Higher rates were seen with more comprehensive testing methods either using an RNA NGS assay or whole genome sequencing (WGS), and lower rates seen for studies using immunohistochemistry (IHC) or circulating tumour DNA (ctDNA) (Fig. 3). Paediatric Pan Cancer cohorts had higher and more variable rates than the adult cohorts, ranging from 0.44 to 3.33%, likely reflecting the greater representation of mid to high *NTRK* fusion prevalence cancer types in paediatric cancers compared to adult (Fig. 4).

Across the 14 tumour groups, the largest number of cancer specific rates (429 in total) were extracted for paediatric cancers (14.69%), lung cancers (13.75%) and thyroid cancers (10.49%). Narrative summary tables for the specific cancer cohort prevalence point estimates are presented for each tumour group in Appendix, supplementary tables S11–S24. High *NTRK* fusion prevalence rates were confirmed in previously identified rare cancers of infantile fibrosarcoma (70.37%), secretory carcinoma of the breast (90.91%) and secretory carcinoma of the salivary gland (range 83.33–89.66%). Additional cancer types with substantial (> 10%) *NTRK* fusion prevalence were largely paediatric cancers; (congenital mesoblastic nephroma (18.75–22.73%), differentiated or papillary thyroid cancer (11.5–26.09%), infantile high-grade glioma (27.59%) and rare skin neoplasms such as spitzoid tumours (12.50–16.43%) and spindle cell nevus of reed (56.52%)). 184 (43.0%) specific cancer type cohorts included had zero *NTRK* fusions identified which may be an indication that these cancer types should not be prioritised for testing but given the rarity of *NTRK* fusions there is also potential that these studies lack sufficient sample size and sensitive methodology to detect fusions at such a low rate.

The relative proportion of *NTRK1*, *NTRK2* and *NTRK3* fusions varied across Pan Cancer cohorts but *NTRK3* fusions were generally the most frequent. This could indicate a higher representation of the canonical (E26 transformation-specific transcription factor 6) *ETV6-NTRK3* fusion in several cancer types but could also reflect testing platforms and referral bias affecting the cancer distribution for each study. Low rates of *NTRK2* fusions identified may be due to the difficulty in identifying fusion breakpoints in *NTRK2* compared to *NTRK1*, particularly using targeted DNA panels^6,10^. 200 of 245 (81.63%) specific cancer cohorts that reported fusions specified the *NTRK* gene involved. Of these, *NTRK1* and *NTRK3* fusions were commonly reported (137, 68.50%) and (128, 64.00%) but *NTRK2* fusions were only identified in (46, 23.00%) cohorts. Ten cohorts listed *NTRK2* fusions exclusively which were either brain/CNS tumours or lung adenocarcinoma.

### Meta-analysis for population prevalence estimates

To arrive at population-point estimates for *NTRK* fusion prevalence, (332) specific cancer cohort rates were consolidated into 135 unique cancer types and assessed for suitability for meta-analysis. Rates for 85 types are presented in Table 1, with 50 cancer types listed separately in Appendix supplementary table S10 as none of these were eligible for meta-analysis and no fusions were identified. Studies included in the meta-analysis are highlighted in blue in supplementary tables S11–S24 of the Appendix. A total of 48 (35.56%) cancer types had rates considered eligible for pooling and just 15 (11.11%) had multiple cohorts eligible. Point estimates for major cancer types such as Colorectal Cancer, Non-Small Cell Lung Cancer, Melanoma, Breast, Prostate and Pancreatic Cancer were very low, ranging between 0.10–0.25%, confirming the rarity of the fusions in broad unselected cancer populations. Higher rates were seen in Thyroid and Sarcomas, but still rare at around 1%. Enrichment was seen in molecular subtypes such as driver WT (wild type) lung and MSI-H/dMMR (microsatellite instability-high/deficient in mismatch repair) colorectal cancers, and in both cancers post-treatment cohorts revealed *NTRK* fusions as resistant mutations at relatively higher prevalence. For all cancer types with multiple cohorts eligible for pooling, non-zero prevalence of *NTRK* fusions were found. This suggests, despite their rarity, the evidence supports the wide range of cancer types that potentially harbour *NTRK* fusions at very low frequencies. A comparison of the point estimates in this study, a previous meta-analysis and the Foundation Medicine cohort are presented for common cancer types in the Appendix supplementary table S25 (and demonstrate consistency for these broad groups).

### Critical appraisal

Of the 444 rates extracted (15 Pan and 429 specific cancer), over half (261, 58.78%) had sample sizes deemed insufficient to identify *NTRK* fusions at the expected prevalence with 95% confidence as per item 11 of the critical appraisal tool (Appendix, supplementary table S8), with only (83, 18.69%) having ideal sample size. The methods used to detect *NTRK* fusions were assessed at the study level, with over 70% studies using methods considered ideal (RNA based NGS assays or WGS, 39.62%) or okay (DNA NGS assays, 32.08%), but substantial variation in specific testing methods was evident within each of these categories. With regards to generalising prevalence rates for population estimates, a minority of studies were considered as nationally representative cohorts (10.37%), largely due to studies being single site or limited in geographic coverage. Additionally, 54.88% studies had risk of external validity bias through systematically selected cohorts (as opposed to consecutive series) due to referral-based recruitment and restricted study eligibility criteria such as patient prognosis or minimal sample requirements for testing. Further to the assessment of testing methods used, the internal validity of each study’s rates was assessed for underestimation of *NTRK* fusion prevalence with 20.12% of studies having greater than a 5% failure rate in cohort testing and 28.30% of studies explicitly looking for *NTRK* fusions in less than the three *NTRK* genes or assessing limited fusion partners. Notably, only 60.37% of studies reported the demographics of the tested cohort in terms of age, sex, stage, morphology, and ethnicity, illustrating a reporting gap for 40% of studies with unclear representativeness of basic cohort demographics. The least reported demographic item was ethnicity, which was not included in 59.15% of studies. Results of the customised study appraisal tool are available in supplementary table S26 in the Appendix.

## Discussion

This review of the current available evidence for *NTRK* fusion prevalence supports the notion that they are extremely rare and widely distributed across solid tumours. The Pan Cancer estimates for adult cohorts all sit comfortably below the commonly cited ‘1%’ prevalence approximation, with prevalence for paediatric cohorts also rare but slightly higher. Variation in these rates could be due to the different sensitivities of testing methods used, such as the low rates seen for cohorts using IHC or ctDNA relative to more comprehensive and reliable NGS on tissue samples. However, variation is also likely due to the relative distributions of cancer types included in these studies, and potential enrichment for high prevalence *NTRK* fusion cancers such as infantile fibrosarcoma and gliomas in some paediatric cohorts.

Our meta-analysis results indicate that the prevalence of *NTRK* fusions in common cancer types is also very low, with many close to 0.20% and upper bound confidence intervals below 0.50%. These results have utilised more recent data sources to provide a comprehensive evidence map of prevalence data and validate common cancer type rates described in Forsythe et al.^18^. There is also consistency with our rates and the predominant data source of Foundation Core, which through substantially larger cohorts, has considerable influence on our pooled prevalence.

With confidence that these rates are representative of the current literature through broad review, *NTRK* fusion prevalence estimates and the associated confidence intervals can be used in a public health context to predict targeted therapy uptake and inform health technology assessments for specific cancer types with the likely impact of drug uptake costs across a cancer population being minimal. The real challenge for integration of targeted therapies for rare biomarkers is the efficiency of testing and identifying positive patients. Our results indicate a thousand patients with common cancer types would need testing to identify two or three patients with *NTRK* fusions. Therefore, this supports the rationale of broad NGS testing (ideally a combined DNA/RNA platform) for advanced cancer patients with greater capability for detecting the broad range of *NTRK* fusion types, but also a spectrum of other targetable and potentially rare fusions and mutations. Additionally, highlighting subpopulations enriched for *NTRK* fusions allows for a more cost-effective approach to screening and the detail in this review provides quantitative evidence to support this approach which has been suggested in literature^11,24^. Prioritised testing for rare histology cancers with higher prevalence as listed in the results offers efficiency, but our evidence also indicates enrichment in cancer types based on molecular status such as lung cancers negative for other drivers, microsatellite instability high colorectal cancers and in some post-therapeutic cohorts with *NTRK* fusions emerging as potential biomarkers of drug resistance. However, large scale data indicate this is evidence of enrichment not exclusivity^25,26^, so although screening patients using sequential biomarker testing may be more cost effective in the specific context of rare *NTRK* fusions this may still miss positive patients, requiring more time and tumour tissue to arrive at a result compared to upfront comprehensive NGS testing which would be the preferred option from a patient perspective, ideally on fresh frozen tissue. Where this technology is not available routinely, *NTRK* FISH or pan-TRK IHC can be broad practical diagnostic options provided sufficient archival tissue is available and could be considered for not only high prevalence cancers or enriched molecular sub-types (such as MSI-H colorectal cancer), but potentially any solid cancer in clinical need of treatment. Given the diagnostic performance of these tests vary by cancer and fusion type, awareness of the evolving literature on validation of *NTRK* fusion diagnostics is critical for pathologists using these methodologies^7,8^ and could further inform optimal population screening algorithms along with accurate prevalence estimates.

More broadly, the results of this review demonstrate the challenge in consolidating evidence for biomarker epidemiology in the context of a rare biomarker. Thorough assessment using a structured tool allowed for comparison and detailed scoping of evidence quality, and this methodology could be adapted for other biomarkers in cancer genomic studies to derive robust prevalence estimates. Despite a large number of included studies, almost 60% of rates extracted had insufficient sample size. This mandates larger scale profiling studies and consolidation of genomic data, but standardisation of factors such as disease ontology, and transparency in methods and demographic reporting is critical to data synthesis being meaningful. Rare targets such as *NTRK*, *NRG1* (neuregulin 1) and *RET* (rearranged during transfection) fusions are becoming increasingly relevant for clinical management Pan-Cancer and efforts to unify biomarker epidemiology data is critical from a public health perspective to understand the potential impact of novel targeted therapies at a population level, and ensure these patients are identified efficiently.

This review aimed to minimise reporting bias through adding a supplemental genomic study search rather than just studies focused on *NTRK* fusion identification but there are certainly NGS profiling studies that would not be captured in this search strategy. Large institutional precision oncology programs may not publish results regularly and an inclusion of international genomic registry data would likely add depth to the evidence base for *NTRK* fusion prevalence. Our decision to synthesise studies using DNA NGS panels rather than restricting to preferred RNA based or WGS methods only means pooled estimates may underestimate the true prevalence, but far fewer studies met this criterion and estimates at least reflect a prevalence that may be identified with population level DNA panel testing which are currently much more accessible at scale through commercial platforms.

## Conclusion

This review provides comprehensive evidence on the prevalence of *NTRK* fusions in solid tumours available in the literature to date. Although rare, the range of cancer types with fusions is extensive, and detailed and accurate epidemiological data is critical to health service planning that supports efficient identification and treatment of patients with these rare, yet targetable Pan Cancer biomarkers.

## Supplementary Information

Supplementary Information.

## Data Availability

Data outlined in this review is available through the article and within the appendix.

## Abbreviation

NTRK: Neurotrophic tyrosine receptor kinase
