# Supplementary material for: Systematic review of NTRK 1/2/3 fusion prevalence pan-cancer and across solid tumours
Source: Sci Rep. 2023 Mar 13;13:4116. doi: 10.1038/s41598-023-31055-3 (PMC10011574; doi:10.1038/s41598-023-31055-3)
Supplement: Supplementary file 1 — Supplementary Information. [file 41598_2023_31055_MOESM1_ESM.docx]

**Systematic Review of NTRK 1/2/3 Fusion Prevalence Pan-Cancer and across Solid Tumours**

**APPENDIX: Detailed methods and results**

**RUNNING TITLE: Prevalence of NTRK 1/2/3 Fusion across Solid Tumours**

**CORRESPONDING AUTHOR:** Sophie O’Haire

Cancer Health Services Research, University of Melbourne

Email: sophie.ohaire@petermac.org

Table of Contents

List of supplementary tables 3

List of supplementary figures 3

1. Preferred Reporting Items for Systematic reviews and Meta-Analyses (PRISMA) Checklist 4

2. Database Search Terms for Ovid Platform (Combined Medline & Embase) 7

3. Eligibility Criteria 8

4. Data extraction 8

A. Country 8

B. Cancer stage categories 8

C. Tumour group categories 9

D. Testing categories 10

E. NTRK fusion rates and types 11

5. Study appraisal 11

A. Background 11

B. Purpose 11

C. Methods 11

6. Development of sample size matrix for approval of sample sizes 14

7. Algorithm for synthesis explained 15

8. Unique cancer types with no estimates eligible for pooling and no fusions identified 16

9. Prevalence Rates for All Specific Cancer Cohorts Extracted 18

10. Comparison with other key studies 43

11. Bias assessment 44

12. References 49

# List of supplementary tables

Supplementary Table S1. PRISMA Checklist 4

Supplementary Table S2. NTRK Focussed Search (Medline & Embase) 7

Supplementary Table S3. Broader Genomic Landscape Study Search (Medline & Embase) 7

Supplementary Table S4. Inclusion and exclusion criteria 8

Supplementary Table S5. Stage categories 9

Supplementary Table S6. Tumour groupings 9

Supplementary Table S7. Testing categories and assay types 10

Supplementary Table S8. Customised study appraisal tool for NTRK Fusion prevalence studies 12

Supplementary Table S9. Sample size calculations for study appraisal across expected prevalence categories 14

Supplementary Table S10. Unique cancer types without estimates eligible for pooling and no fusions identified 16

Supplementary Table S11. NTRK Fusion Prevalence for Brain/Central Nervous System (CNS) Tumours 18

Supplementary Table S12. NTRK Fusion Prevalence for Breast Cancers 19

Supplementary Table S13. NTRK Fusion Prevalence for Cancer of Unknown Primary 20

Supplementary Table S14. NTRK Fusion Prevalence for Colorectal Cancers 21

Supplementary Table S15. NTRK Fusion Prevalence for Genitourinary Cancers 23

Supplementary Table S16. NTRK Fusion Prevalence for Gynaecological Cancers 24

Supplementary Table S17. NTRK Fusion Prevalence for Head & Neck Cancers 25

Supplementary Table S18. NTRK Fusion Prevalence for Lung Cancers 27

Supplementary Table S19. NTRK Fusion Prevalence for Melanoma & Skin Cancers 30

Supplementary Table S20. NTRK Fusion Prevalence for Paediatric Cancers 32

Supplementary Table S21. NTRK Fusion Prevalence for Sarcomas 35

Supplementary Table S22. NTRK Fusion Prevalence for Thyroid Cancers 36

Supplementary Table S23. NTRK Fusion Prevalence for Upper Gastrointestinal Cancers 39

Supplementary Table S24. NTRK Fusion Prevalence for Other Cancers 41

Supplementary Table S25. Comparison of estimates between our study and Forsythe et al. and Foundation Medicine. 43

Supplementary Table S26. Critical appraisal of included studies. 44

# List of supplementary figures

Supplementary Figure S1. Schema of Data Synthesis 15

# Preferred Reporting Items for Systematic reviews and Meta-Analyses (PRISMA) Checklist

Supplementary Table S1. PRISMA Checklist

| **Section and Topic** | **Item #** | **PRIMSMA Checklist item** | **Location where item is reported** |
| --- | --- | --- | --- |
| **TITLE** | | |  |
| Title | 1 | Identify the report as a systematic review. | Page 1 |
| **ABSTRACT** | | |  |
| Abstract | 2 | See the PRISMA 2020 for Abstracts checklist. | Page 2 |
| **INTRODUCTION** | | |  |
| Rationale | 3 | Describe the rationale for the review in the context of existing knowledge. | Pages 3-5 |
| Objectives | 4 | Provide an explicit statement of the objective(s) or question(s) the review addresses. | Page 6 |
| **METHODS** | | |  |
| Eligibility criteria | 5 | Specify the inclusion and exclusion criteria for the review and how studies were grouped for the syntheses. | Pages 6-7; Appendix table S4 |
| Information sources | 6 | Specify all databases, registers, websites, organisations, reference lists and other sources searched or consulted to identify studies. Specify the date when each source was last searched or consulted. | Pages 6-7 |
| Search strategy | 7 | Present the full search strategies for all databases, registers and websites, including any filters and limits used. | Page 6; Appendix tables S2-S3 |
| Selection process | 8 | Specify the methods used to decide whether a study met the inclusion criteria of the review, including how many reviewers screened each record and each report retrieved, whether they worked independently, and if applicable, details of automation tools used in the process. | Page 7 |
| Data collection process | 9 | Specify the methods used to collect data from reports, including how many reviewers collected data from each report, whether they worked independently, any processes for obtaining or confirming data from study investigators, and if applicable, details of automation tools used in the process. | Pages 7-8 |
| Data items | 10a | List and define all outcomes for which data were sought. Specify whether all results that were compatible with each outcome domain in each study were sought (e.g. for all measures, time points, analyses), and if not, the methods used to decide which results to collect. | Pages 8-9 |
|  | 10b | List and define all other variables for which data were sought (e.g. participant and intervention characteristics, funding sources). Describe any assumptions made about any missing or unclear information. | Appendix section 4. |
| Study risk of bias assessment | 11 | Specify the methods used to assess risk of bias in the included studies, including details of the tool(s) used, how many reviewers assessed each study and whether they worked independently, and if applicable, details of automation tools used in the process. | Pages 8-9 |
| Effect measures | 12 | Specify for each outcome the effect measure(s) (e.g. risk ratio, mean difference) used in the synthesis or presentation of results. | Page 7 |
| Synthesis methods | 13a | Describe the processes used to decide which studies were eligible for each synthesis (e.g. tabulating the study intervention characteristics and comparing against the planned groups for each synthesis (item #5)). | Pages 9-10 |
|  | 13b | Describe any methods required to prepare the data for presentation or synthesis, such as handling of missing summary statistics, or data conversions. | Page 10 |
|  | 13c | Describe any methods used to tabulate or visually display results of individual studies and syntheses. | Page 10 |
|  | 13d | Describe any methods used to synthesize results and provide a rationale for the choice(s). If meta-analysis was performed, describe the model(s), method(s) to identify the presence and extent of statistical heterogeneity, and software package(s) used. | Page 10 |
|  | 13e | Describe any methods used to explore possible causes of heterogeneity among study results (e.g. subgroup analysis, meta-regression). | Page 10 |
|  | 13f | Describe any sensitivity analyses conducted to assess robustness of the synthesized results. | Page 10 |
| Reporting bias assessment | 14 | Describe any methods used to assess risk of bias due to missing results in a synthesis (arising from reporting biases). | Pages 8-9; Appendix 3. and Table S6 |
| Certainty assessment | 15 | Describe any methods used to assess certainty (or confidence) in the body of evidence for an outcome. | Page 10 |
| **RESULTS** | | |  |
| Study selection | 16a | Describe the results of the search and selection process, from the number of records identified in the search to the number of studies included in the review, ideally using a flow diagram. | Page 11 |
|  | 16b | Cite studies that might appear to meet the inclusion criteria, but which were excluded, and explain why they were excluded. | Page 11 |
| Study characteristics | 17 | Cite each included study and present its characteristics. | Page 11 |
| Risk of bias in studies | 18 | Present assessments of risk of bias for each included study. | Pages 13-14 |
| Results of individual studies | 19 | For all outcomes, present, for each study: (a) summary statistics for each group (where appropriate) and (b) an effect estimate and its precision (e.g. confidence/credible interval), ideally using structured tables or plots. | Pages 11-13 |
| Results of syntheses | 20a | For each synthesis, briefly summarise the characteristics and risk of bias among contributing studies. | Pages 11-12 |
|  | 20b | Present results of all statistical syntheses conducted. If meta-analysis was done, present for each the summary estimate and its precision (e.g. confidence/credible interval) and measures of statistical heterogeneity. If comparing groups, describe the direction of the effect. | Pages 12-13 |
|  | 20c | Present results of all investigations of possible causes of heterogeneity among study results. | Page 14 |
|  | 20d | Present results of all sensitivity analyses conducted to assess the robustness of the synthesized results. | Page 13 |
| Reporting biases | 21 | Present assessments of risk of bias due to missing results (arising from reporting biases) for each synthesis assessed. | Pages 13-14 |
| Certainty of evidence | 22 | Present assessments of certainty (or confidence) in the body of evidence for each outcome assessed. | Pages 13-14 |
| **DISCUSSION** | | |  |
| Discussion | 23a | Provide a general interpretation of the results in the context of other evidence. | Pages 14-15 |
|  | 23b | Discuss any limitations of the evidence included in the review. | Page 16 |
|  | 23c | Discuss any limitations of the review processes used. | Pages 16-17 |
|  | 23d | Discuss implications of the results for practice, policy, and future research. | Page 17 |
| **OTHER INFORMATION** | | |  |
| Registration and protocol | 24a | Provide registration information for the review, including register name and registration number, or state that the review was not registered. | Page 6 |
|  | 24b | Indicate where the review protocol can be accessed, or state that a protocol was not prepared. | Page 6 |
|  | 24c | Describe and explain any amendments to information provided at registration or in the protocol. | Page 6 |
| Support | 25 | Describe sources of financial or non-financial support for the review, and the role of the funders or sponsors in the review. | Page 20 |
| Competing interests | 26 | Declare any competing interests of review authors. | Page 21 |
| Availability of data, code and other materials | 27 | Report which of the following are publicly available and where they can be found: template data collection forms; data extracted from included studies; data used for all analyses; analytic code; any other materials used in the review. | Page 17  Appendix |

From Page MJ, McKenzie JE, Bossuyt PM, Boutron I, Hoffmann TC, Mulrow CD, et al. The PRISMA 2020 statement: an updated guideline for reporting systematic reviews. BMJ 2021;372:n71. doi: 10.1136/bmj.n71

# Database Search Terms for Ovid Platform (Combined Medline & Embase)

Cochrane Library was searched using the single term “NTRK”.

Supplementary Table S2. NTRK Focussed Search (Medline & Embase)

| **No** | **Search terms** |
| --- | --- |
| 1 | ((Ntrk* or TRK8) adj3 (fusion* or rearrange*)).mp. |
| 2 | ((neurotrophic tyrosine receptor kinase or neurotrophic tyrosine kinase) adj3 (fusion* or rearrange*)).mp. |
| 3 | Exp gene fusion/ |
| 4 | Exp oncogene proteins, fusion/ |
| 5 | (NTRK* or TRK*).mp. |
| 6 | 3 and 5 |
| 7 | 4 and 5 |
| 8 | 1 or 2 or 6 or 7 |
| 9 | Exp neoplasms/ |
| 10 | (neoplasm* or tumo?r* or carcinoma* or cancer* or malignan*).mp. |
| 11 | 9 or 10 |
| 12 | 8 and 11 |
| 13 | Exp animal/ |
| 14 | Exp human/ |
| 15 | 13 not (13 and 14) |
| 16 | 12 not 15 |
| 17 | Limit 16 to English language |
| 18 | Remove duplicates from 17 |

Supplementary Table S3. Broader Genomic Landscape Study Search (Medline & Embase)

| **No** | **Search terms** |
| --- | --- |
| 1 | Genomic*.mp. |
| 2 | (lung adenocarcinoma or lung cancer*).mp. |
| 3 | (prostate adenocarcinoma or prostate cancer*).mp. |
| 4 | (colorectal adenocarcinoma or colorectal cancer*).mp. |
| 5 | Breast cancer* |
| 6 | Melanoma.mp. |
| 7 | Sarcoma*.mp. |
| 8 | Thyroid cancer*.mp. |
| 9 | P?ediatric cancer*.mp. |
| 10 | CNS cancer*.mp. |
| 11 | Brain cancer*.mp. |
| 12 | (pan-cancer or pan cancer).mp. |
| 13 | (profiling or target* or fusion* or rearrangement*).mp. |
| 14 | 2 or 3 or 4 or 6 or 7 or 8 or 9 or 10 or 11 |
| 15 | 13 and (5 or 12) |
| 16 | 1 and (14 or 15) |
| 17 | Limit 16 to yr=”2020-2021” |
| 18 | Limit 17 to “review articles” |
| 19 | 17 not 18 |
| 20 | Limit 19 to English language |
| 21 | Limit 20 to humans |

# Eligibility Criteria

Supplementary Table S4. Inclusion and exclusion criteria

|  | **Inclusion** | **Exclusion** |
| --- | --- | --- |
| Condition | - Rate/numerator of NTRK fusions identified in a cohort and tested denominator in terms of patients (not samples). - Test Platforms (TRK IHC with positive expression validation, or RT-PCR, FISH, or any NGS assay capable of detecting NTRK fusions (including ctDNA)) | - Pan TRK IHC or TRKA/B/C IHC used to identify fusion cases without confirmation of positive cases via alternative method * - Fusions not reported/obtainable as a per patient rate |
| Context | - Cohort Studies, Pathology archives, Commercial testing cohort. RCTs - Overlap Cohort - included if these provide differing levels of detail or larger only is included. - Recent non explicit zero studies | - Studies using samples selected based on known driver mutation/fusion status - Studies reporting NTRK status in cancer types that are defined by the presence/absence of fusion - Abstracts/Case Reports/Non-English - 2010 or older ** |
| Population | - Testing Solid Tumours - Sample Size of 50 or more. Rare cancer types with more than 20 pts will be considered* - Pan Cancer requires >10 cancer types and SS >500 | - Haematological Malignancies - Non-human studies (animal/cell lines) |

*Using CoCoPop question format in place of PICO for Prevalence Review (Munn et al., BMC MRM, 2018)*

* Rare cancer types were defined using the RARECARE reference list defined as incidence of <6 per 100,000 in the European population - (http://rarecarenet.istitutotumori.mi.it/)

** The decision to exclude studies pre-2011 was made after publication of the protocol on PROSPERO as there were fewer than 10 deemed eligible studies and all were predominantly in Thyroid cancers, using outdated testing methods.

# Data extraction

## Country

We extracted the country from which the patients were recruited, or from which the samples were sourced. When studies included separate cohorts, they were extracted separately (e.g., adult versus paediatric), including the country. When a cohort was constituted of patients recruited in multiple countries or if the study was international, the country for the study was categorised as international. Studies involving commercial laboratories for testing and for which no information was provided regarding the country were categorised as not specified (NS).

## Cancer stage categories

Not all studies provided details on patients’ cancer stage, and the categorisation was not always consistent between studies. Cancer stage at recruitment was extracted for prospective studies, while the cancer stage at testing was extracted for retrospective studies. We considered data on NTKR prevalence for early-stage cancers and advanced-stage cancers where available. When both early and advanced stages were reported in a study, the assigned stage category was mixed.

Supplementary Table S5. Stage categories

| **Cancer stage category** | **Different study-specific definitions** |
| --- | --- |
| **Early stage** | - Stage I, II, III (any of these and their combinations) - Early stage cancer - For studies of brain cancer: Grade 1 or 2 [low grade] |
| **Advanced stage** | - Stage III/IV - Stage IV - Locally advanced cancer - Advanced cancer - Metastatic cancer - Relapsed and/or refractory cancer - For studies of brain cancer: Grade 3 or 4 [high grade] |

## Tumour group categories

All specific cancer type estimates were extracted as detailed as per source article in terms of topography and morphology. Estimates were then allocated a broad tumour group to assist with narrative summary and assessing synonymous cancer type cohorts for meta-analysis. Groupings were based on primary site largely, in line with ICD-10 coding, except for Sarcoma and Paediatric estimates. Some synonyms were considered the same unique type (e.g., small bowel and small intestine, breast cancer and breast carcinoma), and this was based on Oncotree [1] and expert clinical input. Once allocated a grouping, estimates of similar cancer types were assessed for grouping at the more detailed level, where they were considered to represent the same unique cancer type within the broader grouping. Molecular subtypes were considered separately as independent types.

For example, cancer specific estimates extracted as small bowel cancer, pancreatic cancer and small intestine were all allocated to the Upper Gastrointestinal Grouping, and Small Intestine was considered an equivalent cancer type to Small Bowel Cancer.

Supplementary Table S6. Tumour groupings

| **Tumour group** | **Cancer types and selected cancer sub-types included in meta-analyses** |
| --- | --- |
| **Brain / Central nervous system (CNS) cancers** | Includes all brain tumours and central nervous system malignancies |
| **Breast cancers** | Includes any tumours arising from the breast |
| **Cancers of Unknown Primary (CUP)** | Includes all cancers of unknown primary |
| **Colorectal cancers** | Includes any cancers across the lower intestine   - Anal cancer - Appendiceal cancer - Colon cancer - Colorectal cancer - Rectal cancer |
| **Genitourinary cancers** | Includes all genitourinary cancers   - Bladder/urothelial cancer - Kidney cancer - Penile cancer - Prostate cancer - Testicular cancer |
| **Gynaecological cancers** | - Cervical cancer - Endometrial cancer - Ovarian cancer - Uterine cancer - Vulvar cancer |
| **Head and neck cancers** | - Head and neck cancers - Salivary gland carcinoma |
| **Lung cancers** | Includes all lung cancers   - Mesothelioma - Small cell lung cancer - Non-cell lung cancer |
| **Melanoma and skin cancers** | Includes any skin cancers   - Melanoma - Non-melanoma |
| **Paediatric cancers** | Includes any cohort completely limited to under 18 years of age |
| **Sarcomas** | - Bone sarcoma - Soft tissue sarcoma |
| **Thyroid cancers** | Includes all thyroid cancers |
| **Upper gastrointestinal cancers (GI)** | - Esophageal cancer - Gastric cancer - Liver cancer - Pancreatic cancer - Small bower cancer |
| **Other cancers** | - Endocrine cancer - Mesothelioma - Neuroendocrine cancer - Thymic cancer |

## Testing categories

The assay used for testing was extracted in detail and then condensed into categories and assay types (Table S7). Where studies utilised a unique assay, they were reported as stated (e.g., study 57 aka 27 used Mass Spectrometry and with multiplex PCR) and grouped in “other” category. When Next Generation Sequencing (NGS) was used, we extracted whether the testing was performed in house – at the recruitment site, such as a hospital or an academic centre – or at a commercial testing facility. Where studies have used multiple NSG assays, “Multiple NSG” was reported. Studies using histology-based, RT-PCR, SNP array, and assays under the ‘Other’ category were not classified as NGS-based.

Supplementary Table S7. Testing categories and assay types

| **Category** | **Assay types** |
| --- | --- |
| **Histology** | - Immunohistochemistry (IHC) - Fluorescence In Situ Hybridisation (FISH) |
| **Targeted panels and next generation sequencing** | - RT-PCR - DNA panel - RNA panel - Hybrid panel - SNP array - Circulating tumour DNA (ctDNA) - Whole transcriptome sequencing (WTS) - Whole exome sequencing (WES) - Whole genome sequencing (WGS) |
| **Other** | - Mass spectrometry and multiplex PCR - Genome wide DNA methylation and copy-number variation |

## NTRK fusion rates and types

For each study, the number of positive cases detected (numerator) and the total number of successfully tested patients (denominator) were extracted. In addition, we excluded haematological tumours where possible from the denominator.

We extracted the type of NTRK fusions (1/2/3) from the studies where possible, and used registry portals (MSK, cBioPortal) or supplementary figures and tables when not readily available from the main manuscript.

# Study appraisal

Assessing bias and quality in observational somatic molecular studies for estimating biomarker prevalence.

## Background

- Bias assessment in Systematic Reviews are established and focus mainly on intervention studies and the internal validity of results [2].
- Limited checklists exist for assessing prevalence studies, but these assume study design is for epidemiological purposes, which is rarely the case for somatic profiling. A recent systematic review by Migliacava et al, 2020, consolidated these tools and recommended use of Joanna Briggs Critical Appraisal Checklist, and noted the considerable overlap in items across tools addressing key domains (external validity, internal validity, and statistical & reporting quality).
- Context for consideration is Health Technology Assessment (HTA) of biomarker testing that requires (national) scale estimates based on somatic profiling cohort studies. Understanding how estimates may deviate from the true prevalence in the population can help inform sensitivity analyses and translational decisions.

## Purpose

- Explore the detail in pros and cons of studies in an epidemiological framework to understand potential bias/quality issues in prevalence estimates of rare biomarkers where large scale/national epidemiological surveys are unlikely.
- The tool will assess domains of potential bias in prevalence estimates (external validity, internal validity, and statistical & reporting quality). The impact of some items may be relatively minor, and some will not be quantifiable, however the tool will provide two functions for the review:
  1. to highlight the complexity of deriving accurate estimates and recognise superior studies,
  2. to use some items in the tool to apply criteria for meta-analysis.

## Methods

- We created an adapted checklist using two existing published studies and contextualised the items to somatic profiling processes and applied criteria for some items specific to NTRK fusions (identification methods and sample size).
- Using adaptation of the ‘JBI critical appraisal checklist for studies reporting prevalence data’ [3] and a ‘risk of bias tool for prevalence studies’ [4] as options recommended by recent systematic review [5].

Supplementary Table S8. Customised study appraisal tool for NTRK Fusion prevalence studies

| **Source ^a^** | **Risk Of Bias Item** | ***Context/considerations*** | **Response** | **Notes** |
| --- | --- | --- | --- | --- |
| *Source (Hoy Item 1)* | 1. Was the target population a nationally representative sample?  *(considering stage/age/ cancer type distributions)* | YES   - Target population was a multi-site nationally representative population   NO   - Limited geographic coverage (single site, region) - Clinical subset of this population (e.g., specific age limits, treatment history, ethnicity, or exposure) - Unclear on geographical coverage - Unclear on clinical restriction | Yes - Low Risk | *Target Population is the group to which results will be generalised, akin to ‘study eligibility’.*  *Defined by study, may be national population or patients of a specific hospital.* |
|  |  |  | No - Potential Bias |  |
| *Source (JBI Item 1 & Hoy Item 2)* | 2. Was the sample frame appropriate to address the target population? | YES   - There was a list of all eligible target population participants to recruit for this study   NO   - Sampling frame definition not clear/stated - Referral based recruitment, factors outside eligibility may impact being included - If retrospective testing cohort of NGS data, sample requirements may be a source of bias as samples that failed testing not included - Explicit that not all eligible patients identified | Yes - Low Risk | *Sample Frame is the list of the target population identified that can be included in the study.* |
|  |  |  | No - Potential Bias |  |
| *Source (JBI Item 2 & Hoy Item 3)* | 3. Were random or exhaustive recruitment undertaken? | YES   - For testing studies, all identified cases (or a randomly selected subset) were included - For data analysis studies, all cases with data (or a randomly selected subset) included   NO   - Enrolment of identified pts/cases methods not clear - Enrolment of identified pts/cases restricted based on practical constraint (resourcing, strict pre-test sample requirements) | Yes - Low Risk | *Random selection is unlikely in this context, not practical for rare biomarker and not standard for genomic testing studies* |
|  |  |  | No - Potential Bias |  |
| *Source (JBI Item 9 or 5 & Hoy Item 4)* | 4. Was the response (result) rate adequate? | YES   - 99% or more of patients tested in the study obtain a result - For data analysis studies this will always be low risk   NO   - Test failure rate minimal (<5%) - Test failure rate >5% - Test failure rate not reported? | Yes - Low Risk | *High success threshold is based on expected prevalence for most cancers of <1%* |
|  |  |  | No - Potential Bias |  |
| *Source (Hoy Item 5)* | 5. Were the samples tested reflective of the stage of the patient cohort? | YES   - Sample represents cohort through (e.g., biopsy at recruitment, surgical resections of early stage cohort)   NO   - The stage of sample used was not reflective of the - The stage of sample used was not clear | Yes - Low Risk | *Some biomarker prevalence varies by stage or post treatments (EGFR NTRK resistance) so ideally sample is temporal with stage of cohort, although driver mutations like NTRK fusions often stable* |
|  |  |  | No - Potential Bias |  |
| *Source (Hoy Item 6)* | 6. Was an appropriate definition of the condition used? | Two Sub-Items to consider for this item   1. Was the study considering/assessing fusions across all three NTRK genes? 2. Was the study considering novel fusion partners? | Yes to both - Low Risk | *Relates to the methods used but using these items as a check that conceptually the methods can address these features, not how well they do it.* |
|  |  |  | No to Either - Potential Bias |  |
| *Source (JBI Item 6 & 7)* | 7. Were valid methods used for the identification of NTRK Fusions? | Two Sub-Items to consider for this item   1. Were NGS methods used for testing the entire cohort? 2. If yes, were RNA based methods or whole genome sequencing (WGS) methods used for the entire cohort | Yes to both - Ideal | *Methods allocated based on relative sensitivity & specificity for different methods ^b^* |
|  |  |  | Yes to a) only - Okay |  |
|  |  |  | No to a) - Poor |  |
| *Source (JBI Item 7 & Hoy Item 8)* | 8. Was the condition measured in a standard, reliable way for all participants? | YES   - The same test method was used for the entire cohort   NO   - Multiple versions of the same NGS panel used with differing NTRK fusion coverage - Different NGS assays were used for different patients - Different test methods used across cohort | Yes - Low Risk | *Relevant due to the potential differing accuracy/performance of different methods.*  *A multi-test algorithm applied consistently is considered the same method* |
|  |  |  | No - Potential Bias |  |
| *Source (Hoy Item 9)* | 9. Was the length of the shortest prevalence period for the parameter of interest appropriate? | YES   - Recruitment of cohort timeframe at least 12 months   NO   - Recruitment timeframe for cases unclear - Recruitment timeframe less than 12 months | Yes - Low Risk | *So rare that cross sectional prevalence is likely sufficient but ideally studies recruit population over 12 months to allow inference of annual incidence* |
|  |  |  | No - Potential Bias |  |
| *Source (JBI Item 8 & Hoy Item 10)* | 10. Were the numerator and denominator for prevalence appropriate? | YES   - Numerators/Denominators are clear and per patient   NO   - Prevalence rate reported without clarity on Numerator or Denominator - Non-Explicit Zero Fusion Study | Yes - Low Risk |  |
|  |  |  | No - Potential Bias |  |
| *Source (JBI Item 3)* | 11. Was the sample size adequate? | Minimum inclusion in cancer types is 50. (20 for rare cancer types)  Cancer types grouped into three categories of expected prevalence  (High >75%, Low 5-20%, Very Low 0.3-1%)  Sample sizes are scored in three tiers for each of the three groups:  **Ideal** (High >75, Low >245, Very Low >1280)  **Okay** (High 50-75, Low 70-245, Very Low 380-1280)  **Poor** (High <50, Low <70, Very Low <380) | Ideal | *ITEM to be performed per cancer type estimate not study.*  *Sample size calculations are based on single proportion formula ^c^*  *Expected prevalence in three tiers based on previous reviews ^d^* |
|  |  |  | Okay |  |
|  |  |  | Poor |  |
| *Source (JBI Item 4)* | 12. Were the study subjects described in detail?  *(Age/Sex/Stage/ Histology subtypes/ Ethnicity)* | YES   - Baseline characteristics of tested cohort described for at least 4 out of 5 demographics   NO   - 3 or less characteristics reported - Demographics not clear or not reported | Yes | *May not impact estimate but inability to compare cohort demographics across studies limit quality synthesis and discovery of enriched subgroups* |
|  |  |  | No |  |

^a^ Refers to the original item from the existing tool/s that was adapted for this context

^b^ Method criteria based on current literature [6-8] and consultation with pathologists at the Peter MacCallum Cancer Centre Department of Pathology, with higher sensitivity methods prioritised due to the rarity of NTRK fusions. For high prevalence cancers dominated by ETV6-NTRK3 fusions, it was acknowledged that the relative improvement in accuracy is likely less than other cancers, as more simple diagnostic methods would still detect the frequency of NTRK fusions with good accuracy with majority being ETV6-NTRK3, but evidence emerging still that secretory carcinomas and infantile fibrosarcomas may have fusions with alternative partners, so NGS is still preferable. RNA panels on WT cases by DNA panel capable of NTRK fusion detection were considered ideal under the assumption of mutual exclusion of NTRK fusions with other driver mutations and a lack of fusion indication on DNA panel being likely true negative.

^c^ Sample size calculation based on 95% confidence interval single proportion formula [9] and varying parameters of the formula (expected prevalence, sample size, precision and the Z statistic – in this case 1.96). These parameters are in supplementary table S9 below.

^d^ Expected prevalence categories and the included cancer types in each tier was based on current literature [10, 11].

# Development of sample size matrix for approval of sample sizes

Using the following formula for sample size for prevalence studies with 95% confidence:

$$N= \frac{Z^{2}* P(1-P))}{d^{2}}$$

Where $N$ = Sample Size, $P$ = Expected Prevalence, $d$ = Estimate Precision, $Z$ = 1.96

The following matrix of sample size ratings was used for assessing estimates relative sufficiency of sample size to detect NTRK fusions.

Supplementary Table S9. Sample size calculations for study appraisal across expected prevalence categories

| **Expected Prevalence Group** | **Included Cancer Types** | **Sample Size**  **Rating** | **Sample Size Range** | $\mathbf{P}$ | $\mathbf{d}$ | $\mathbf{N}$ |
| --- | --- | --- | --- | --- | --- | --- |
| High (>70%) | Secretory Carcinoma of the Salivary Gland, Secretory Breast Carcinoma, Infantile Fibrosarcoma | Ideal | ≥75 | 0.75 | 0.1 | 73 |
| High (>70%) | *As above* | Okay | ≥50 - <75 | 0.85 | 0.1 | 49 |
| High (>70%) | *As above* | Poor | <50 | . | . | . |
| Low (5%-20%) | Thyroid broadly or specifically Papillary Thyroid Carcinoma, Spitzoid Neoplasms, Glioma and glioma subtypes, Congenital Mesoblastic Nephroma | Ideal | ≥250 | 0.2 | 0.05 | 243 |
| Low (5%-20%) | *As above* | Okay | ≥75 - <250 | 0.2 | 0.1 | 62 |
| Low (5%-20%) | *As above* | Okay | ≥75 - <250 | 0.05 | 0.05 | 73 |
| Low (5%-20%) | *As above* | Poor | <75 | . | . | . |
| Very Low (≤1%) | All other types, including Pan cohorts | Ideal | ≥1280 | 0.003 | 0.003 | 1277 |
| Very Low (≤1%) | *As above* | Okay | ≥380 - <1280 | 0.01 | 0.01 | 381 |
| Very Low (≤1%) | *As above* | Poor | <380 | . | . | . |

# Algorithm for synthesis explained

The synthesis of data was done in two stages:

1. Broad narrative summary
2. Meta-analysis of high-quality estimates

First, all included studies with prevalence data extracted were combined into a narrative summary of estimates for the ‘pan cancer’ category and specific cancer cohorts which were collated within tumour groups. These tumour groups allowed for exploration of relative enrichment/variation of fusions in specific cancer types and provided the foundation for the bias and quality assessment (Figure S1).

Next, to derive robust prevalence estimates for each unique cancer type, we proceeded with rates that met additional synthesis criteria. Narratives were presented with tumours in groups and then specific types considered synonymous which were tallied for the number of types reported on and assessed for pooling.

For cancer types where no rates were eligible for pooling but fusions were identified, then the study with largest sample size is presented as a less robust estimate to highlight the presence of NTRK fusions in that cancer type (Figure S1).

Where studies reported rate but no numerator, this was estimated to come up with the confidence intervals.

When considering a unique cancer type with multiple estimates, reported rates were eligible for pooling and meta-analysis if the following synthesis criteria were satisfied (Figure S1):

1. No overlap with other cohort
2. NTRK fusion rates explicitly reported
3. Item 7. of the appraisal tool regarding valid methods used for identification of fusion ranked ‘Ideal’
4. Item 11. of the appraisal tool regarding appropriate sample size ranked ‘Good’ or ‘Okay’

Sensitivity analysis was performed if enough studies were eligible.

Supplementary Figure S1. Schema of Data Synthesis


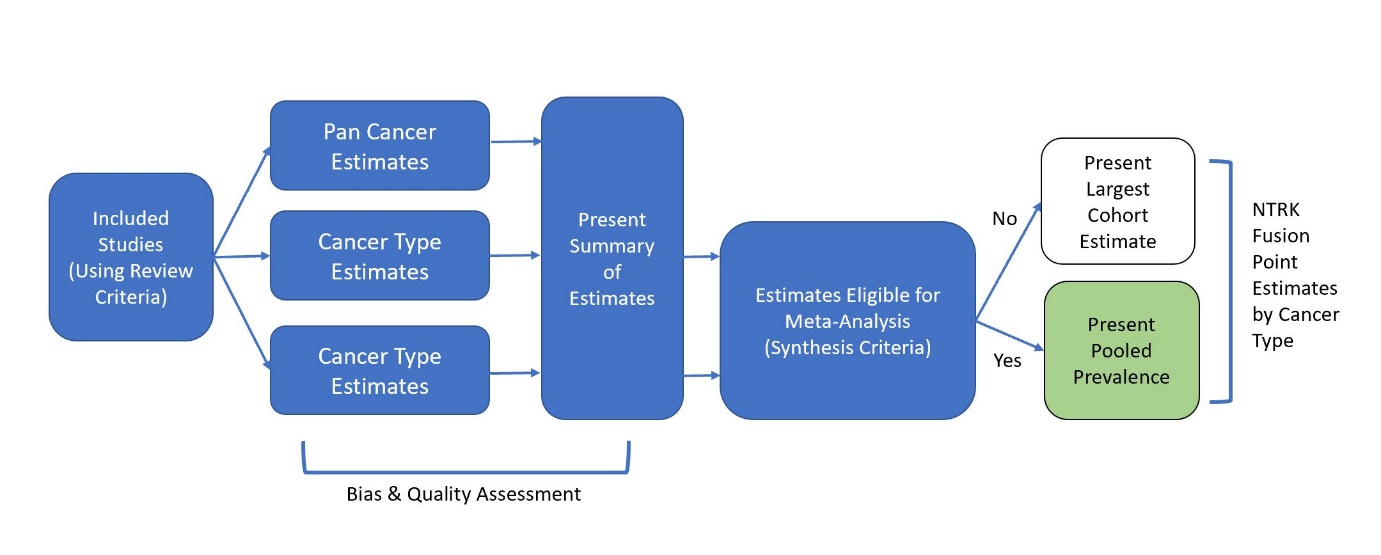


# Unique cancer types with no estimates eligible for pooling and no fusions identified

Supplementary Table S10. Unique cancer types without estimates eligible for pooling and no fusions identified

| **Cancer Type** | **# of estimates across review** | **Range across all point estimates in review** | **# of estimates eligible for pooling** | **NTRK Fusion Prevalence Point Estimate** | **95% Confidence Interval** | **Denominator** |
| --- | --- | --- | --- | --- | --- | --- |
| Anaplastic Oligodendroglioma | 1 | - | 0 | 0.00% | 0.00%-8.81% | 40 |
| Neuroendocrine Breast Cancer | 1 | - | 0 | 0.00% | 0.00%-16.84% | 20 |
| Anal Cancer | 1 | - | 0 | 0.00% | 0.00%-10.89% | 32 |
| Proficient MMR Colorectal Cancer | 1 | - | 0 | 0.00% | 0.00%-1.54% | 238 |
| RAS/BRAF WT, Anti-EGFR Sensitive Colorectal Cancer | 1 | - | 0 | 0.00% | 0.00%-7.55% | 47 |
| Rectal Adenocarcinoma | 2 | 0.00% | 0 | 0.00% | 0.00%-3.81% | 95 |
| Renal Cancer | 1 | - | 0 | 0.00% | 0.00%-3.03% | 12 |
| Kidney Chromophobe | 2 | 0.00% | 0 | 0.00% | 0.00%-5.44% | 66 |
| Kidney Papillary Cell Carcinoma | 2 | 0.00% | 0 | 0.00% | 0.00%-1.26% | 198 |
| Testicular Germ Cell Tumour | 1 | - | 0 | 0.00% | 0.00%-2.34% | 156 |
| Endometrial Carcinoma | 3 | 0.00% | 0 | 0.00% | 0.00%-1.73% | 185 |
| Uterine Carcinosarcoma | 1 | - | 0 | 0.00% | 0.00%-6.27% | 57 |
| Gynaecological | 1 | - | 0 | 0.00% | 0.00%-1.82% | 201 |
| Head & Neck | 2 | 0.00% | 0 | 0.00% | 0.00%-2.04% | 179 |
| Acinic Cell Carcinoma | 1 | - | 0 | 0.00% | 0.00%-4.93% | 73 |
| Adenoid Cystic Carcinoma | 1 | - | 0 | 0.00% | 0.00%-2.37% | 154 |
| Carcinoma ex Pleomorphic Adenoma | 1 | - | 0 | 0.00% | 0.00%-15.44% | 22 |
| Muco-Epidermoid Carcinoma | 1 | - | 0 | 0.00% | 0.00%-6.27% | 57 |
| Myo-epithelial Carcinoma | 1 | - | 0 | 0.00% | 0.00%-16.84 | 20 |
| Salivary Duct Carcinoma | 1 | - | 0 | 0.00% | 0.00%-8.04% | 44 |
| Salivary Gland Adenocarcinoma, NOS | 1 | - | 0 | 0.00% | 0.00%-3.10% | 117 |
| Salivary Gland Carcinoma, NOS | 1 | - | 0 | 0.00% | 0.00%-3.05% | 119 |
| Thoracic Primary | 1 | - | 0 | 0.00% | 0.00%-5.21% | 69 |
| Large Cell Carcinoma | 1 | - | 0 | 0.00% | 0.00%-6.06% | 59 |
| Pulmonary Neuroendocrine Tumour | 1 | - | 0 | 0.00% | 0.00%-2.37% | 154 |
| EGFR, KRAS, HER2, BRAF, MET, ALK, RET, ROS1, FGFR WT Lung Adenocarcinoma | 1 | - | 0 | 0.00% | 0.00%-2.06% | 177 |
| EGFRm Osimertinib Resistant Lung | 1 | - | 0 | 0.00% | 0.00%-5.78% | 62 |
| Inflammatory Myofibroblastic Tumours | 2 | 0.00%-5.00% | 0 | 0.00% | 0.00%-5.78% | 62 |
| Myxofibrosarcoma | 1 | - | 0 | 0.00% | 0.00%-11.94% | 29 |
| Desmoplastic Small Round Cell Tumors | 1 | - | 0 | 0.00% | 0.00%-4.35% | 83 |
| Skin | 1 | - | 0 | 0.00% | 0.00%-2.38% | 153 |
| Cutaneous Sarcomatoid Malignancies | 1 | - | 0 | 0.00% | 0.00%-4.93% | 73 |
| Acral Melanoma | 3 | 0.00% | 0 | 0.00% | 0.00%-4.35% | 83 |
| Uveal Melanoma | 2 | 0.00% | 0 | 0.00% | 0.00%-4.51% | 80 |
| Sun Protected Melanoma (Acral, Vulvar, Mucosal) | 1 | - | 0 | 0.00% | 0.00%-11.22% | 31 |
| Non-Melanoma Skin | 1 | - | 0 | 0.00% | 0.00%-2.53% | 144 |
| Melanoma Unknown Primary | 1 | - | 0 | 0.00% | 0.00%-3.10% | 117 |
| Follicular Variant Papillary Thyroid Cancer | 3 | 0.00%-13.33% | 0 | 0.00% | 0.00%-3.45% | 105 |
| Follicular Thyroid Cancer | 2 | 0.00% | 0 | 0.00% | 0.00%-5.52% | 65 |
| Medullary Thyroid Cancer | 1 | - | 0 | 0.00% | 0.00%-4.68% | 77 |
| Minimally Invasive Follicular Thyroid Cancer | 2 | 0.00% | 0 | 0.00% | 0.00%-11.57% | 30 |
| Focal Anaplastic/Poorly Differentiated Thyroid Cancer | 1 | - | 0 | 0.00% | 0.00%-12.34% | 28 |
| Upper Gastrointestinal | 1 | - | 0 | 0.00% | 0.00%-0.74% | 497 |
| Upper Gastrointestinal (Excluding Pancreas) | 1 | - | 0 | 0.00% | 0.00%-8.60% | 41 |
| Stomach Adenocarcinoma | 1 | - | 0 | 0.00% | 0.00%-1.29% | 285 |
| Esophagogastric Carcinoma | 1 | - | 0 | 0.00% | 0.00%-1.16% | 317 |
| Esophageal Carcinoma | 1 | - | 0 | 0.00% | 0.00%-1.97% | 185 |
| Esophagogastric Adenocarcinoma | 1 | - | 0 | 0.00% | 0.00%-0.84% | 438 |
| Hepatocellular Carcinoma | 6 | 0.00% | 0 | 0.00% | 0.00%-0.74% | 499 |
| Ampullary Cancer | 1 | - | 0 | 0.00% | 0.00%-13.72% | 25 |

# Prevalence Rates for All Specific Cancer Cohorts Extracted

Supplementary Table S11. NTRK Fusion Prevalence for Brain/Central Nervous System (CNS) Tumours

Studies included in meta-analysis are highlighted in blue.

| **Reference** | **YEAR** | **COUNTRY** | **OVERLAP** | **AGE** | **No. of positive cases detected** | **No of pts tested** | **% of cases detected** | **TYPE** | **# Per Unique Types** | **Eligible for pooling** | **Why not eligible?** | **Sample Size Ranking** | **Definition BIAS** | **Method Ranking** | **% NTRK1** | **% NTRK2** | **% NTRK3** |
| --- | --- | --- | --- | --- | --- | --- | --- | --- | --- | --- | --- | --- | --- | --- | --- | --- | --- |
| [12] | 2019 | USA | FM and MSK | Adult | 0 | 133 | 0.000% | Anaplastic Astrocytoma | 1 | Yes | . | Okay | Ideal | Okay |  |  |  |
| [12] | 2019 | USA | FM and MSK | Adult | 0 | 40 | 0.000% | Anaplastic Oligodendroglioma | 1 | No | Sample Size Ranking | Poor | Ideal | Okay |  |  |  |
| [13] | 2018 | Commercial Lab - NS | Caris Life Sciences | Adult | 7 | 316 | 2.215% | Astrocytoma | 1 | Yes | . | Ideal | Ideal | Ideal | 14.3% | 71.4% | 14.3% |
| [12] | 2019 | USA | FM and MSK | Adult | 0 | 104 | 0.000% | Diffuse Astrocytoma | 1 | Yes | . | Okay | Ideal | Okay |  |  |  |
| [14] | 2018 | International | N/A | Mixed | 3 | 30 | 10.000% | Diffuse Leptomeningeal Glioneuronal Tumour (DLGNT) | 1 | No | Sample Size and Method Ranking | Poor | Ideal | Poor | 33.3% | 33.3% | 33.3% |
| [15] | 2020 | South Korea | N/A | Adult | 5 | 166 | 3.01% | Glioblastoma | 10 | Yes | . | Okay | Ideal | Ideal |  |  |  |
| [16] | 2013 | USA | TCGA | Adult | 2 | 161 | 1.242% | Glioblastoma Multiforme | 10 | No | Larger TCGA cohort available | Okay | Ideal | Ideal | 100.0% | 0.0% | 0.0% |
| [17] | 2020 | Denmark | N/A | Adult | 1 | 108 | 0.93% | Glioblastoma Multiforme | 10 | Yes | . | Okay | Ideal | Ideal | 0.0% | 100.0% | 0.0% |
| [18] | 2020 | USA | MSK | Mixed | 4 | 641 | 0.624% | Glioblastoma Multiforme | 10 | Yes | . | Ideal | Ideal | Okay | 50.0% | 25.0% | 25.0% |
| [19] | 2013 | USA | N/A | NS | 0 | 24 | 0 | Glioblastoma Multiforme | 10 | No | Sample Size Ranking | Poor | Ideal | Ideal |  |  |  |
| [20] | 2014 | USA | TCGA | Adult | 1 | 157 | 0.637% | Glioblastoma Multiforme | 10 | No | Larger TCGA cohort available | Okay | Ideal | Ideal | 100.0% | 0.0% | 0.0% |
| [21] | 2019 | Brazil | N/A | Adult | 1 | 113 | 0.88% | Glioblastoma Multiforme | 10 | No | Method Ranking | Okay | Not Ideal | Poor | 100.0% | 0.0% | 0.0% |
| [22] | 2015 | USA | TCGA | Adult | 1 | 158 | 0.633% | Glioblastoma Multiforme | 10 | No | Larger TCGA cohort available | Okay | Ideal | Ideal | 100.0% | 0.0% | 0.0% |
| [12] | 2019 | USA | FM and MSK | Adult | 1 | 458 | 0.218% | Glioblastoma Multiforme | 10 | No | Larger MSK cohort available | Ideal | Ideal | Okay | 0.0% | 0.0% | 100.0% |
| [23] | 2018 | USA | TCGA | Adult | 1 | 180 | 0.556% | Glioblastoma Multiforme | 10 | Yes | . | Okay | Ideal | Ideal | 100.0% | 0.0% | 0.0% |
| [24] | 2019 | Commercial Lab - NS | Caris Life Sciences | Adult | 14 | 982 | 1.426% | Glioma | 3 | Yes | . | Ideal | Ideal | Ideal | 21.4% | 64.3% | 14.3% |
| [25] | 2017 | USA | MSK | Mixed | 1 | 512 | 0.195% | Glioma | 3 | Yes | . | Ideal | Ideal | Okay | 0.0% | 0.0% | 100.0% |
| [26] | 2020 | Commercial Lab - NS | FM | Adult | 21 | 6395 | 0.328% | Glioma | 3 | Yes | . | Ideal | Ideal | Okay | NS | NS | NS |
| [13] | 2018 | Commercial Lab - NS | Caris Life Sciences | Adult | 1 | 41 | 2.439% | Glioma NOS | 1 | No | Method Ranking | Poor | Ideal | Poor | 0.0% | 100.0% | 0.0% |
| [27] | 2020 | USA | MSK | Mixed | 8 | 1465 | 0.546% | Glioma/Neuroepithelial tumour | 1 | Yes | . | Ideal | Ideal | Okay | 37.5% | 37.5% | 25.0% |
| [20] | 2014 | USA | TCGA | Adult | 2 | 461 | 0.434% | Low Grade Glioma | 3 | No | Larger TCGA cohort available | Ideal | Ideal | Ideal | 0.0% | 100.0% | 0.0% |
| [22] | 2015 | USA | TCGA | Adult | 2 | 266 | 0.752% | Low Grade Glioma | 3 | No | Larger TCGA cohort available | Ideal | Ideal | Ideal | 50.0% | 50.0% | 0.0% |
| [23] | 2018 | USA | TCGA | Adult | 5 | 534 | 0.936% | Low Grade Glioma | 3 | Yes |  | Ideal | Ideal | Ideal | 20.0% | 60.0% | 20.0% |
| [13] | 2018 | Commercial Lab - NS | Caris Life Sciences | Adult | 0 | 33 | 0.000% | Oligodendroglioma | 2 | No | Sample Size Ranking | Poor | Ideal | Poor |  |  |  |
| [12] | 2019 | USA | FM and MSK | Adult | 0 | 88 | 0.000% | Oligodendroglioma | 2 | Yes |  | Okay | Ideal | Okay |  |  |  |
| [26] | 2020 | Commercial Lab - NS | FM | Adult | 3 | 1386 | 0.216% | Unknown Neurological Primary | 1 | Yes | . | Ideal | Ideal | Okay | NS | NS | NS |

Supplementary Table S12. NTRK Fusion Prevalence for Breast Cancers

Studies included in meta-analysis are highlighted in blue.

| **Reference** | **YEAR** | **COUNTRY** | **OVERLAP** | **AGE** | **COHORT STAGE** | **No. of positive cases detected** | **No of pts tested** | **% of cases detected** | **TYPE** | **# Per Unique Types** | **Eligible for pooling** | **Why not eligible?** | **Sample Size Ranking** | **Definition BIAS** | **Method Ranking** | **% NTRK1** | **% NTRK2** | **% NTRK3** |
| --- | --- | --- | --- | --- | --- | --- | --- | --- | --- | --- | --- | --- | --- | --- | --- | --- | --- | --- |
| [28] | 2020 | Germany | N/A | Adult | Advanced | 0 | 149 | 0.0% | Breast | 13 | No | Non-Explicit Zero | Poor | Ideal | Ideal |  |  |  |
| [29] | 2021 | Canada | POG Canada | Adult | Advanced | 1 | 149 | 0.671% | Breast | 13 | No | Sample Size Ranking | Poor | Ideal | Ideal | 100.0% | 0.0% | 0.0% |
| [30] | 2020 | Italy | N/A | Unclear | NS | 2 | 711 | 0.281% | Breast | 13 | No | Method Ranking | Okay | Ideal | Poor | 100.0% | 0.0% | 0.0% |
| [31] | 2021 | China | N/A | Adult | Mixed | 0 | 874 | 0.000% | Breast | 13 | No | Method Ranking | Okay | Ideal | Poor |  |  |  |
| [22] | 2015 | USA | TCGA | Adult | Early | 1 | 1,019 | 0.098% | Breast Cancer | 13 | No | Larger TCGA Available | Okay | Ideal | Ideal | 0.0% | 0.0% | 100.0% |
| [23] | 2018 | USA | TCGA | Adult | NS | 2 | 1119 | 0.179% | Breast Cancer | 13 | Yes |  | Okay | Ideal | Ideal | 0.0% | 50.0% | 50.0% |
| [25] | 2017 | USA | MSK | Mixed | Advanced | 0 | 1237 | 0.000% | Breast Cancer | 13 | No | Larger MSK Cohort Available | Okay | Ideal | Okay |  |  |  |
| [26] | 2020 | Commercial Lab - NS | FM | Adult | NS | 44 | 19024 | 0.231% | Breast Cancer | 13 | Yes |  | Ideal | Ideal | Okay |  |  |  |
| [24] | 2019 | Commercial Lab - NS | Caris Life Sciences | Adult | NS | 1 | 769 | 0.130% | Breast Carcinoma (Includes Secretory) | 13 | Yes |  | Okay | Ideal | Ideal | 0.0% | 0.0% | 100.0% |
| [27] | 2020 | USA | MSK | Mixed | NS - MSK Advanced | 6 | 4458 | 0.135% | Breast Carcinoma (Includes Secretory) | 13 | Yes |  | Ideal | Ideal | Okay | 33.3% | 0.0% | 66.7% |
| [20] | 2014 | USA | TCGA | Adult | Mixed | 1 | 1072 | 0.093% | Invasive Breast Carcinoma | 13 | No | Larger TCGA Available | Okay | Ideal | Ideal | 0.0% | 0.0% | 100.0% |
| [32] | 2015 | South Korea | N/A | Adult | Early | 1 | 120 | 0.833% | Invasive Breast Carcinoma (Includes Secretory) | 13 | No | Sample Size Ranking | Poor | Ideal | Ideal | 0.0% | 0.0% | 100.0% |
| [33] | 2019 | France | N/A | Adult | NS | 1 | 339 | 0.3% | Invasive Breast Carcinoma | 13 | No | Sample Size Ranking | Poor | Ideal | Poor | 100.0% | 0.0% | 0.0% |
| [34] | 2020 | USA | MSK | Adult | Advanced | 4 | 4854 | 0.08% | Breast Cancer (Excludes Secretory) | 2 | Yes |  | Ideal | Ideal | Okay | 100.0% | 0.0% | 0.0% |
| [18] | 2020 | USA | MSK | Mixed | NS - MSK Advanced | 3 | 3775 | 0.079% | Invasive Breast Carcinoma (Excludes Secretory) | 2 | No | Larger MSK Cohort Available | Ideal | Ideal | Okay | 66.7% | 0.0% | 33.3% |
| [35] | 2019 | Commercial Lab - NS | Caris Life Sciences | Adult | Early | 0 | 20 | 0 | Neuroendocrine of the Breast | 1 | No | Sample and Method Ranking | Poor | Ideal | Poor |  |  |  |
| [36] | 2019 | China | N/A | Mixed | Early | 39 | 44 | 88.64% | Secretory Carcinoma of the Breast | 1 | No | Sample and Method Ranking | Poor | Not Ideal | Poor | 0.0% | 0.0% | 100.0% |

Supplementary Table S13. NTRK Fusion Prevalence for Cancer of Unknown Primary

Studies included in meta-analysis are highlighted in blue.

| **Reference** | **YEAR** | **COUNTRY** | **OVERLAP** | **AGE** | **COHORT STAGE** | **No. of positive cases detected** | **No of pts tested** | **% of cases detected** | **TYPE** | **# Per Unique Types** | **Eligible for pooling** | **Why not eligible?** | **Sample Size Ranking** | **Definition BIAS** | **Method Ranking** | **% NTRK1** | **% NTRK2** | **% NTRK3** |
| --- | --- | --- | --- | --- | --- | --- | --- | --- | --- | --- | --- | --- | --- | --- | --- | --- | --- | --- |
| [25] | 2017 | USA | MSK | Mixed | Advanced | 1 | 160 | 0.625% | Cancer of Unknown Primary | 4 | No | Larger MSK Cohort Available | Poor | Ideal | Okay | 100.0% | 0.0% | 0.0% |
| [24] | 2019 | Commercial Lab - NS | Caris Life Sciences | Adult | NS | 2 | 227 | 0.881% | Cancer of Unknown Primary | 4 | No | Sample Size Ranking | Poor | Ideal | Ideal | 0.0% | 0.0% | 100.0% |
| [26] | 2020 | Commercial Lab - NS | FM | Adult | NS | 15 | 10636 | 0.141% | Cancer of Unknown Primary | 4 | Yes |  | Ideal | Ideal | Okay |  |  |  |
| [18] | 2020 | USA | MSK | Mixed | NS - MSK Advanced | 1 | 318 | 0.314% | Unknown Primary | 4 | No | Sample Size Ranking | Poor | Ideal | Okay | 100.0% | 0.0% | 0.0% |

Supplementary Table S14. NTRK Fusion Prevalence for Colorectal Cancers

Studies included in meta-analysis are highlighted in blue.

| **Reference** | **YEAR** | **COUNTRY** | **OVERLAP** | **AGE** | **COHORT STAGE** | **No. of positive cases detected** | **No of pts tested** | **% of cases detected** | **TYPE** | **# Per Unique Types** | **Eligible for pooling** | **Why not eligible?** | **Sample Size Ranking** | **Definition BIAS** | **Method Ranking** | **% NTRK1** | **% NTRK2** | **% NTRK3** |
| --- | --- | --- | --- | --- | --- | --- | --- | --- | --- | --- | --- | --- | --- | --- | --- | --- | --- | --- |
| [26] | 2020 | Commercial Lab - NS | FM | Adult | NS | 52 | 24747 | 0.210% | Colorectal | 16 | Yes |  | Ideal | Ideal | Okay |  |  |  |
| [37] | 2021 | China | Cancer Sequencing YS Panel | Adult | Mixed | 3 | 630 | 0.476% | Colorectal Cancer | 16 | Yes |  | Okay | Ideal | Okay | 100.0% | 0.0% | 0.0% |
| [24] | 2019 | Commercial Lab - NS | Caris Life Sciences | Adult | NS | 2 | 1272 | 0.157% | Colorectal Carcinoma | 16 | Yes |  | Okay | Ideal | Ideal | 100.0% | 0.0% | 0.0% |
| [27] | 2020 | USA | MSK | Mixed | NS - MSK Advanced | 9 | 2929 | 0.307% | Colorectal Carcinoma | 16 | Yes |  | Ideal | Ideal | Okay | 66.7% | 0.0% | 33.3% |
| [38] | 2015 | France | N/A | Mixed | Mixed | 1 | 408 | 0.25% | Colorectal Carcinoma | 1 | No | Sample Size Ranking | Poor | Poor | Poor | 0.25% | 0.0% | 0.0% |
| [29] | 2021 | Canada | POG Canada | Adult | Advanced | 0 | 73 | 0.000% | Colorectal | 16 | No | Sample Size Ranking | Poor | Ideal | Ideal |  |  |  |
| [39] | 2018 | USA | MSK | Adult | Advanced | 5 | 979 | 0.51% | Colorectal | 16 | No | Larger MSK Cohort Available | Okay | Ideal | Okay | 80.0% | 0.0% | 20.0% |
| [31] | 2021 | China | N/A | Adult | Mixed | 2 | 1007 | 0.199% | Colorectal | 16 | No | Method Ranking | Okay | Ideal | Poor |  |  |  |
| [40] | 2015 | South Korea | Samsung Medical Center | Adult | Advanced | 2 | 74 | 2.70% | Colorectal Cancer | 16 | No | Method & Sample Size Ranking | Poor | Not Ideal | Poor | 100.0% | 0.0% | 0.0% |
| [41] | 2018 | South Korea | N/A | Adult | NS | 6 | 80 | 7.50% | Colorectal Cancer | 16 | No | Method & Sample Size Ranking | Poor | Not Ideal | Poor | 100.0% | 0.0% | 0.0% |
| [42] | 2020 | Taiwan | N/A | Adult | Mixed | 1 | 104 | 0.96% | Colorectal Cancer | 16 | No | Sample Size Ranking | Poor | Ideal | Ideal | 100.0% | 0.0% | 0.0% |
| [43] | 2018 | Italy | N/A | NS | Advanced | 1 | 550 | 0.18% | Colorectal Cancer | 16 | No | Method Ranking | Okay | Not Ideal | Poor | 100.0% | 0.0% | 0.0% |
| [44] | 2020 | China | Cancer Sequencing YS Panel | Adult | Mixed | 6 | 609 | 0.985% | Colorectal Cancer | 16 | No | Larger CSYS Cohort Available | Okay | Ideal | Okay | 66.7% | 0.0% | 33.3% |
| [25] | 2017 | USA | MSK | Mixed | Advanced | 3 | 978 | 0.307% | Colorectal Cancer | 16 | No | Larger MSK Cohort Available | Okay | Ideal | Okay | 66.7% | 0.0% | 33.3% |
| [45] | 2021 | Japan | N/A | Adult | NS | 2 | 1012 | 0.20% | Colorectal Cancer | 16 | No | Method Ranking | Okay | Ideal | Poor | 100.0% | 0.0% | 0.0% |
| [46] | 2019 | USA | MSK | Adult | Advanced | 8 | 2314 | 0.346% | Colorectal Cancer | 16 | No | Larger MSK Cohort Available | Ideal | Ideal | Ideal | 75.0% | 0.0% | 25.0% |
| [47] | 2019 | USA | Guardant 360 | Adult | Advanced | 3 | 3808 | 0.08% | Colorectal Cancer | 16 | No | Method Ranking | Ideal | Not Ideal | Poor | 100.0% | 0.0% | 0.0% |
| [48] | 2017 | The Netherlands | N/A | Adult | Early | 1 | 278 | 0.360% | Colon Adenocarcinoma | 4 | No | Sample Size Ranking | Poor | Ideal | Ideal | 0.0% | 0.0% | 100.0% |
| [20] | 2014 | USA | TCGA | Adult | Mixed | 2 | 286 | 0.699% | Colon Adenocarcinoma | 4 | No | Sample Size Ranking | Poor | Ideal | Ideal | 0.0% | 0.0% | 100.0% |
| [23] | 2018 | USA | TCGA | Adult | NS - TCGA | 3 | 310 | 0.968% | Colon Adenocarcinoma | 4 | No | Sample Size Ranking | Poor | Ideal | Ideal | 0.0% | 0.0% | 100.0% |
| [49] | 2020 | International | N/A | Infer (Adult) | NS | 16 | 7008 | 0.228% | Colonic Adenocarcinoma | 4 | No | Method Ranking | Ideal | Ideal | Poor | 93.3% | 0.0% | 6.7% |
| [50] | 2021 | Japan | FM | Adult | Advanced | 1 | 60 | 1.667% | Colorectal Adenocarcinoma | 4 | No | Sample Size Ranking | Poor | Ideal | Okay | 100.0% | 0.0% | 0.0% |
| [39] | 2018 | USA | MSK | Adult | Early | 0 | 120 | 0.000% | Colorectal Adenocarcinoma | 4 | No | Sample Size Ranking | Poor | Ideal | Okay |  |  |  |
| [51] | 2019 | South Korea | Samsung Medical Center | Mixed | Advanced | 2 | 181 | 1.105% | Colorectal Adenocarcinoma | 4 | No | Sample Size Ranking | Poor | Ideal | Ideal |  |  |  |
| [52] | 2020 | Australia | N/A | Adult | Early | 9 | 4569 | 0.20% | Colorectal Adenocarcinoma | 4 | No | Method Ranking | Ideal | Ideal | Poor | 88.9% | 11.1% | 0.0% |
| [18] | 2020 | USA | MSK | Mixed | NS - MSK Advanced | 8 | 2306 | 0.347% | Colon Cancer | 2 | Yes |  | Ideal | Ideal | Okay | 62.5% | 0.0% | 37.5% |
| [18] | 2020 | USA | MSK | Mixed | NS - MSK Advanced | 1 | 176 | 0.568% | Appendiceal Adenocarcinoma | 2 | No | Sample Size Ranking | Poor | Ideal | Okay | 100.0% | 0.0% | 0.0% |
| [27] | 2020 | USA | MSK | Mixed | NS - MSK Advanced | 1 | 208 | 0.481% | Appendiceal Adenocarcinoma | 2 | No | Sample Size Ranking | Poor | Ideal | Okay | 100.0% | 0.0% | 0.0% |
| [53] | 2016 | South Korea | N/A | Adult | Early | 3 | 147 | 2.04% | Colon Cancer | 2 | No | Sample Size Ranking | Poor | Ideal | Ideal | 100.0% | 0.0% | 0.0% |
| [23] | 2018 | USA | TCGA | Adult | NS - TCGA | 0 | 95 | 0.000% | Rectal Adenocarcinoma | 2 | No | Sample Size Ranking | Poor | Ideal | Ideal |  |  |  |
| [20] | 2014 | USA | TCGA | Adult | Mixed | 0 | 91 | 0.000% | Rectum Adenocarcinoma | 2 | No | Sample Size Ranking | Poor | Ideal | Ideal |  |  |  |
| [54] | 2019 | Commercial Lab - NS | FM | Infer (adult) | NS | NS | NS | 3.250% | Microsatellite Instability High Colorectal Carcinoma | 1 | Yes |  | Ideal | Ideal | Okay |  |  |  |
| [54] | 2019 | Commercial Lab - NS | FM | Infer (adult) | NS | NS | NS | 0.18% | Microsatellite Stable Colorectal Carcinoma | 1 | Yes |  | Ideal | Ideal | Okay |  |  |  |
| [25] | 2017 | USA | MSK | Mixed | Advanced | 0 | 32 | 0.000% | Anal Cancer | 1 | No | Sample Size Ranking | Poor | Ideal | Okay |  |  |  |
| [25] | 2017 | USA | MSK | Mixed | Advanced | 1 | 79 | 1.266% | Appendiceal Cancer | 1 | No | Sample Size Ranking | Poor | Ideal | Okay | 100.0% | 0.0% | 0.0% |
| [55] | 2019 | China | N/A | Adult | Mixed | 0 | 238 | 0.000% | Proficient Mismatch Repair Colorectal Cancer | 1 | No | Sample Size Ranking | Poor | Ideal | Okay |  |  |  |
| [55] | 2019 | China | N/A | Adult | Mixed | 7 | 125 | 5.60% | Deficient Mismatch Repair Colorectal Carcinoma | 1 | No | Sample Size Ranking | Poor | Ideal | Okay | 71.4% | 0.0% | 28.6% |
| [56] | 2019 | Italy | PRESSING Panel | Adult | Advanced | 1 | 199 | 0.50% | RAS/BRAF WT Colorectal Cancer | 1 | No | Method & Sample Size Ranking | Poor | Ideal | Poor | 0.0% | 0.0% | 100.0% |
| [57] | 2017 | Italy | PRESSING Panel | Adult | Advanced | 0 | 47 | 0.000% | RAS/BRAF WT, anti-EGFR Sensitive (Disease Control >6 Months) Colorectal Cancer | 1 | No | Method & Sample Size Ranking | Poor | Ideal | Poor |  |  |  |

Supplementary Table S15. NTRK Fusion Prevalence for Genitourinary Cancers

Studies included in meta-analysis are highlighted in blue.

| **Reference** | **YEAR** | **COUNTRY** | **OVERLAP** | **AGE** | **COHORT STAGE** | **No. of positive cases detected** | **No of pts tested** | **% of cases detected** | **TYPE** | **# Per Unique Types** | **Eligible for pooling** | **Why not eligible?** | **Sample Size Ranking** | **Definition BIAS** | **Method Ranking** | **% NTRK1** | **% NTRK2** | **% NTRK3** |
| --- | --- | --- | --- | --- | --- | --- | --- | --- | --- | --- | --- | --- | --- | --- | --- | --- | --- | --- |
| [31] | 2021 | China | N/A | Adult | Mixed | 0 | 53 | 0.000% | Prostate | 5 | No | Method & Sample Size Ranking | Poor | Ideal | Poor |  |  |  |
| [58] | 2019 | USA | FM | NS | NS | 0 | 67 | 0.000% | Prostate | 5 | No | Larger FM Cohort Available | Poor | Ideal | Okay |  |  |  |
| [30] | 2020 | Italy | N/A | Unclear | NS | 0 | 177 | 0.000% | Prostate | 5 | No | Method & Sample Size Ranking | Poor | Ideal | Poor |  |  |  |
| [25] | 2017 | USA | MSK | Mixed | Advanced | 0 | 623 | 0.000% | Prostate Cancer | 5 | Yes |  | Okay | Ideal | Okay |  |  |  |
| [26] | 2020 | Commercial Lab - NS | FM | Adult | NS | 11 | 7222 | 0.152% | Prostate Cancer | 5 | Yes |  | Ideal | Ideal | Okay |  |  |  |
| [23] | 2018 | USA | TCGA | Adult | NS - TCGA | 0 | 502 | 0.000% | Prostate Adenocarcinoma | 3 | Yes |  | Okay | Ideal | Ideal |  |  |  |
| [22] | 2015 | USA | TCGA | Adult | Early | 0 | 178 | 0.000% | Prostate Adenocarcinoma | 3 | No | Larger TCGA Cohort Available | Poor | Ideal | Ideal |  |  |  |
| [20] | 2014 | USA | TCGA | Adult | Mixed | 0 | 335 | 0.000% | Prostate Adenocarcinoma | 3 | No | Larger TCGA Cohort Available | Poor | Ideal | Ideal |  |  |  |
| [31] | 2021 | China | N/A | Adult | Mixed | 0 | 78 | 0.000% | Bladder | 4 | No |  | Poor | Ideal | Poor |  |  |  |
| [30] | 2020 | Italy | N/A | Unclear | NS | 0 | 129 | 0.000% | Bladder | 4 | No |  | Poor | Ideal | Poor |  |  |  |
| [25] | 2017 | USA | MSK | Mixed | Advanced | 0 | 406 | 0.000% | Bladder Cancer | 4 | Yes |  | Okay | Ideal | Okay |  |  |  |
| [26] | 2020 | Commercial Lab - NS | FM | Adult | NS | 8 | 3425 | 0.234% | Bladder Cancer | 4 | Yes |  | Ideal | Ideal | Okay |  |  |  |
| [23] | 2018 | USA | TCGA | Adult | NS - TCGA | 0 | 414 | 0.000% | Bladder Urothelial Carcinoma | 3 | Yes |  | Okay | Ideal | Ideal |  |  |  |
| [22] | 2015 | USA | TCGA | Adult | Early | 0 | 121 | 0.000% | Bladder Urothelial Carcinoma | 3 | No | Larger TCGA Cohort Available | Poor | Ideal | Ideal |  |  |  |
| [20] | 2014 | USA | TCGA | Adult | Mixed | 0 | 250 | 0.000% | Bladder Urothelial Carcinoma | 3 | No | Larger TCGA Cohort Available | Poor | Ideal | Ideal |  |  |  |
| [31] | 2021 | China | N/A | Adult | Mixed | 0 | 120 | 0.000% | Renal | 1 | No | Method & Sample Size Ranking | Poor | Ideal | Poor |  |  |  |
| [25] | 2017 | USA | MSK | Mixed | Advanced | 0 | 322 | 0.000% | Renal Cell Carcinoma | 1 | No | Sample Size Ranking | Poor | Ideal | Okay |  |  |  |
| [23] | 2018 | USA | TCGA | Adult | NS - TCGA | 0 | 541 | 0.000% | Renal Clear Cell Carcinoma | 3 | Yes |  | Okay | Ideal | Ideal |  |  |  |
| [22] | 2015 | USA | TCGA | Adult | Early | 0 | 474 | 0.000% | Renal Clear Cell Carcinoma | 3 | No | Larger TCGA Cohort Available | Okay | Ideal | Ideal |  |  |  |
| [20] | 2014 | USA | TCGA | Adult | Mixed | 0 | 529 | 0.000% | Kidney Clear Cell Carcinoma | 3 | No | Larger TCGA Cohort Available | Okay | Ideal | Ideal |  |  |  |
| [20] | 2014 | USA | TCGA | Adult | Mixed | 0 | 66 | 0.000% | Kidney Chromophobe | 2 | No | Sample Size Ranking | Poor | Ideal | Ideal |  |  |  |
| [23] | 2018 | USA | TCGA | Adult | NS - TCGA | 0 | 66 | 0.000% | Renal Chromophobe Tumor | 2 | No | Sample Size Ranking | Poor | Ideal | Ideal |  |  |  |
| [20] | 2014 | USA | TCGA | Adult | Mixed | 0 | 198 | 0.000% | Kidney Papillary Cell Carcinoma | 2 | No | Sample Size Ranking | Poor | Ideal | Ideal |  |  |  |
| [23] | 2018 | USA | TCGA | Adult | NS - TCGA | 0 | 291 | 0.000% | Renal Papillary Cell Carcinoma | 2 | No | Sample Size Ranking | Poor | Ideal | Ideal |  |  |  |
| [59] | 2020 | International | N/A | Mixed | Early | 2 | 20 | 10% | Metanephric Adenomas | 1 | No | Method & Sample Size Ranking | Poor | Not Ideal | Poor | 100.0% | 0.0% | 0.0% |
| [23] | 2018 | USA | TCGA | Adult | NS - TCGA | 0 | 156 | 0.000% | Testicular Germ Cell Tumours | 1 | No | Sample Size Ranking | Poor | Ideal | Ideal |  |  |  |

Supplementary Table S16. NTRK Fusion Prevalence for Gynaecological Cancers

Studies included in meta-analysis are highlighted in blue.

| **Reference** | **YEAR** | **COUNTRY** | **OVERLAP** | **AGE** | **COHORT STAGE** | **No. of positive cases detected** | **No of pts tested** | **% of cases detected** | **TYPE** | **# Per Unique Types** | **Eligible for pooling** | **Why not eligible?** | **Sample Size Ranking** | **Definition BIAS** | **Method Ranking** | **% NTRK1** | **% NTRK2** | **% NTRK3** |
| --- | --- | --- | --- | --- | --- | --- | --- | --- | --- | --- | --- | --- | --- | --- | --- | --- | --- | --- |
| [31] | 2021 | China | N/A | Adult | Mixed | 0 | 104 | 0.000% | Cervical | 4 | No | Method & Sample Size Ranking | Poor | Ideal | Poor |  |  |  |
| [25] | 2017 | USA | MSK | Mixed | Advanced | 0 | 47 | 0.000% | Cervical Cancer | 4 | No | Sample Size Ranking | Poor | Ideal | Okay |  |  |  |
| [23] | 2018 | USA | TCGA | Adult | NS - TCGA | 1 | 306 | 0.327% | Cervical Cancer | 4 | No | Sample Size Ranking | Poor | Ideal | Ideal | 0.0% | 0.0% | 100.0% |
| [24] | 2019 | Commercial Lab - NS | Caris Life Sciences | Adult | NS | 1 | 68 | 1.471% | Cervical Carcinoma | 4 | No | Sample Size Ranking | Poor | Ideal | Ideal | 100.0% | 0.0% | 0.0% |
| [25] | 2017 | USA | MSK | Mixed | Advanced | 0 | 211 | 0.000% | Endometrial (Excludes Uterine Sarcoma) | 3 | No | Sample Size Ranking | Poor | Ideal | Okay |  |  |  |
| [20] | 2014 | USA | TCGA | Adult | Mixed | 0 | 166 | 0.000% | Endometrial Carcinoma | 3 | No | Larger TCGA Cohort Available | Poor | Ideal | Ideal |  |  |  |
| [23] | 2018 | USA | TCGA | Adult | NS - TCGA | 0 | 185 | 0.000% | Endometrial Carcinoma | 3 | No | Sample Size Ranking | Poor | Ideal | Ideal |  |  |  |
| [26] | 2020 | Commercial Lab - NS | FM | Adult | NS | 3 | 1078 | 0.278% | Fallopian Tube | 1 | No | Sample Size Ranking | Poor | Ideal | Okay |  |  |  |
| [30] | 2020 | Italy | N/A | Unclear | NS | 0 | 201 | 0.000% | Gynaecological | 2 | No | Method & Sample Size Ranking | Poor | Ideal | Poor |  |  |  |
| [29] | 2021 | Canada | POG Canada | Adult | Advanced | 0 | 26 | 0.000% | Gynaecological | 2 | No | Sample Size Ranking | Poor | Ideal | Ideal |  |  |  |
| [60] | 2020 | Commercial Lab - NS | Caris Life Sciences | Adult | Mixed | 0 | 62 | 0.000% | Neuroendocrine Carcinoma of the Cervix | 1 | No | Sample Size Ranking | Poor | Ideal | Ideal |  |  |  |
| [29] | 2021 | Canada | POG Canada | Adult | Advanced | 0 | 28 | 0.000% | Ovarian | 4 | No | Sample Size Ranking | Poor | Ideal | Ideal |  |  |  |
| [25] | 2017 | USA | MSK | Mixed | Advanced | 0 | 217 | 0.000% | Ovarian Cancer | 4 | No | Sample Size Ranking | Poor | Ideal | Okay |  |  |  |
| [23] | 2018 | USA | TCGA | Adult | NS - TCGA | 0 | 428 | 0.000% | Ovarian Serous Carcinoma | 3 | Yes |  | Okay | Ideal | Ideal |  |  |  |
| [22] | 2015 | USA | TCGA | Adult | Early | 0 | 400 | 0.000% | Ovarian Serous Cystadenocarcinoma | 3 | No | Larger TCGA Cohort Available | Okay | Ideal | Ideal |  |  |  |
| [20] | 2014 | USA | TCGA | Adult | Mixed | 0 | 412 | 0.000% | Ovarian Serous Cystadenocarcinoma | 3 | No | Larger TCGA Cohort Available | Okay | Ideal | Ideal |  |  |  |
| [31] | 2021 | China | N/A | Adult | Mixed | 0 | 190 | 0.000% | Ovary | 4 | No | Method & Sample Size Ranking | Poor | Ideal | Poor |  |  |  |
| [26] | 2020 | Commercial Lab - NS | FM | Adult | NS | 21 | 11590 | 0.181% | Ovary | 4 | Yes |  | Ideal | Ideal | Okay |  |  |  |
| [31] | 2021 | China | N/A | Adult | Mixed | 0 | 64 | 0.000% | Uterine | 2 | No | Method & Sample Size Ranking | Poor | Ideal | Poor |  |  |  |
| [23] | 2018 | USA | TCGA | Adult | NS - TCGA | 0 | 57 | 0.000% | Uterine Carcinosarcoma | 1 | No | Sample Size Ranking | Poor | Ideal | Ideal |  |  |  |
| [25] | 2017 | USA | MSK | Mixed | Advanced | 0 | 91 | 0.000% | Uterine Sarcoma | 2 | No | Sample Size Ranking | Poor | Ideal | Okay |  |  |  |
| [18] | 2020 | USA | MSK | Mixed | NS - MSK Advanced | 2 | 174 | 1.149% | Uterine Sarcoma | 2 | No | Sample Size Ranking | Poor | Ideal | Okay | 100.0% | 0.0% | 0.0% |
| [26] | 2020 | Commercial Lab - NS | FM | Adult | NS | 2 | 1080 | 0.185% | Uterus | 2 | Yes |  | Okay | Ideal | Okay |  |  |  |

Supplementary Table S17. NTRK Fusion Prevalence for Head & Neck Cancers

Studies included in meta-analysis are highlighted in blue.

| **Reference** | **YEAR** | **COUNTRY** | **OVERLAP** | **AGE** | **COHORT STAGE** | **No. of positive cases detected** | **No of pts tested** | **% of cases detected** | **TYPE** | **# Per Unique Types** | **Eligible for pooling** | **Why not eligible?** | **Sample Size Ranking** | **Definition BIAS** | **Method Ranking** | **% NTRK1** | **% NTRK2** | **% NTRK3** |
| --- | --- | --- | --- | --- | --- | --- | --- | --- | --- | --- | --- | --- | --- | --- | --- | --- | --- | --- |
| [26] | 2020 | Commercial Lab - NS | FM | Adult | NS | 3 | 3145 | 0.095% | Head and Neck (Excluding Salivary Gland Cancers) | 1 | Yes |  | Ideal | Ideal | Okay |  |  |  |
| [61] | 2017 | Commercial Lab - NS | FM | Adult | NS | 0 | 73 | 0.00% | Acinic Cell Carcinoma | 1 | No | Sample Size Ranking | Poor | Ideal | Okay |  |  |  |
| [61] | 2017 | Commercial Lab - NS | FM | Adult | NS | 0 | 154 | 0.00% | Adenoid Cystic Carcinoma | 1 | No | Sample Size Ranking | Poor | Ideal | Okay |  |  |  |
| [61] | 2017 | Commercial Lab - NS | FM | Adult | NS | 0 | 22 | 0.00% | Carcinoma ex Pleomorphic Adenoma | 1 | No | Sample Size Ranking | Poor | Ideal | Okay |  |  |  |
| [31] | 2021 | China | N/A | Adult | Mixed | 0 | 97 | 0.000% | Head and Neck | 2 | No | Method and Sample Size Ranking | Poor | Ideal | Poor |  |  |  |
| [25] | 2017 | USA | MSK | Mixed | Advanced | 0 | 179 | 0.000% | Head and Neck | 2 | No | Sample Size Ranking | Poor | Ideal | Okay |  |  |  |
| [22] | 2015 | USA | TCGA | Adult | Early | 1 | 300 | 0.333% | Head and Neck Squamous Cell Carcinoma | 3 | No | Sample Size Ranking | Poor | Ideal | Ideal | 0.0% | 0.0% | 100.0% |
| [20] | 2014 | USA | TCGA | Adult | Mixed | 2 | 411 | 0.487% | Head and Neck Squamous Cell Carcinoma | 3 | No | Larger TCGA Cohort Available | Okay | Ideal | Ideal | 0.0% | 50.0% | 50.0% |
| [23] | 2018 | USA | TCGA | Adult | NS - TCGA | 2 | 522 | 0.383% | Head and Neck Squamous Cell Carcinoma | 3 | Yes |  | Okay | Ideal | Ideal | 0.0% | 50.0% | 50.0% |
| [61] | 2017 | Commercial Lab - NS | FM | Adult | NS | 0 | 57 | 0.00% | Muco-Epidermoid Carcinoma | 1 | No | Sample Size Ranking | Poor | Ideal | Okay |  |  |  |
| [61] | 2017 | Commercial Lab - NS | FM | Adult | NS | 0 | 20 | 0.00% | Myo-epithelial Carcinoma | 1 | No | Sample Size Ranking | Poor | Ideal | Okay |  |  |  |
| [25] | 2017 | USA | MSK | Mixed | Advanced | 5 | 104 | 4.808% | Salivary Carcinoma (Includes Secretory Carcinomas) | 4 | No | Sample Size Ranking | Poor | Ideal | Okay | 0.0% | 0.0% | 100.0% |
| [18] | 2020 | USA | MSK | Mixed | NS - MSK Advanced | 12 | 227 | 5.286% | Salivary Carcinoma (Includes Secretory) | 4 | No | Sample Size Ranking | Poor | Ideal | Okay | 0.0% | 0.0% | 100.0% |
| [61] | 2017 | Commercial Lab - NS | FM | Adult | NS | 0 | 44 | 0.00% | Salivary Duct Carcinoma | 1 | No | Sample Size Ranking | Poor | Ideal | Okay |  |  |  |
| [61] | 2017 | Commercial Lab - NS | FM | Adult | NS | 0 | 117 | 0.00% | Salivary Gland Adenocarcinoma, NOS | 1 | No | Sample Size Ranking | Poor | Ideal | Okay |  |  |  |
| [26] | 2020 | Commercial Lab - NS | FM | Adult | NS | 24 | 962 | 2.495% | Salivary Gland Cancer | 4 | Yes |  | Okay | Ideal | Okay |  |  |  |
| [27] | 2020 | USA | MSK | Mixed | NS - MSK Advanced | 13 | 256 | 5.078% | Salivary Gland Carcinoma (Includes Secretory) | 4 | No | Sample Size Ranking | Poor | Ideal | Okay | 0.0% | 0.0% | 100.0% |
| [61] | 2017 | Commercial Lab - NS | FM | Adult | NS | 0 | 119 | 0.00% | Salivary Gland Carcinoma, NOS | 1 | No | Sample Size Ranking | Poor | Ideal | Okay |  |  |  |
| [62] | 2020 | International | N/A | Adult | NS | 40 | 48 | 83.33% | Secretory Carcinoma of Salivary Gland | 4 | No | Sample Size Ranking | Poor | Ideal | Ideal | 0.0% | 0.0% | 100.0% |
| [63] | 2020 | USA | N/A | NS | NS | 16 | 19 | 84.2% | Secretory Carcinoma of the Salivary Gland | 4 | No | Method and Sample Size Ranking | Poor | Ideal | Poor |  |  |  |
| [64] | 2020 | USA | MSK | NS | NS | 26 | 29 | 89.66% | Secretory Carcinoma of the Salivary Gland | 4 | No | Method and Sample Size Ranking | Poor | Ideal | Poor |  |  |  |
| [65] | 2021 | Japan | N/A | Mixed | Early | 30 | 33 | 90.91% | Secretory Carcinoma of the Salivary Gland | 4 | No | Method and Sample Size Ranking | Poor | Ideal | Poor | 0.0% | 0.0% | 100.0% |

Supplementary Table S18. NTRK Fusion Prevalence for Lung Cancers

Studies included in meta-analysis are highlighted in blue.

| **Reference** | **YEAR** | **COUNTRY** | **OVERLAP** | **AGE** | **COHORT STAGE** | **No. of positive cases detected** | **No of pts tested** | **% of cases detected** | **TYPE** | **# Per Unique Types** | **Eligible for pooling** | **Why not eligible?** | **Sample Size Ranking** | **Definition BIAS** | **Method Ranking** | **% NTRK1** | **% NTRK2** | **% NTRK3** |
| --- | --- | --- | --- | --- | --- | --- | --- | --- | --- | --- | --- | --- | --- | --- | --- | --- | --- | --- |
| [66] | 2013 | USA | Dana Farber | Adult | Mixed | 3 | 91 | 3.30% | EGFR, ALK, KRAS, ROS WT Lung Adenocarcinoma | 1 | No | Method & Sample Size Ranking | Poor | Not Ideal | Poor | 100.0% | 0.0% | 0.0% |
| [67] | 2020 | China | N/A | Adult | NS | 1 | 373 | 0.27% | BRAF,KRAS, EGFR WT Lung Adenocarcinoma | 1 | No | Sample Size Ranking | Poor | Not Ideal | Okay | 100.0% | 0.0% | 0.0% |
| [68] | 2018 | International | N/A | Adult | Advanced | 1 | 56 | 1.786% | EGFR T790M 2nd Gen TKI Resistant Non-Small Cell Lung Cancer | 1 | No | Method & Sample Size Ranking | Poor | Not Ideal | Poor | 100.0% | 0.0% | 0.0% |
| [69] | 2019 | China | N/A | Adult | Early | 0 | 177 | 0.0% | EGFR, KRAS, HER2, BRAF, MET, ALK, RET, ROS1, FGFR WT Lung Adenocarcinoma | 1 | No | Sample Size Ranking | Poor | Ideal | Ideal |  |  |  |
| [70] | 2020 | USA | MSK | Adult | Advanced | 0 | 62 | 0 | EGFRm Osimertinib Resistant Lung | 1 | No | Sample Size Ranking | Poor | Ideal | Okay |  |  |  |
| [71] | 2019 | China | N/A | Adult | Advanced | 4 | 3873 | 0.10% | EGFRm post TKI Non-Small Cell Lung Cancer | 1 | Yes |  | Ideal | Ideal | Okay | 100.0% | 0.0% | 0.0% |
| [72] | 2019 | Hong Kong | N/A | Adult | Mixed | 0 | 59 | 0 | Large Cell Carcinoma | 1 | No | Sample Size Ranking | Poor | Ideal | Okay |  |  |  |
| [73] | 2018 | International | N/A | Adult | Mixed | 1 | 69 | 1.449% | Large Cell Neuroendocrine Carcinomas | 1 | No | Sample Size Ranking | Poor | Ideal | Ideal | 100.0% | 0.0% | 0.0% |
| [74] | 2015 | Japan | N/A | Adult | Mixed | 0 | 106 | 0% | Lung | 4 | No | Sample Size Ranking | Poor | Not Ideal | Ideal |  |  |  |
| [30] | 2020 | Italy | N/A | Unclear | NS | 1 | 132 | 0.758% | Lung | 4 | No | Method & Sample Size Ranking | Poor | Ideal | Poor |  |  |  |
| [75] | 2020 | China | N/A | Adult | NS | 12 | 21115 | 0.06% | Lung | 4 | Yes |  | Ideal | Ideal | Okay | 100.0% | 0.0% | 0.0% |
| [76] | 2020 | China | N/A | Adult | Advanced | 2 | 173 | 1.16% | Lung | 4 | No | Sample Size Ranking | Poor | Ideal | Okay | 100.0% | 0.0% | 0.0% |
| [77] | 2019 | USA | N/A | Adult | Early | 0 | 112 | 0 | Lung Adenocarcinoma | 16 | No | Sample Size Ranking | Poor | Not Ideal | Ideal |  |  |  |
| [78] | 2020 | Singapore | N/A | Adult | Mixed | 0 | 181 | 0 | Lung Adenocarcinoma | 16 | No | Sample Size Ranking | Poor | Ideal | Ideal |  |  |  |
| [79] | 2020 | China | Burning Rock Biotech | Adult | Mixed | 0 | 215 | 0 | Lung Adenocarcinoma | 16 | No | Sample Size Ranking | Poor | Ideal | Okay |  |  |  |
| [80] | 2018 | USA | N/A | NS | Advanced | 0 | 302 | 0 | Lung Adenocarcinoma | 16 | No | Sample Size Ranking | Poor | Ideal | Ideal |  |  |  |
| [81] | 2019 | South Korea | N/A | Adult | Early | 2 | 350 | 0.571% | Lung Adenocarcinoma | 16 | No | Sample Size Ranking | Poor | Ideal | Okay | 0.0% | 0.0% | 100.0% |
| [82] | 2020 | USA | N/A | Only specified by under / over 70 | Advanced | 0 | 415 | 0.00% | Lung Adenocarcinoma | 16 | Yes |  | Okay | Not Ideal | Okay |  |  |  |
| [22] | 2015 | USA | TCGA | Adult | Early | 1 | 487 | 0.205% | Lung Adenocarcinoma | 16 | No | Larger TCGA Cohort Available | Okay | Ideal | Ideal | 0.0% | 100.0% | 0.0% |
| [83] | 2019 | USA | MSK | Adult | Early | 2 | 492 | 0.41% | Lung Adenocarcinoma | 16 | No | Larger MSK Cohort Available | Okay | Ideal | Okay | 50.0% | 0.0% | 50.0% |
| [20] | 2014 | USA | TCGA | Adult | Mixed | 1 | 513 | 0.195% | Lung Adenocarcinoma | 16 | No | Larger TCGA Cohort Available | Okay | Ideal | Ideal | 0.0% | 100.0% | 0.0% |
| [23] | 2018 | USA | TCGA | Adult | NS - TCGA | 1 | 541 | 0.185% | Lung Adenocarcinoma | 16 | Yes |  | Okay | Ideal | Ideal | 0.0% | 100.0% | 0.0% |
| [84] | 2020 | USA | MSK | Adult | Early | 1 | 604 | 0.17% | Lung Adenocarcinoma | 16 | No | Larger MSK Cohort Available | Okay | Ideal | Okay | 100.0% | 0.0% | 0.0% |
| [85] | 2019 | USA | MSK | Adult | Advanced | 7 | 2522 | 0.3% | Lung Adenocarcinoma | 16 | No | Larger MSK Cohort Available | Ideal | Ideal | Ideal | 57.1% | 28.6% | 14.3% |
| [18] | 2020 | USA | MSK | Mixed | NS - MSK Advanced | 6 | 3658 | 0.164% | Lung Adenocarcinoma | 16 | No | Larger MSK Cohort Available | Ideal | Ideal | Okay | 50.0% | 16.7% | 33.3% |
| [27] | 2020 | USA | MSK | Mixed | NS - MSK Advanced | 9 | 3993 | 0.225% | Lung Adenocarcinoma | 16 | Yes |  | Ideal | Ideal | Okay | 66.7% | 11.1% | 22.2% |
| [86] | 2021 | Germany | N/A | Adult | Advanced | 1 | 4033 | 0.02% | Lung Adenocarcinoma | 16 | Yes |  | Ideal | Not Ideal | Ideal | 100.0% | 0.0% | 0.0% |
| [43] | 2018 | Italy | N/A | NS | Advanced | 0 | 312 | 0.000% | Lung Adenocarcinoma | 16 | No | Sample Size Ranking | Poor | Not Ideal | Poor |  |  |  |
| [77] | 2019 | USA | N/A | Adult | Early | 0 | 98 | 0.000% | Lung Squamous Cell Carcinoma | 6 | No | Sample Size Ranking | Poor | Not Ideal | Ideal |  |  |  |
| [86] | 2021 | Germany | N/A | Adult | Advanced | 0 | 99 | 0.000% | Lung Squamous Cell Carcinoma | 6 | No | Sample Size Ranking | Poor | Not Ideal | Ideal |  |  |  |
| [79] | 2020 | China | Burning Rock Biotech | Adult | Mixed | 0 | 103 | 0.000% | Lung Squamous Cell Carcinoma | 6 | No | Sample Size Ranking | Poor | Ideal | Okay |  |  |  |
| [22] | 2015 | USA | TCGA | Adult | Early | 0 | 220 | 0.000% | Lung Squamous Cell Carcinoma | 6 | No | Sample Size Ranking | Poor | Ideal | Ideal |  |  |  |
| [20] | 2014 | USA | TCGA | Adult | Mixed | 0 | 492 | 0.000% | Lung Squamous Cell Carcinoma | 6 | No | Larger TCGA Cohort Available | Okay | Ideal | Ideal |  |  |  |
| [23] | 2018 | USA | TCGA | Adult | NS - TCGA | 0 | 502 | 0.000% | Lung Squamous Cell Carcinoma | 6 | Yes |  | Okay | Ideal | Ideal |  |  |  |
| [87] | 2015 | International | N/A | Adult | Early | 1 | 72 | 1.39% | Mucinous Adenocarcinoma | 1 | No | Sample Size Ranking | Poor | Ideal | Ideal | 100.0% | 0.0% | 0.0% |
| [88] | 2020 | Japan | LC Scrum | Adult | Advanced | 0 | 89 | 0% | Non-Small Cell Lung Cancer | 15 | No | Sample Size Ranking | Poor | Ideal | Ideal |  |  |  |
| [89] | 2021 | India | N/A | Adult | Advanced | 1 | 145 | 0.69% | Non-Small Cell Lung Cancer | 15 | No | Sample Size Ranking | Poor | Ideal | Ideal |  |  |  |
| [90] | 2014 | Japan | N/A | Adult | NS | 0 | 203 | 0% | Non-Small Cell Lung Cancer | 15 | No | Method & Sample Size Ranking | Poor | Not Ideal | Poor |  |  |  |
| [91] | 2019 | China | N/A | Adult | Mixed | 0 | 410 | 0% | Non-Small Cell Lung Cancer | 15 | No | Method Ranking | Okay | Not Ideal | Poor |  |  |  |
| [92] | 2020 | Australia | N/A | NS | NS | 0 | 522 | 0.000% | Non-Small Cell Lung Cancer | 15 | No | Method Ranking | Okay | Ideal | Poor |  |  |  |
| [93] | 2021 | Sweden | N/A | Adult | Mixed | 0 | 617 | 0.0% | Non-Small Cell Lung Cancer | 15 | No | Method Ranking | Okay | Ideal | Poor |  |  |  |
| [25] | 2017 | USA | MSK | Mixed | Advanced | 1 | 1563 | 0.064% | Non-Small Cell Lung Cancer | 15 | No | Larger MSK Cohort Available | Ideal | Ideal | Okay | 100.0% | 0.0% | 0.0% |
| [94] | 2018 | USA | N/A | NS | Mixed | 4 | 1804 | 0.222% | Non-Small Cell Lung Cancer | 15 | Yes |  | Ideal | Ideal | Ideal | 50.0% | 0.0% | 50.0% |
| [95] | 2020 | China | Cancer Sequencing YS Panel | Adult | Mixed | 4 | 1984 | 0.202% | Non-Small Cell Lung Cancer | 15 | Yes |  | Ideal | Ideal | Okay | 50.0% | 0.0% | 50.0% |
| [67] | 2020 | China | N/A | Adult | Advanced | 1 | 2202 | 0.05% | Non-Small Cell Lung Cancer | 15 | Yes |  | Ideal | Not Ideal | Okay | 100.0% | 0.0% | 0.0% |
| [94] | 2018 | USA | MSK | NS | Mixed | 7 | 3068 | 0.228% | Non-Small Cell Lung Cancer | 15 | Yes |  | Ideal | Ideal | Okay | 57.1% | 14.3% | 28.6% |
| [24] | 2019 | Commercial Lab - NS | Caris Life Sciences | Adult | NS | 4 | 4073 | 0.098% | Non-Small Cell Lung Cancer | 15 | Yes |  | Ideal | Ideal | Ideal | 25.0% | 25.0% | 50.0% |
| [31] | 2021 | China | N/A | Adult | Mixed | 0 | 4742 | 0.000% | Non-Small Cell Lung Cancer | 15 | No | Method Ranking | Ideal | Ideal | Poor |  |  |  |
| [96] | 2021 | China | N/A | Adult | NS | 44 | 7395 | 0.6% | Non-Small Cell Lung Cancer | 15 | Yes |  | Ideal | Ideal | Okay |  |  |  |
| [26] | 2020 | Commercial Lab - NS | FM | Adult | NS | 67 | 39746 | 0.169% | Non-Small Cell Lung Cancer | 15 | Yes |  | Ideal | Ideal | Okay |  |  |  |
| [97] | 2021 | Japan | LC Scrum | Adult | Advanced | 0 | 166 | 0 | Non-Squamous Non Small Cell Lung Cancer | 3 | No | Sample Size Ranking | Poor | Ideal | Ideal |  |  |  |
| [98] | 2020 | USA | Dana Farber | Adult | Mixed | 0 | 909 | 0 | Non-Squamous Non Small Cell Lung Cancer | 3 | Yes |  | Okay | Ideal | Okay |  |  |  |
| [99] | 2019 | China | N/A | Adult | Advanced | 0 | 77 | 0.00% | Non-Squamous Non Small-Cell Lung Cancer | 3 | No | Sample Size Ranking | Poor | Ideal | Okay |  |  |  |
| [92] | 2020 | Australia | N/A | NS | NS | 0 | 154 | 0.000% | Pulmonary Neuroendocrine Tumour | 1 | No | Method & Sample Size Ranking | Poor | Ideal | Poor |  |  |  |
| [25] | 2017 | USA | MSK | Mixed | Advanced | 0 | 88 | 0.000% | Small Cell Lung Cancer | 4 | No | Sample Size Ranking | Poor | Ideal | Okay |  |  |  |
| [92] | 2020 | Australia | N/A | NS | NS | 0 | 105 | 0.000% | Small Cell Lung Cancer | 4 | No | Method & Sample Size Ranking | Poor | Ideal | Poor |  |  |  |
| [31] | 2021 | China | N/A | Adult | Mixed | 0 | 111 | 0.000% | Small Cell Lung Cancer | 4 | No | Method & Sample Size Ranking | Poor | Ideal | Poor |  |  |  |
| [100] | 2019 | China | cancer sequencing YS Panel | Adult | Mixed | 2 | 122 | 1.639% | Small Cell Lung Cancer | 4 | No | Sample Size Ranking | Poor | Ideal | Okay | 0.0% | 0.0% | 100.0% |
| [29] | 2021 | Canada | POG Canada | Adult | Advanced | 0 | 69 | 0.000% | Thoracic Primary | 1 | No | Sample Size Ranking | Poor | Ideal | Ideal |  |  |  |

Supplementary Table S19. NTRK Fusion Prevalence for Melanoma & Skin Cancers

Studies included in meta-analysis are highlighted in blue.

| **Reference** | **YEAR** | **COUNTRY** | **OVERLAP** | **AGE** | **COHORT STAGE** | **No. of positive cases detected** | **No of pts tested** | **% of cases detected** | **TYPE** | **# Per Unique Types** | **Eligible for pooling** | **Why not eligible?** | **Sample Size Ranking** | **Definition BIAS** | **Method Ranking** | **% NTRK1** | **% NTRK2** | **% NTRK3** |
| --- | --- | --- | --- | --- | --- | --- | --- | --- | --- | --- | --- | --- | --- | --- | --- | --- | --- | --- |
| [101] | 2017 | USA | N/A | Adult | Mixed | 0 | 22 | 0 | Acral Lentiginous Melanoma | 3 | No | Non-Explicit Zero | Poor | Ideal | Ideal |  |  |  |
| [102] | 2018 | USA | MSK | Adult * | Advanced | 0 | 54 | 0.000% | Acral Melanoma | 3 | No | Sample Size Ranking | Poor | Ideal | Okay |  |  |  |
| [103] | 2020 | Australia | MIA biobank | NS | Mixed | 0 | 83 | 0 | Acral Melanoma | 3 | No | Non-Explicit Zero | Poor | Ideal | Ideal |  |  |  |
| [102] | 2018 | USA | MSK | Adult * | Advanced | 3 | 395 | 0.76% | Cutaneous Melanoma | 3 | Yes |  | Okay | Ideal | Okay | 66.7% | 33.3% | 0.0% |
| [104] | 2020 | USA | N/A | Adult | NS | 0 | 73 | 0% | Cutaneous Sarcomatoid Malignancies | 1 | No | Sample Size Ranking | Poor | Ideal | Ideal |  |  |  |
| [105] | 2020 | USA | Northwestern University Dept. of Dermatology | Mixed | Early | 16 | 128 | 12.50% | Melanocytic Tumors with Spitz Morphology | 3 | Yes |  | Okay | Ideal | Ideal | 62.5% | 0.0% | 37.5% |
| [51] | 2019 | South Korea | Samsung Medical Center | Mixed | Advanced | 0 | 82 | 0.000% | Melanoma | 6 | No | Sample Size Ranking | Poor | Ideal | Ideal |  |  |  |
| [25] | 2017 | USA | MSK | Mixed | Advanced | 1 | 350 | 0.286% | Melanoma | 6 | No | Larger MSK Cohort Available | Poor | Ideal | Okay | 100.0% | 0.0% | 0.0% |
| [23] | 2018 | USA | TCGA | Adult | NS - TCGA | 1 | 476 | 0.210% | Melanoma | 6 | Yes |  | Okay | Ideal | Ideal | 0.0% | 0.0% | 100.0% |
| [18] | 2020 | USA | MSK | Mixed | NS - MSK Advanced | 5 | 932 | 0.536% | Melanoma | 6 | No | Larger MSK Cohort Available | Okay | Ideal | Okay | 60.0% | 20.0% | 20.0% |
| [27] | 2020 | USA | MSK | Mixed | NS - MSK Advanced | 4 | 1125 | 0.356% | Melanoma | 6 | Yes |  | Okay | Ideal | Okay | 50.0% | 25.0% | 25.0% |
| [26] | 2020 | Commercial Lab - NS | FM | Adult | NS | 9 | 5602 | 0.161% | Melanoma | 6 | Yes |  | Ideal | Ideal | Okay |  |  |  |
| [102] | 2018 | USA | MSK | Adult * | Advanced | 0 | 117 | 0.000% | Melanoma Unknown Primary | 1 | No | Sample Size Ranking | Poor | Ideal | Okay |  |  |  |
| [102] | 2018 | USA | MSK | Adult * | Advanced | 1 | 113 | 0.885% | Mucosal/Paramucosal | 1 | No | Sample Size Ranking | Poor | Ideal | Okay |  |  |  |
| [25] | 2017 | USA | MSK | Mixed | Advanced | 0 | 144 | 0.000% | Non-Melanoma Skin | 1 | No | Sample Size Ranking | Poor | Ideal | Okay |  |  |  |
| [106] | 2018 | USA | Northwestern University Dept. of Dermatology | Mixed | NS | 13 | 23 | 56.522% | Pigmented Spindle Cell Nevus of Reed | 1 | No | Sample Size Ranking | Poor | Ideal | Ideal | 0.0% | 0.0% | 100.0% |
| [30] | 2020 | Italy | N/A | Unclear | NS | 0 | 153 | 0.000% | Skin | 1 | No | Method & Sample Size Ranking | Poor | Ideal | Poor |  |  |  |
| [22] | 2015 | USA | TCGA | Adult | Early | 1 | 78 | 1.282% | Skin Cutaneous Melanoma | 3 | No | Larger TCGA Cohort Available | Poor | Ideal | Ideal | 0.0% | 0.0% | 100.0% |
| [20] | 2014 | USA | TCGA | Adult | Mixed | 1 | 374 | 0.267% | Skin Cutaneous Melanoma | 3 | No | Sample Size Ranking | Poor | Ideal | Ideal | 0.0% | 0.0% | 100.0% |
| [107] | 2020 | USA | N/A | Mixed | NS | 1 | 25 | 4.0% | Spitzoid Melanoma (Melanoma with Annotated Spitz Features) | 1 | No | Sample Size Ranking | Poor | Ideal | Ideal | 100.0% | 0.0% | 0.0% |
| [108] | 2014 | International | N/A | Mixed | Early | 23 | 140 | 16.43% | Spitzoid Neoplasms | 3 | No | Method Ranking | Okay | Not Ideal | Poor | 100.0% | 0.0% | 0.0% |
| [106] | 2018 | USA | Northwestern University Dept. of Dermatology | Mixed | NS | 2 | 67 | 2.99% | Spitzoid Neoplasms (Spitz Nevus, Spitzoid Tumours, Spitzoid Melanoma) | 3 | No | Sample Size Ranking | Poor | Ideal | Ideal | 0.0% | 0.0% | 100.0% |
| [106] | 2018 | USA | Northwestern University Dept. of Dermatology | Mixed | NS | 0 | 31 | 0.000% | Sun Protected Melanomas (Acral, Vulvar, Mucosal) | 1 | No | Sample Size Ranking | Poor | Ideal | Ideal |  |  |  |
| [102] | 2018 | USA | MSK | Adult * | Advanced | 0 | 70 | 0.000% | Uveal Melanoma | 2 | No | Sample Size Ranking | Poor | Ideal | Okay |  |  |  |
| [23] | 2018 | USA | TCGA | Adult | NS - TCGA | 0 | 80 | 0.000% | Uveal Melanoma | 2 | No | Sample Size Ranking | Poor | Ideal | Ideal |  |  |  |

* Based on similar studies in cBioPortal, with fewer than 0.5% of patients under 20 years of age

Supplementary Table S20. NTRK Fusion Prevalence for Paediatric Cancers

Studies included in meta-analysis are highlighted in blue.

| **Reference** | **YEAR** | **COUNTRY** | **OVERLAP** | **AGE** | **COHORT STAGE** | **No. of positive cases detected** | **No of pts tested** | **% of cases detected** | **TYPE** | **Sample Size Ranking** | **Definition BIAS** | **Method Ranking** | **% NTRK1** | **% NTRK2** | **% NTRK3** |
| --- | --- | --- | --- | --- | --- | --- | --- | --- | --- | --- | --- | --- | --- | --- | --- |
| [23] | 2018 | USA | St Jude Pecan/TARGET | Paediatric | NS | 0 | 40 | 0.000% | Adrenocortical Carcinoma | Poor | Ideal | Ideal |  |  |  |
| [109] | 2017 | USA | MSK | Paediatric | NS | 4 | 207 | 1.93% | Atypical Spitz Tumours | Okay | Not Ideal | Poor | 0.0% | 0.0% | 100.0% |
| [110] | 2017 | Commercial Lab - NS | FM | Paediatric | NS | 1 | 63 | 1.587% | Bone Sarcoma | Poor | Ideal | Ideal |  |  |  |
| [26] | 2020 | Commercial Lab - NS | FM | Paediatric | NS | 0 | 251 | 0.000% | Bone Sarcoma | Poor | Ideal | Okay |  |  |  |
| [110] | 2017 | Commercial Lab - NS | FM | Paediatric | NS | 0 | 67 | 0.000% | Brain - Astrocytoma | Poor | Ideal | Ideal |  |  |  |
| [110] | 2017 | Commercial Lab - NS | FM | Paediatric | NS | 0 | 27 | 0.000% | Brain - Ependymoma | Poor | Ideal | Ideal |  |  |  |
| [110] | 2017 | Commercial Lab - NS | FM | Paediatric | NS | 1 | 59 | 1.695% | Brain - Glioblastoma | Poor | Ideal | Ideal |  |  |  |
| [110] | 2017 | Commercial Lab - NS | FM | Paediatric | NS | 0 | 29 | 0.000% | Brain - Glioma | Poor | Ideal | Ideal |  |  |  |
| [26] | 2020 | Commercial Lab - NS | FM | Paediatric | NS | 9 | 800 | 1.125% | Brain - Glioma | Ideal | Ideal | Okay |  |  |  |
| [111] | 2014 | International | St Jude Children's Hospital | Paediatric | Advanced | 8 | 118 | 6.78% | Brain - High Grade Glioma | Okay | Ideal | Ideal | 37.5% | 37.5% | 25.0% |
| [23] | 2018 | USA | St Jude Pecan/TARGET | Paediatric | NS | 7 | 132 | 5.303% | Brain - High Grade Glioma | Okay | Ideal | Ideal | 57.1% | 28.6% | 14.3% |
| [23] | 2018 | USA | St Jude Pecan/TARGET | Paediatric | NS | 3 | 120 | 2.500% | Brain - Low Grade Glioma | Okay | Ideal | Ideal | 0.0% | 66.7% | 33.3% |
| [110] | 2017 | Commercial Lab - NS | FM | Paediatric | NS | 0 | 32 | 0.000% | Brain - Medulloblastoma | Poor | Ideal | Ideal |  |  |  |
| [112] | 2021 | USA | N/A | Paediatric | Mixed | 7 | 338 | 2.071% | Central Nervous System Tumours | Ideal | Ideal | Ideal | 0.0% | 85.7% | 14.3% |
| [113] | 2016 | USA | St Jude Children's Hospital | Paediatric | Early | 2 | 91 | 2.2% | Cerebral Low Grade Neuroepithelial Tumours | Okay | Ideal | Okay | 0.0% | 100.0% | 0.0% |
| [23] | 2018 | USA | St Jude Pecan/TARGET | Paediatric | NS | 0 | 29 | 0.000% | Chroroid Plexus Carcinoma | Poor | Ideal | Ideal |  |  |  |
| [114] | 2018 | International | N/A | Paediatric | NS | 0 | 20 | 0.000% | Clear Cell Carcinoma of the Kidney (BCOR Fusion Negative) | Poor | Not Ideal | Poor |  |  |  |
| [26] | 2020 | Commercial Lab - NS | FM | Paediatric | NS | 1 | 408 | 0.245% | CNS Non Glioma | Okay | Ideal | Okay |  |  |  |
| [115] | 2020 | International | N/A | Paediatric | NS | 5 | 22 | 22.73% | Congenital Mesoblastic Nephroma | Poor | Ideal | Ideal | 0.0% | 0.0% | 100.0% |
| [114] | 2018 | International | N/A | Paediatric | NS | 15 | 80 | 18.75% | Congenital Mesoblastic Nephroma | Okay | Not Ideal | Poor | 13.3% | 0.0% | 86.7% |
| [116] | 2020 | Saudi Arabia | N/A | Paediatric | Early | 6 | 48 | 12.50% | Differentiated Thyroid Cancer | Poor | Ideal | Ideal | 16.7% | 0.0% | 83.3% |
| [117] | 2020 | USA | MD Anderson | Paediatric | Advanced | 18 | 69 | 26.09% | Differentiated Thyroid Cancer | Poor | Not Ideal | Okay | 61.1% | 0.0% | 38.9% |
| [118] | 2019 | Germany | INFORM | Paediatric | Mixed | 1 | 21 | 4.76% | Diffuse Intrinsic Pontine Glioma | Poor | Ideal | Ideal | 100.0% | 0.0% | 0.0% |
| [119] | 2019 | International | N/A | Paediatric | Early | 1 | 37 | 2.70% | Dysembryoplastic Neuroepithelial Tumor, Mixed Neuronal-Glial Tumours, Polymorphous Low-Grade Neuroepithelial Tumor of the Young | Poor | Ideal | Ideal | 0.0% | 100.0% | 0.0% |
| [110] | 2017 | Commercial Lab - NS | FM | Paediatric | NS | 0 | 230 | 0.000% | Embryonal - Neuroblastoma | Poor | Ideal | Ideal |  |  |  |
| [110] | 2017 | Commercial Lab - NS | FM | Paediatric | NS | 0 | 29 | 0.000% | Embryonal - Wilms Tumor | Poor | Ideal | Ideal |  |  |  |
| [25] | 2017 | USA | MSK | Mixed | Advanced | 0 | 76 | 0.000% | Embryonal Tumor | Poor | Ideal | Okay |  |  |  |
| [26] | 2020 | Commercial Lab - NS | FM | Paediatric | NS | 1 | 104 | 0.962% | Endocrine - Neuro Cancer | Poor | Ideal | Okay |  |  |  |
| [23] | 2018 | USA | St Jude Pecan/TARGET | Paediatric | NS | 0 | 92 | 0.000% | Ependymoma | Poor | Ideal | Ideal |  |  |  |
| [26] | 2020 | Commercial Lab - NS | FM | Paediatric | NS | 0 | 154 | 0.000% | Ewings Sarcoma | Poor | Ideal | Okay |  |  |  |
| [23] | 2018 | USA | St Jude Pecan/TARGET | Paediatric | NS | 0 | 123 | 0.000% | Ewing's Sarcoma | Poor | Ideal | Ideal |  |  |  |
| [120] | 2020 | USA | N/A | Paediatric | NS | 0 | 23 | 0.000% | Follicular Variant Papillary Thyroid Cancer | Poor | Not Ideal | Ideal |  |  |  |
| [121] | 2020 | USA | N/A | Paediatric | Mixed | 2 | 61 | 3.279% | Glioma, Glioneuronal, or Ependymoma | Poor | Ideal | Ideal | 0.0% | 0.0% | 100.0% |
| [122] | 2020 | International | Some from St Jude | Paediatric | NS | 24 | 241 | 10.0% | Infant Gliomas | Okay | Ideal | Ideal | 12.5% | 29.2% | 58.3% |
| [114] | 2018 | International | N/A | Paediatric | NS | 19 | 27 | 70.370% | Infantile fibrosarcoma | Poor | Not Ideal | Poor | 0.0% | 0.0% | 100.0% |
| [123] | 2019 | International | N/A | Paediatric | Mixed | 8 | 29 | 27.586% | Infantile High Grade Glioma | Poor | Ideal | Poor |  |  |  |
| [123] | 2019 | International | N/A | Paediatric | Mixed | 0 | 83 | 0.000% | Infantile Low Grade Glioma | Okay | Ideal | Poor |  |  |  |
| [124] | 2020 | Russia | N/A | Paediatric | Mixed | 1 | 29 | 3.4% | Inflammatory Myofibroblastic Tumors | Poor | Ideal | Okay | 0.0% | 0.0% | 100.0% |
| [26] | 2020 | Commercial Lab - NS | FM | Paediatric | NS | 0 | 119 | 0.000% | Kidney | Poor | Ideal | Okay |  |  |  |
| [125] | 2020 | Saudi Arabia | N/A | Paediatric | Early | 3 | 37 | 8.11% | Low Grade Glioma | Poor | Ideal | Ideal | 0.0% | 66.7% | 33.3% |
| [126] | 2020 | Canada | N/A | Paediatric |  | 2 | 477 | 0.42% | Low Grade Glioma | Ideal | Ideal | Okay | 0.0% | 100.0% | 0.0% |
| [23] | 2018 | USA | St Jude Pecan/TARGET | Paediatric | NS | 0 | 714 | 0.000% | Medulloblastoma | Okay | Ideal | Ideal |  |  |  |
| [23] | 2018 | USA | St Jude Pecan/TARGET | Paediatric | NS | 0 | 382 | 0.000% | Neuroblastoma | Okay | Ideal | Ideal |  |  |  |
| [23] | 2018 | USA | St Jude Pecan/TARGET | Paediatric | NS | 0 | 53 | 0.000% | Osteosarcoma | Poor | Ideal | Ideal |  |  |  |
| [127] | 2013 | Ukraine | Chernobyl Tissue Bank | Paediatric | NS | 3 | 26 | 11.5% | Papillary Thyroid Cancer | Poor | Ideal | Okay | 33.3% | 0.0% | 66.7% |
| [127] | 2013 | Ukraine | Chernobyl Tissue Bank | Paediatric | NS | 2 | 27 | 7.407% | Papillary Thyroid Cancer | Poor | Ideal | Okay | 0.0% | 0.0% | 100.0% |
| [128] | 2017 | Brazil | N/A | Paediatric | Mixed | 3 | 35 | 8.571% | Papillary Thyroid Cancer | Poor | Not Ideal | Poor | 0.0% | 0.0% | 100.0% |
| [129] | 2014 | Ukraine | Chernobyl Tissue Bank | Paediatric | NS | 9 | 62 | 14.52% | Papillary Thyroid Cancer | Poor | Not Ideal | Poor | 0.0% | 0.0% | 100.0% |
| [112] | 2021 | USA | N/A | Paediatric | Mixed | 10 | 73 | 13.699% | Papillary Thyroid Cancer | Poor | Ideal | Ideal | 40.0% | 0.0% | 60.0% |
| [130] | 2020 | Czech Republic | N/A | Paediatric | Mixed | 17 | 93 | 18.28% | Papillary Thyroid Cancer | Okay | Ideal | Ideal | 17.6% | 0.0% | 82.4% |
| [131] | 2020 | Japan | N/A | Paediatric (range 9-23, median 18) | Early | 7 | 136 | 5.147% | Papillary Thyroid Cancer | Okay | Not Ideal | Poor | 0.0% | 0.0% | 100.0% |
| [132] | 2016 | USA | N/A | Paediatric | Mixed | 7 | 27 | 25.9% | Papillary Thyroid Carcinoma | Poor | Ideal | Okay | 14.3% | 0.0% | 85.7% |
| [120] | 2020 | USA | N/A | Paediatric | NS | 1 | 27 | 3.704% | Papillary Thyroid Carcinoma (Classic) | Poor | Not Ideal | Ideal | 0.0% | 0.0% | 100.0% |
| [26] | 2020 | Commercial Lab - NS | FM | Paediatric | NS | 0 | 262 | 0.000% | Peripheral Nervous System | Poor | Ideal | Okay |  |  |  |
| [133] | 2013 | Germany | PCAWG | Paediatric (8/96 pts over 18) | Mixed | 3 | 96 | 3.125% | Pilocytic Astrocytoma | Okay | Ideal | Ideal | 0.0% | 100.0% | 0.0% |
| [23] | 2018 | USA | St Jude Pecan/TARGET | Paediatric | NS | 0 | 39 | 0.000% | Retinoblastoma | Poor | Ideal | Ideal |  |  |  |
| [23] | 2018 | USA | St Jude Pecan/TARGET | Paediatric | NS | 0 | 58 | 0.000% | Rhabdomyosarcoma | Poor | Ideal | Ideal |  |  |  |
| [26] | 2020 | Commercial Lab - NS | FM | Paediatric | NS | 0 | 220 | 0.000% | Rhabdomyosarcoma | Poor | Ideal | Okay |  |  |  |
| [110] | 2017 | Commercial Lab - NS | FM | Paediatric | NS | 4 | 261 | 1.533% | Soft Tissue Sarcoma (Including Infantile Fibrosarcoma) | Poor | Ideal | Ideal |  |  |  |
| [26] | 2020 | Commercial Lab - NS | FM | Paediatric | NS | 23 | 489 | 4.703% | Soft Tissue Sarcoma NOS | Okay | Ideal | Okay |  |  |  |
| [134] | 2019 | USA | St Jude Children's Hospital | Paediatric | NS | 3 | 49 | 6.12% | Spitzoid Melanoma/Atypical Spitz Tumour | Poor | Ideal | Ideal | 66.7% | 0.0% | 33.3% |
| [23] | 2018 | USA | St Jude Pecan/TARGET | Paediatric | NS | 0 | 91 | 0.000% | Wilms Tumor | Poor | Ideal | Ideal |  |  |  |
| [114] | 2018 | International | N/A | Paediatric | NS | 0 | 208 | 0.000% | Wilms Tumor | Poor | Not Ideal | Poor |  |  |  |

Supplementary Table S21. NTRK Fusion Prevalence for Sarcomas

Studies included in meta-analysis are highlighted in blue.

| **Reference** | **YEAR** | **COUNTRY** | **OVERLAP** | **AGE** | **COHORT STAGE** | **No. of positive cases detected** | **No of pts tested** | **% of cases detected** | **TYPE** | **# Per Unique Types** | **Eligible for pooling** | **Why not eligible?** | **Sample Size Ranking** | **Definition BIAS** | **Method Ranking** | **% NTRK1** | **% NTRK2** | **% NTRK3** |
| --- | --- | --- | --- | --- | --- | --- | --- | --- | --- | --- | --- | --- | --- | --- | --- | --- | --- | --- |
| [25] | 2017 | USA | MSK | Mixed | Advanced | 0 | 115 | 0.000% | Bone Cancer | 2 | No | Sample Size Ranking | Poor | Ideal | Okay |  |  |  |
| [26] | 2020 | Commercial Lab - NS | FM | Adult | NS | 1 | 616 | 0.162% | Bone Sarcoma | 2 | Yes |  | Okay | Ideal | Okay |  |  |  |
| [135] | 2020 | Commercial Lab - NS | FM | Mixed | Mixed | 0 | 83 | 0 | Desmoplastic Small Round Cell Tumors | 1 | No | Sample Size Ranking | Poor | Ideal | Ideal |  |  |  |
| [31] | 2021 | China | N/A | Adult | Mixed | 1 | 35 | 2.857% | Gastrointestinal Stromal Tumour | 4 | No | Method & Sample Size Ranking | Poor | Ideal | Poor | 100.0% | 0.0% | 0.0% |
| [25] | 2017 | USA | MSK | Mixed | Advanced | 0 | 126 | 0.000% | Gastrointestinal Stromal Tumour | 4 | No | Sample Size Ranking | Poor | Ideal | Okay |  |  |  |
| [136] | 2016 | Commercial Lab - NS | FM | Mixed | Mixed | 1 | 186 | 0.538% | Gastrointestinal Stromal Tumour | 4 | No | Sample Size Ranking | Poor | Ideal | Okay | 0.0% | 0.0% | 100.0% |
| [26] | 2020 | Commercial Lab - NS | FM | Adult | NS | 6 | 1009 | 0.595% | Gastrointestinal Stromal Tumour | 4 | Yes |  | Okay | Ideal | Okay |  |  |  |
| [137] | 2020 | Japan | N/A | Mixed | NS | 2 | 40 | 5.00% | Inflammatory Myofibroblastic Tumours | 2 | No | Method & Sample Size Ranking | Poor | Ideal | Poor | 0.0% | 0.0% | 100.0% |
| [138] | 2015 | International | MSK | Mixed | NS | 0 | 62 | 0 | Inflammatory Myofibroblastic Tumours | 2 | No | Method & Sample Size Ranking | Poor | Not Ideal | Poor |  |  |  |
| [26] | 2020 | Commercial Lab - NS | FM | Adult | NS | 5 | 1865 | 0.268% | Leiomyosarcoma | 1 | Yes |  | Ideal | Ideal | Okay |  |  |  |
| [139] | 2018 | Japan | NCC biobank | Adult | Mixed | 0 | 29 | 0 | Myxofibrosarcoma | 1 | No | Sample Size Ranking | Poor | Ideal | Ideal |  |  |  |
| [140] | 2020 | International | N/A | NS | NS | 3 | 113 | 2.65% | Osteosarcoma | 1 | No | Sample Size Ranking | Poor | Ideal | Ideal | 0.0% | 33.3% | 66.7% |
| [29] | 2021 | Canada | POG Canada | Adult | Advanced | 3 | 42 | 7.143% | Sarcoma | 5 | No | Sample Size Ranking | Poor | Ideal | Ideal | 0.0% | 66.7% | 33.3% |
| [20] | 2014 | USA | TCGA | Adult | Mixed | 1 | 103 | 0.971% | Sarcoma | 5 | No | Sample Size Ranking | Poor | Ideal | Ideal | 100.0% | 0.0% | 0.0% |
| [141] | 2021 | China | N/A | Mixed | NS | 6 | 199 | 3.02% | Sarcoma | 5 | No | Sample Size Ranking | Poor | Ideal | Okay | 33.3% | 0.0% | 66.7% |
| [23] | 2018 | USA | TCGA | Adult | NS - TCGA | 2 | 263 | 0.760% | Sarcoma | 5 | No | Sample Size Ranking | Poor | Ideal | Ideal | 100.0% | 0.0% | 0.0% |
| [27] | 2020 | USA | MSK | Mixed | NS - MSK Advanced | 13 | 1915 | 0.679% | Sarcoma | 5 | Yes |  | Ideal | Ideal | Okay | 69.2% | 0.0% | 30.8% |
| [18] | 2020 | USA | MSK | Mixed | NS - MSK Advanced | 9 | 770 | 1.169% | Sarcoma NOS (Excludes Uterine) | 1 | Yes |  | Okay | Ideal | Okay | 88.9% | 0.0% | 11.1% |
| [142] | 2019 | USA | MSK | Mixed | NS (likely advanced MSK) | 6 | 175 | 3.43% | Soft Tissue Sarcoma | 5 | No | Sample Size Ranking | Poor | Ideal | Ideal | 50.0% | 0.0% | 50.0% |
| [24] | 2019 | Commercial Lab - NS | Caris Life Sciences | Adult | NS | 2 | 478 | 0.418% | Soft Tissue Sarcoma | 5 | Yes |  | Okay | Ideal | Ideal | 50.0% | 0.0% | 50.0% |
| [25] | 2017 | USA | MSK | Mixed | Advanced | 1 | 438 | 0.228% | Soft Tissue Sarcoma (Excludes Uterine, GIST) | 5 | Yes |  | Okay | Ideal | Okay | 100.0% | 0.0% | 0.0% |
| [26] | 2020 | Commercial Lab - NS | FM | Adult | NS | 44 | 4164 | 1.057% | Soft Tissue Sarcoma (Excluding GIST, Uterine, Leiomyosarcoma) | 5 | Yes |  | Ideal | Ideal | Okay |  |  |  |
| [31] | 2021 | China | N/A | Adult | Mixed | 0 | 57 | 0.000% | Soft tissue (Excludes GIST) | 5 | No | Method & Sample Size Ranking | Poor | Ideal | Poor |  |  |  |
| [143] | 2019 | USA | MSK | Mixed | NS | 3 | 33 | 9.09% | Thoracic Inflammatory Myofibroblastic Tumors | 1 | No | Method & Sample Size Ranking | Poor | Ideal | Poor | 0.0% | 0.0% | 100.0% |

Supplementary Table S22. NTRK Fusion Prevalence for Thyroid Cancers

Studies included in meta-analysis are highlighted in blue.

| **Reference** | **YEAR** | **COUNTRY** | **OVERLAP** | **AGE** | **COHORT STAGE** | **No. of positive cases detected** | **No of pts tested** | **% of cases detected** | **TYPE** | **# Per Unique Types** | **Eligible for pooling** | **Why not eligible?** | **Sample Size Ranking** | **Definition BIAS** | **Method Ranking** | **% NTRK1** | **% NTRK2** | **% NTRK3** |
| --- | --- | --- | --- | --- | --- | --- | --- | --- | --- | --- | --- | --- | --- | --- | --- | --- | --- | --- |
| [31] | 2021 | China | N/A | Adult | Mixed | 0 | 104 | 0.000% | Cervical | 4 | No | Method & Sample Size Ranking | Poor | Ideal | Poor |  |  |  |
| [144] | 2019 | China | N/A | Adult | Mixed | 1 | 25 | 4.000% | Anaplastic Thyroid Cancer | 4 | No | Sample Size Ranking | Poor | Not Ideal | Okay | 100.0% | 0.0% | 0.0% |
| [145] | 2019 | South Korea | N/A | Adult | Diff by Cohorts | 0 | 27 | 0.000% | Anaplastic Thyroid Cancer | 4 | No | Sample Size Ranking | Poor | Not Ideal | Okay |  |  |  |
| [146] | 2017 | USA | FM | Mixed | Mixed | 1 | 132 | 0.758% | Anaplastic Thyroid Cancer | 4 | No | Sample Size Ranking | Poor | Ideal | Okay | 100.0% | 0.0% | 0.0% |
| [147] | 2018 | Commercial Lab - NS | FM and MSK | Adult | Mixed | 2 | 196 | 1.020% | Anaplastic Thyroid Cancer | 4 | No | Sample Size Ranking | Poor | Ideal | Okay | 100.0% | 0.0% | 0.0% |
| [148] | 2018 | Brazil | N/A | Adult | Mixed | 0 | 71 | 0.000% | Classic Papillary Thyroid Cancer | 2 | No | Method & Sample Size Ranking | Poor | Not Ideal | Poor |  |  |  |
| [149] | 2017 | South Korea | N/A | Adult | NS | 2 | 575 | 0.35% | Classic Papillary Thyroid Cancer | 2 | No | Method Ranking | Ideal | Not Ideal | Poor | 100.0% | 0.0% | 0.0% |
| [150] | 2019 | The Netherlands | N/A | Adult | Advanced | 3 | 59 | 5.08% | Classic Papillary Thyroid Cancer (Radioactive Iodine Refractory) | 1 | No | Sample Size Ranking | Poor | Ideal | Ideal | 33.3% | 0.0% | 66.7% |
| [145] | 2019 | South Korea | N/A | Adult | Diff by Cohorts | 0 | 28 | 0.000% | Focal Anaplastic/Poorly Differentiated Thyroid Cancer | 1 | No | Sample Size Ranking | Poor | Not Ideal | Okay |  |  |  |
| [147] | 2018 | Commercial Lab - NS | FM and MSK | Adult | Mixed | 0 | 65 | 0.000% | Follicular Thyroid Cancer | 2 | No | Sample Size Ranking | Poor | Ideal | Okay |  |  |  |
| [150] | 2019 | The Netherlands | N/A | Adult | Advanced | 0 | 24 | 0.000% | Follicular Thyroid Carcinoma | 2 | No | Sample Size Ranking | Poor | Ideal | Ideal |  |  |  |
| [148] | 2018 | Brazil | N/A | Adult | Mixed | 6 | 45 | 13.33% | Follicular Variant Papillary Thyroid Cancer | 3 | No | Method & Sample Size Ranking | Poor | Not Ideal | Poor | 0.0% | 0.0% | 100.0% |
| [151] | 2016 | South Korea | N/A | Adult | Early | 4 | 48 | 8.333% | Follicular Variant Papillary Thyroid Cancer | 3 | No | Sample Size Ranking | Poor | Ideal | Ideal | 0.0% | 0.0% | 100.0% |
| [149] | 2017 | South Korea | N/A | Adult | NS | 0 | 105 | 0.000% | Follicular Variant Papillary Thyroid Cancer | 3 | No | Method Ranking | Okay | Not Ideal | Poor |  |  |  |
| [150] | 2019 | The Netherlands | N/A | Adult | Advanced | 1 | 35 | 2.857% | Hurtle Cell Carcinoma | 2 | No | Sample Size Ranking | Poor | Ideal | Ideal | 0.0% | 0.0% | 100.0% |
| [147] | 2018 | Commercial Lab - NS | FM and MSK | Adult | Mixed | 0 | 35 | 0.000% | Hurtle Cell Carcinoma | 2 | No | Sample Size Ranking | Poor | Ideal | Okay |  |  |  |
| [152] | 2021 | USA | ThygeNext | Unclear | Early | 1 | 209 | 0.478% | Malignant Thyroid Nodule | 1 | No | Sample Size Ranking | Poor | Not Ideal | Okay | 0.0% | 0.0% | 100.0% |
| [146] | 2017 | USA | FM | Mixed | Mixed | 0 | 77 | 0.000% | Medullary Thyroid Cancer | 1 | No | Sample Size Ranking | Poor | Ideal | Okay |  |  |  |
| [151] | 2016 | South Korea | N/A | Adult | Early | 0 | 30 | 0.000% | Minimally Invasive Follicular Thyroid Cancer | 2 | No | Sample Size Ranking | Poor | Ideal | Ideal |  |  |  |
| [149] | 2017 | South Korea | N/A | Adult | NS | 0 | 21 | 0.000% | Non-Invasive Follicular Thyroid Neoplasm | 2 | No | Method & Sample Size Ranking | Poor | Not Ideal | Poor |  |  |  |
| [145] | 2019 | South Korea | N/A | Adult | Diff by Cohorts | 0 | 31 | 0.00% | Papillary Thyroid Cancer | 14 | No | Sample Size Ranking | Poor | Not Ideal | Okay |  |  |  |
| [153] | 2020 | China | N/A | Adult | Mixed | 3 | 66 | 4.545% | Papillary Thyroid Cancer | 14 | No | Sample Size Ranking | Poor | Not Ideal | Okay | 100.0% | 0.0% | 0.0% |
| [151] | 2016 | South Korea | N/A | Adult | Early | 3 | 77 | 3.90% | Papillary Thyroid Cancer | 14 | Yes |  | Okay | Ideal | Ideal | 33.3% | 0.0% | 66.7% |
| [154] | 2012 | France | N/A | Mixed | Mixed | 2 | 103 | 1.9% | Papillary Thyroid Cancer | 14 | No | Method Ranking | Okay | Not Ideal | Poor |  |  |  |
| [129] | 2014 | USA | N/A | Adult | NS | 3 | 151 | 1.99% | Papillary Thyroid Cancer | 14 | No | Method Ranking | Okay | Not Ideal | Poor | 0.0% | 0.0% | 100.0% |
| [155] | 2019 | Italy | N/A | Adult | Early | 5 | 208 | 2.404% | Papillary Thyroid Cancer | 14 | No | Method Ranking | Okay | Not Ideal | Poor | 100.0% | 0.0% | 0.0% |
| [156] | 2017 | South Korea | N/A |  | Early | 6 | 240 | 2.50% | Papillary Thyroid Cancer | 14 | No | Method Ranking | Okay | Not Ideal | Poor | 16.7% | 0.0% | 83.3% |
| [146] | 2017 | USA | FM | Mixed | Mixed | 2 | 303 | 0.7% | Papillary Thyroid Cancer | 14 | No | Larger FM Cohort Available | Ideal | Ideal | Okay | 50.0% | 0.0% | 50.0% |
| [157] | 2020 | Japan | N/A | Adult | Mixed | 2 | 307 | 0.65% | Papillary Thyroid Cancer | 14 | No | Method Ranking | Ideal | Not Ideal | Poor | 50.0% | 0.0% | 50.0% |
| [147] | 2018 | Commercial Lab - NS - NS | FM and MSK | Adult | Mixed | 5 | 468 | 1.07% | Papillary Thyroid Cancer | 14 | Yes |  | Ideal | Ideal | Okay |  |  |  |
| [158] | 2014 | USA | TCGA | Adult | Mixed | 10 | 484 | 2.07% | Papillary Thyroid Cancer | 14 | Yes |  | Ideal | Not Ideal | Ideal | 40.0% | 0.0% | 60.0% |
| [159] | 2020 | Taiwan | N/A | Mixed | Early | 12 | 525 | 2.29% | Papillary Thyroid Cancer | 14 | No | Method Ranking | Ideal | Not Ideal | Poor | 16.7% | 0.0% | 83.3% |
| [160] | 2020 | China | N/A | Adult | Early | 2 | 168 | 1.19% | Papillary Thyroid Carcinoma | 14 | Yes |  | Okay | Not Ideal | Okay | 100.0% | 0.0% | 0.0% |
| [161] | 2018 | China | N/A | Mixed | NS | 12 | 355 | 3.38% | Papillary Thyroid Carcinoma | 14 | Yes |  | Ideal | Ideal | Ideal | 25.0% | 0.0% | 75.0% |
| [144] | 2019 | China | N/A | Adult | Mixed | 1 | 41 | 2.44% | Poorly Differentiated Thyroid Cancer | 1 | No | Sample Size Ranking | Poor | Not Ideal | Okay | 100.0% | 0.0% | 0.0% |
| [149] | 2017 | South Korea | N/A | Adult | NS | 1 | 39 | 2.564% | Tall Cell Variant Papillary Thyroid Cancer | 1 | No | Method & Sample Size Ranking | Poor | Not Ideal | Poor | 100.0% | 0.0% | 0.0% |
| [31] | 2021 | China | N/A | Adult | Mixed | 0 | 63 | 0.000% | Thyroid | 10 | No | Method & Sample Size Ranking | Poor | Ideal | Poor |  |  |  |
| [26] | 2020 | Commercial Lab - NS | FM | Adult | NS | 17 | 1595 | 1.066% | Thyroid | 10 | Yes |  | Ideal | Ideal | Okay |  |  |  |
| [162] | 2020 | USA | FM | Adult | Advanced | 0 | 55 | 0 | Thyroid Cancer | 10 | No | Sample Size Ranking | Poor | Ideal | Okay |  |  |  |
| [25] | 2017 | USA | MSK | Mixed | Advanced | 1 | 226 | 0.442% | Thyroid Cancer | 10 | No | Larger MSK Cohort Available | Okay | Ideal | Okay | 0.0% | 0.0% | 100.0% |
| [18] | 2020 | USA | MSK | Mixed | NS - MSK Advanced | 10 | 451 | 2.217% | Thyroid Cancer | 10 | No | Larger MSK Cohort Available | Ideal | Ideal | Okay | 40.0% | 0.0% | 60.0% |
| [22] | 2015 | USA | TCGA | Adult | Early | 14 | 494 | 2.834% | Thyroid Cancer | 10 | No | Larger TCGA Cohort Available | Ideal | Ideal | Ideal | 35.7% | 0.0% | 64.3% |
| [23] | 2018 | USA | TCGA | Adult | NS - TCGA | 12 | 513 | 2.339% | Thyroid Cancer | 10 | Yes |  | Ideal | Ideal | Ideal | 41.7% | 0.0% | 58.3% |
| [24] | 2019 | Commercial Lab - NS | Caris Life Sciences | Adult | NS | 4 | 70 | 5.714% | Thyroid Carcinoma | 10 | No | Sample Size Ranking | Poor | Ideal | Ideal | 0.0% | 0.0% | 100.0% |
| [20] | 2014 | USA | TCGA | Adult | Mixed | 12 | 498 | 2.410% | Thyroid Carcinoma | 10 | No | Larger TCGA Cohort Available | Ideal | Ideal | Ideal | 41.7% | 0.0% | 58.3% |
| [27] | 2020 | USA | MSK | Mixed | NS - MSK Advanced | 13 | 571 | 2.277% | Thyroid Carcinoma | 10 | Yes |  | Ideal | Ideal | Okay | 30.8% | 0.0% | 69.2% |

Supplementary Table S23. NTRK Fusion Prevalence for Upper Gastrointestinal Cancers

Studies included in meta-analysis are highlighted in blue.

| **Reference** | **YEAR** | **COUNTRY** | **OVERLAP** | **AGE** | **COHORT STAGE** | **No. of positive cases detected** | **No of pts tested** | **% of cases detected** | **TYPE** | **# Per Unique Types** | **Eligible for pooling** | **Why not eligible?** | **Sample Size Ranking** | **Definition BIAS** | **Method Ranking** | **% NTRK1** | **% NTRK2** | **% NTRK3** |
| --- | --- | --- | --- | --- | --- | --- | --- | --- | --- | --- | --- | --- | --- | --- | --- | --- | --- | --- |
| [25] | 2017 | USA | MSK | Mixed | Advanced | 0 | 25 | 0.000% | Ampullary Carcinoma | 1 | No | Sample Size Ranking | Poor | Ideal | Okay |  |  |  |
| [31] | 2021 | China | N/A | Adult | Mixed | 0 | 149 | 0.000% | Biliary | 6 | No | Sample Size Ranking | Poor | Ideal | Poor |  |  |  |
| [26] | 2020 | Commercial Lab - NS | FM | Adult | NS | 4 | 2182 | 0.183% | Biliary | 6 | Yes |  | Ideal | Ideal | Okay |  |  |  |
| [25] | 2017 | USA | MSK | Mixed | Advanced | 1 | 242 | 0.413% | Biliary Cancer | 6 | No | Sample Size Ranking | Poor | Ideal | Okay | 100.0% | 0.0% | 0.0% |
| [163] | 2019 | Germany | N/A | Adult | Mixed | 0 | 93 | 0 | Biliary Tract Cancer | 6 | No | Method & Sample Size Ranking | Poor | Ideal | Poor |  |  |  |
| [43] | 2018 | Italy | N/A | NS | Advanced | 0 | 24 | 0.000% | Biliary Tract Carcinoma | 6 | No | Method & Sample Size Ranking | Poor | Not Ideal | Poor |  |  |  |
| [18] | 2020 | USA | MSK | Mixed | NS - MSK Advanced | 2 | 553 | 0.362% | Biliary Tract Carcinoma | 6 | Yes |  | Okay | Ideal | Okay | 100.0% | 0.0% | 0.0% |
| [23] | 2018 | USA | TCGA | Adult | NS - TCGA | 0 | 291 | 0.000% | Cholangiocarcinoma | 3 | No | Sample Size Ranking | Poor | Ideal | Ideal |  |  |  |
| [27] | 2020 | USA | MSK | Mixed | NS - MSK Advanced | 2 | 787 | 0.254% | Cholangiocarcinoma | 3 | Yes |  | Okay | Ideal | Okay | 100.0% | 0.0% | 0.0% |
| [26] | 2020 | Commercial Lab - NS | FM | Adult | NS | 6 | 3905 | 0.154% | Cholangiocarcinoma | 3 | Yes |  | Ideal | Ideal | Okay |  |  |  |
| [23] | 2018 | USA | TCGA | Adult | NS - TCGA | 0 | 185 | 0.000% | Esophageal Carcinoma | 1 | No | Sample Size Ranking | Poor | Ideal | Ideal |  |  |  |
| [164] | 2019 | Germany | N/A | Adult | Mixed | 0 | 438 | 0 | Esophagogastric Adenocarcinoma | 1 | No | Method Ranking | Okay | Ideal | Poor |  |  |  |
| [25] | 2017 | USA | MSK | Mixed | Advanced | 0 | 317 | 0.000% | Esophagogastric Carcinoma | 1 | No | Sample Size Ranking | Poor | Ideal | Okay |  |  |  |
| [23] | 2018 | USA | TCGA | Adult | NS - TCGA | 0 | 414 | 0.000% | Gastric Adenocarcinoma | 2 | Yes |  | Okay | Ideal | Ideal |  |  |  |
| [40] | 2015 | South Korea | Samsung Medical Center | Adult | Advanced | 1 | 66 | 1.515% | Gastric Cancer | 3 | No | Sample Size Ranking | Poor | Not Ideal | Poor |  |  |  |
| [51] | 2019 | South Korea | Samsung Medical Center | Mixed | Advanced | 0 | 211 | 0.000% | Gastric Carcinoma | 3 | No | Sample Size Ranking | Poor | Ideal | Ideal |  |  |  |
| [165] | 2020 | Mongolia | N/A | Adult | Mixed | 0 | 70 | 0.000% | Hepatocellular Carcinoma | 6 | No | Sample Size Ranking | Poor | Ideal | Ideal |  |  |  |
| [25] | 2017 | USA | MSK | Mixed | Advanced | 0 | 105 | 0.000% | Hepatocellular Carcinoma | 6 | No | Sample Size Ranking | Poor | Ideal | Okay |  |  |  |
| [20] | 2014 | USA | TCGA | Adult | Mixed | 0 | 194 | 0.000% | Hepatocellular Carcinoma | 6 | No | Sample Size Ranking | Poor | Ideal | Ideal |  |  |  |
| [166] | 2020 | South Korea | N/A | Adult | Early | 0 | 288 | 0.000% | Hepatocellular Carcinoma | 6 | No | Sample Size Ranking | Poor | Not Ideal | Poor |  |  |  |
| [23] | 2018 | USA | TCGA | Adult | NS - TCGA | 0 | 374 | 0.000% | Hepatocellular Carcinoma | 6 | No | Sample Size Ranking | Poor | Ideal | Ideal |  |  |  |
| [31] | 2021 | China | N/A | Adult | Mixed | 0 | 499 | 0.000% | Hepatocellular Carcinoma | 6 | No | Method Ranking | Okay | Ideal | Poor |  |  |  |
| [107] | 2014 | Commercial Lab - NS | FM | Adult | Mixed | 1 | 28 | 3.57% | Intrahepatic Cholangiocarcinoma | 1 | No | Sample Size Ranking | Poor | Ideal | Okay | 100.0% | 0.0% | 0.0% |
| [26] | 2020 | Commercial Lab - NS | FM | Adult | NS | 2 | 1064 | 0.188% | Liver | 1 | Yes |  | Okay | Ideal | Okay |  |  |  |
| [29] | 2021 | Canada | POG Canada | Adult | Advanced | 0 | 42 | 0.000% | Pancreas | 5 | No | Sample Size Ranking | Poor | Ideal | Ideal |  |  |  |
| [26] | 2020 | Commercial Lab - NS | FM | Adult | NS | 15 | 11989 | 0.125% | Pancreas | 5 | Yes |  | Ideal | Ideal | Okay |  |  |  |
| [31] | 2021 | China | N/A | Adult | Mixed | 0 | 178 | 0.000% | Pancreatic | 5 | No | Method & Sample Size Ranking | Poor | Ideal | Poor |  |  |  |
| [23] | 2018 | USA | TCGA | Adult | NS - TCGA | 1 | 179 | 0.559% | Pancreatic Adenocarcinoma | 2 | No | Sample Size Ranking | Poor | Ideal | Ideal | 0.0% | 0.0% | 100.0% |
| [27] | 2020 | USA | MSK | Mixed | NS - MSK Advanced | 5 | 1492 | 0.335% | Pancreatic Adenocarcinoma | 2 | Yes |  | Ideal | Ideal | Okay | 20.0% | 0.0% | 80.0% |
| [25] | 2017 | USA | MSK | Mixed | Advanced | 2 | 490 | 0.408% | Pancreatic Cancer | 5 | No | Larger MSK Cohort Available | Okay | Ideal | Okay | 0.0% | 0.0% | 100.0% |
| [18] | 2020 | USA | MSK | Mixed | NS - MSK Advanced | 4 | 1315 | 0.304% | Pancreatic Cancer | 5 | Yes |  | Ideal | Ideal | Okay | 25.0% | 0.0% | 75.0% |
| [25] | 2017 | USA | MSK | Mixed | Advanced | 0 | 35 | 0.000% | Small Bowel | 2 | No | Sample Size Ranking | Poor | Ideal | Okay |  |  |  |
| [26] | 2020 | Commercial Lab - NS | FM | Adult | NS | 1 | 1027 | 0.097% | Small Intestine | 2 | Yes |  | Okay | Ideal | Okay |  |  |  |
| [26] | 2020 | Commercial Lab - NS | FM | Adult | NS | 5 | 3558 | 0.141% | Stomach | 3 | Yes |  | Ideal | Ideal | Okay |  |  |  |
| [20] | 2014 | USA | TCGA | Adult | Mixed | 0 | 285 | 0.000% | Stomach Adenocarcinoma | 2 | No | Sample Size Ranking | Poor | Ideal | Ideal |  |  |  |
| [31] | 2021 | China | N/A | Adult | Mixed | 0 | 497 | 0.000% | Upper Gastrointestinal | 1 | No | Method Ranking | Okay | Ideal | Poor |  |  |  |
| [29] | 2021 | Canada | POG Canada | Adult | Advanced | 0 | 41 | 0.000% | Upper GI (Excluding Pancreas) | 1 | No | Sample Size Ranking | Poor | Ideal | Ideal |  |  |  |

Supplementary Table S24. NTRK Fusion Prevalence for Other Cancers

Studies included in meta-analysis are highlighted in blue.

| **Reference** | **YEAR** | **COUNTRY** | **OVERLAP** | **AGE** | **COHORT STAGE** | **No. of positive cases detected** | **No of pts tested** | **% of cases detected** | **TYPE** | **Sample Size Ranking** | **Definition BIAS** | **Method Ranking** | **% NTRK1** | **% NTRK2** | **% NTRK3** |
| --- | --- | --- | --- | --- | --- | --- | --- | --- | --- | --- | --- | --- | --- | --- | --- |
| [25] | 2017 | USA | MSK | Mixed | Advanced | 0 | 26 | 0.000% | Adrenocortical Carcinoma | Poor | Ideal | Okay |  |  |  |
| [23] | 2018 | USA | TCGA | Adult | NS - TCGA | 0 | 79 | 0.000% | Adrenocortical Carcinoma | Poor | Ideal | Ideal |  |  |  |
| [26] | 2020 | Commercial Lab - NS | FM | Adult | NS | 1 | 947 | 0.106% | Endocrine - Neuro Cancer | Okay | Ideal | Okay |  |  |  |
| [30] | 2020 | Italy | N/A | Unclear | NS | 2 | 159 | 1.258% | Gastrointestinal | Poor | Ideal | Poor |  |  |  |
| [25] | 2017 | USA | MSK | Mixed | Advanced | 0 | 53 | 0.000% | Gastrointestinal Neuroendocrine Tumour | Poor | Ideal | Okay |  |  |  |
| [25] | 2017 | USA | MSK | Mixed | Advanced | 0 | 268 | 0.000% | Germ Cell Tumor | Poor | Ideal | Okay |  |  |  |
| [92] | 2020 | Australia | N/A | NS | NS | 1 | 335 | 0.299% | Malignant Pleural Mesothelioma | Poor | Ideal | Poor | 100.0% | 0.0% | 0.0% |
| [23] | 2018 | USA | TCGA | Adult | NS - TCGA | 0 | 87 | 0.000% | Mesothelioma | Poor | Ideal | Ideal |  |  |  |
| [25] | 2017 | USA | MSK | Mixed | Advanced | 0 | 106 | 0.000% | Mesothelioma | Poor | Ideal | Okay |  |  |  |
| [167] | 2018 | Commercial Lab - NS | FM | NS | NS | 7 | 2417 | 0.3% | Neuroendocrine (Multiple Primary Sites) | Ideal | Ideal | Okay | 42.9% | 14.3% | 42.9% |
| [168] | 2020 | China | N/A | Adult | Advanced | 1 | 35 | 2.86% | Neuroendocrine Neoplasms | Poor | Ideal | Okay | 0.0% | 0.0% | 100.0% |
| [27] | 2020 | USA | MSK | Mixed | NS - MSK Advanced | 1 | 322 | 0.311% | Neuroendocrine Tumor | Poor | Ideal | Okay | 0.0% | 0.0% | 100.0% |
| [169] | 2020 | China | N/A | NS | Early | 2 | 50 | 4.000% | Non Pulmonary Sarcomatoid Carcinomas | Poor | Not Ideal | Okay | 100.0% | 0.0% | 0.0% |
| [26] | 2020 | Commercial Lab - NS | FM | Adult | NS | 2 | 695 | 0.288% | Peritoneum | Okay | Ideal | Okay |  |  |  |
| [23] | 2018 | USA | TCGA | Adult | NS - TCGA | 0 | 184 | 0.000% | Pheochromocytoma or Paranganglioma | Poor | Ideal | Ideal |  |  |  |
| [50] | 2021 | Japan | FM | Adult | Advanced | 1 | 53 | 1.887% | Rare Cancers | Poor | Ideal | Okay |  |  |  |
| [31] | 2021 | China | N/A | Adult | Mixed | 0 | 32 | 0.000% | Thymic | Poor | Ideal | Poor |  |  |  |
| [23] | 2018 | USA | TCGA | Adult | NS - TCGA | 0 | 120 | 0.000% | Thymoma | Poor | Ideal | Ideal |  |  |  |

# Comparison with other key studies

Supplementary Table S25. Comparison of estimates between our study and Forsythe et al. and Foundation Medicine.

| **Cancer type** | **Our Study** | **Forsythe et al. (2020)** [10] | **Foundation Medicine** [26, 170] |
| --- | --- | --- | --- |
| NSCLC | 0.19% (0.11%-0.33%) | 0.17% (0.09-0.25%) | 0.17% (0.13%-0.21%) [26] |
| Lung Adenocarcinoma | 0.09% (0.03%-0.31%) |  | 0.26% 95/37015 [170] |
| Breast Carcinoma | 0.21% (0.16%-0.27%) | 0.10% (0.03%-0.18%) | 0.23% (0.17%-0.31%) [26] |
| Melanoma | 0.19% (0.12%-0.33%) | - | 0.16% (0.07%-0.30%) |
| Cutaneous Melanoma | 0.76% (0.16%-2.20%) | 0.31% (0.07%-0.55%) | 0.17% (6/3449) |
| Colorectal | 0.22% (0.18%-0.28%) | - | 0.21% (0.16%-0.28%) |
| Colorectal Adenocarcinoma | 0.20% (0.09%-0.37%) | 0.26% (0.15%-0.36%) | 0.23% (73/28939) |
| Pancreatic | 0.14% (0.09%-0.22%) | - | 0.13% (0.07%-0.21%) |
| Pancreatic Adenocarcinoma | 0.34% (0.08%-0.78%) | 0.31% (0.09%-0.53%) | 0.17% (21/12225) |
| Prostate | 0.14% (0.08%-0.25%) | - | 0.15% (0.08%-0.27%) [26] |
| Prostate Adenocarcinoma | 0.00% (0.00%-0.73%) | - | 0.22% (20/9056) |

Lung adenocarcinoma for our meta-analysis is relatively very low and lower than the pooled NSCLC figure which is counterintuitive as the largest subgroup. This is likely due to a large study with a very low prevalence. Issue is they were only looking for specific NTRK1 fusions, captured as an issue in the assessment but did not include this as criteria as many studies this detail was hard unclear and would have been even fewer studies considered eligible for pooling.

# Bias assessment

Supplementary Table S26. Critical appraisal of included studies.

| **Reference** | **Cancer Site** | **1. Was the target population a nationally representative sample?** | **2. Was the sample frame appropriate to address the target population?** | **3. Were random or exhaustive recruitment undertaken?** | **4. Was the response (result) rate adequate?** | **5. Were the samples tested reflective of the stage of the patient cohort?** | **6. Was an appropriate definition of the condition used?** | **7. Were valid methods used for the identification of NTRK Fusions?** | **8. Was the condition measured in a standard, reliable way for all participants?** | **9. Was the length of the shortest prevalence period for the parameter of interest appropriate?** | **10. Were the numerator and denominator for prevalence appropriate?** | **11. Was the sample size adequate?** | **12. Were the study subjects described in detail?** |
| --- | --- | --- | --- | --- | --- | --- | --- | --- | --- | --- | --- | --- | --- |
| [158] | Thyroid | Not Ideal | Not Ideal | Not Ideal | Not Ideal | Ideal | Not Ideal | Ideal | Ideal | Ideal | Ideal | Good | Ideal |
| [116] | Paediatric | Ideal | Ideal | Ideal | Ideal | Ideal | Ideal | Ideal | Ideal | Ideal | Ideal | Poor | Ideal |
| [140] | Sarcoma | Not Ideal | Not Ideal | Not Ideal | Ideal | Not Ideal | Ideal | Ideal | Not Ideal | Not Ideal | Ideal | Poor | Not Ideal |
| [138] | Sarcoma | Not Ideal | Not Ideal | Not Ideal | Ideal | Ideal | Not Ideal | Not Ideal | Not Ideal | Not Ideal | Not Ideal | Poor | Not Ideal |
| [164] | Upper Gastrointestinal | Not Ideal | Ideal | Ideal | Ideal | Ideal | Ideal | Not Ideal | Ideal | Ideal | Ideal | Okay | Ideal |
| [148] | Thyroid | Not Ideal | Ideal | Ideal | Ideal | Ideal | Not Ideal | Not Ideal | Ideal | Ideal | Ideal | Poor | Not Ideal |
| [63] | Head and Neck | Not Ideal | Not Ideal | Ideal | Ideal | Ideal | Not Ideal | Not Ideal | Not ideal | Ideal | Not Ideal | Poor | Not ideal |
| [85] | Lung | Not Ideal | Not Ideal | Ideal | Not Ideal | Not Ideal | Ideal | Ideal | Not Ideal | Ideal | Ideal | Good | Not Ideal |
| [28] | Breast | Not Ideal | Not Ideal | Ideal | Ideal | Ideal | Ideal | Ideal | Ideal | Not Ideal | Not Ideal | Poor | Not Ideal |
| [146] | Thyroid | Not Ideal | Not Ideal | Ideal | Ideal | Not Ideal | Ideal | Not Ideal | Ideal | Not Ideal | Not Ideal | Good | Not Ideal |
| [165] | Upper Gastrointestinal | Not Ideal | Ideal | Ideal | Not Ideal | Ideal | Ideal | Ideal | Ideal | Ideal | Not Ideal | Poor | Ideal |
| [84] | Lung | Not Ideal | Not Ideal | Not Ideal | Ideal | Ideal | Ideal | Not Ideal | Not Ideal | Ideal | Ideal | Poor | Ideal |
| [59] | Genitourinary | Not Ideal | Ideal | Ideal | Not Ideal | Ideal | Not Ideal | Not Ideal | Not Ideal | Not Ideal | Ideal | Poor | Ideal |
| [72] | Lung | Not Ideal | Ideal | Ideal | Ideal | Not Ideal | Ideal | Not Ideal | Ideal | Ideal | Not Ideal | Poor | Ideal |
| [143] | Sarcoma | Not Ideal | Ideal | Ideal | Ideal | Not Ideal | Ideal | Not Ideal | Not Ideal | Not Ideal | Ideal | Poor | Not Ideal |
| [78] | Lung | Not Ideal | Not Ideal | Not Ideal | Not Ideal | Ideal | Ideal | Ideal | Ideal | Not Ideal | Not Ideal | Poor | Ideal |
| [91] | Lung | Not Ideal | Ideal | Not Ideal | Ideal | Ideal | Not Ideal | Not Ideal | Ideal | Ideal | Ideal | Okay | Ideal |
| [110] | Pan - Paediatric | Not Ideal | Not Ideal | Ideal | Ideal | Not Ideal | Ideal | Not Ideal | Not Ideal | Not Ideal | Ideal | Okay | Not Ideal |
| [166] | Upper Gastrointestinal | Not Ideal | Ideal | Ideal | Ideal | Ideal | Not Ideal | Not Ideal | Ideal | Ideal | Ideal | Poor | Ideal |
| [41] | Colorectal | Not Ideal | Ideal | Ideal | Ideal | Not Ideal | Not Ideal | Not Ideal | Ideal | Not Ideal | Ideal | Poor | Not Ideal |
| [52] | Colorectal | Not Ideal | Ideal | Ideal | Ideal | Ideal | Ideal | Not Ideal | Ideal | Ideal | Ideal | Good | Ideal |
| [135] | Sarcoma | Not Ideal | Not Ideal | Ideal | Ideal | Not Ideal | Ideal | Ideal | Ideal | Not Ideal | Not Ideal | Poor | Ideal |
| [60] | Gynaecological | Not Ideal | Not Ideal | Ideal | Ideal | Ideal | Ideal | Ideal | Ideal | Not Ideal | Ideal | Poor | Ideal |
| [122] | Paediatric | Not Ideal | Not Ideal | Ideal | Ideal | Ideal | Ideal | Ideal | Not Ideal | Not Ideal | Not Ideal | Good | Ideal |
| [47] | Colorectal | Not Ideal | Not Ideal | Ideal | Not Ideal | Ideal | Not Ideal | Not Ideal | Ideal | Ideal | Ideal | Good | Not Ideal |
| [46] | Colorectal | Not Ideal | Not Ideal | Ideal | Ideal | Not Ideal | Ideal | Ideal | Ideal | Ideal | Ideal | Good | Not Ideal |
| [155] | Thyroid | Not Ideal | Ideal | Ideal | Ideal | Ideal | Not Ideal | Not Ideal | Ideal | Ideal | Ideal | Okay | Ideal |
| [128] | Paediatric | Not Ideal | Ideal | Not Ideal | Ideal | Ideal | Not Ideal | Not Ideal | Ideal | Not Ideal | Ideal | Poor | Ideal |
| [101] | Skin/Melanoma | Not Ideal | Not Ideal | Not Ideal | Not Ideal | Not Ideal | Ideal | Ideal | Ideal | Ideal | Ideal | Poor | Ideal |
| [38] | Colorectal Cancer | Not Ideal | Ideal | Not Ideal | Ideal | Not Ideal | Not Ideal | Not Ideal | Ideal | Ideal | Ideal | Okay | Not Ideal |
| [57] | Colorectal | Not Ideal | Ideal | Not Ideal | Ideal | Not Ideal | Ideal | Not Ideal | Ideal | Not Ideal | Ideal | Poor | Ideal |
| [14] | Brain/CNS | Not Ideal | Not Ideal | Ideal | Ideal | Not Ideal | Ideal | Not Ideal | Ideal | Ideal | Ideal | Poor | Ideal |
| [169] | Other | Not Ideal | Ideal | Ideal | Not Ideal | Ideal | Not Ideal | Not Ideal | Not Ideal | Ideal | Ideal | Poor | Ideal |
| [79] | Lung | Ideal | Not Ideal | Ideal | Ideal | Not Ideal | Ideal | Not Ideal | Ideal | Not Ideal | Ideal | Poor | Ideal |
| [144] | Thyroid | Not Ideal | Not Ideal | Ideal | Ideal | Ideal | Not Ideal | Not Ideal | Ideal | Ideal | Ideal | Poor | Ideal |
| [93] | Lung | Ideal | Ideal | Ideal | Not Ideal | Ideal | Ideal | Not Ideal | Ideal | Ideal | Ideal | Good | Ideal |
| [94] | Lung | Not Ideal | Not Ideal | Ideal | Ideal | Not Ideal | Ideal | Ideal | Ideal | Ideal | Ideal | Good | Not Ideal |
| [94] | Lung | Not Ideal | Not Ideal | Ideal | Ideal | Not Ideal | Ideal | Ideal | Ideal | Ideal | Ideal | Good | Not Ideal |
| [13] | Brain/CNS | Not Ideal | Not Ideal | Not ideal | Ideal | Not ideal | Ideal | Ideal | Ideal | Ideal | Ideal | Good | Not Ideal |
| [120] | Paediatric | Not Ideal | Not Ideal | Ideal | Ideal | Not Ideal | Not Ideal | Not Ideal | Ideal | Ideal | Ideal | Poor | Not Ideal |
| [16] | Brain/CNS | Ideal | Not Ideal | Ideal | Ideal | Ideal | Ideal | Ideal | Ideal | Ideal | Ideal | Okay | Not Ideal |
| [97] | Lung | Not Ideal | Ideal | Ideal | Not Ideal | Ideal | Ideal | Ideal | Not Ideal | Ideal | Ideal | Poor | Ideal |
| [24] | Lung | Not Ideal | Not Ideal | Ideal | Ideal | Not Ideal | Ideal | Ideal | Ideal | Ideal | Ideal | Good | Not Ideal |
| [73] | Lung | Not Ideal | Not Ideal | Not Ideal | Not Ideal | Ideal | Ideal | Ideal | Ideal | Not Ideal | Ideal | Poor | Ideal |
| [123] | Paediatric | Not Ideal | Not Ideal | Ideal | Not Ideal | Ideal | Ideal | Not Ideal | Not Ideal | Ideal | Not Ideal | Okay | Ideal |
| [44] | Colorectal | Ideal | Not Ideal | Ideal | Not Ideal | Not Ideal | Ideal | Not Ideal | Ideal | Ideal | Ideal | Okay | Ideal |
| [77] | Lung | Not Ideal | Not Ideal | Ideal | Ideal | Ideal | Not Ideal | Ideal | Ideal | Not Ideal | Ideal | Poor | Ideal |
| [68] | Lung | Not Ideal | Not Ideal | Not Ideal | Not Ideal | Ideal | Not Ideal | Not Ideal | Ideal | Not Ideal | Not Ideal | Poor | Ideal |
| [95] | Lung | Ideal | Not Ideal | Ideal | Ideal | Not Ideal | Ideal | Not Ideal | Ideal | Not Ideal | Not Ideal | Good | Ideal |
| [100] | Lung | Not Ideal | Not Ideal | Ideal | Ideal | Not Ideal | Ideal | Not Ideal | Ideal | Ideal | Ideal | Poor | Ideal |
| [37] | Colorectal | Not Ideal | Not Ideal | Ideal | Ideal | Not Ideal | Ideal | Not Ideal | Ideal | Not Ideal | Not Ideal | Okay | Ideal |
| [58] | Genitourinary | Not Ideal | Not Ideal | Ideal | Ideal | Not Ideal | Ideal | Not Ideal | Not Ideal | Not Ideal | Ideal | Poor | Not Ideal |
| [162] | Thyroid | Not Ideal | Ideal | Ideal | Ideal | Not Ideal | Ideal | Not Ideal | Ideal | Ideal | Ideal | Poor | Ideal |
| [131] | Paediatric | Not Ideal | Ideal | Ideal | Ideal | Ideal | Not Ideal | Not Ideal | Ideal | Ideal | Ideal | Okay | Ideal |
| [133] | Paediatric | Not Ideal | Not Ideal | Not Ideal | Ideal | Ideal | Ideal | Ideal | Ideal | Not Ideal | Ideal | Okay | Ideal |
| [12] | Brain/CNS | Not Ideal | Not Ideal | Ideal | Ideal | Ideal | Ideal | Not Ideal | Not Ideal | Ideal | Not Ideal | Good | Ideal |
| [32] | Breast | Not Ideal | Not Ideal | Not Ideal | Ideal | Ideal | Ideal | Ideal | Ideal | Not Ideal | Ideal | Poor | Ideal |
| [48] | Colorectal | Not Ideal | Ideal | Not Ideal | Not Ideal | Ideal | Ideal | Ideal | Ideal | Ideal | Ideal | Poor | Ideal |
| [50] | Colorectal | Not Ideal | Ideal | Not Ideal | Not Ideal | Not Ideal | Ideal | Not Ideal | Ideal | Ideal | Ideal | Poor | Ideal |
| [51] | Upper Gastrointestinal | Not Ideal | Ideal | Ideal | Not Ideal | Not Ideal | Ideal | Ideal | Ideal | Ideal | Ideal | Poor | Ideal |
| [152] | Thyroid | Not Ideal | Not Ideal | Ideal | Ideal | Ideal | Not Ideal | Not Ideal | Ideal | Ideal | Ideal | Okay | Not Ideal |
| [121] | Paediatric | Not Ideal | Not Ideal | Not Ideal | Ideal | Ideal | Ideal | Ideal | Ideal | Ideal | Not Ideal | Poor | Not Ideal |
| [98] | Lung | Not Ideal | Not Ideal | Ideal | Ideal | Ideal | Ideal | Not Ideal | Not Ideal | Ideal | Not Ideal | Okay | Ideal |
| [153] | Thyroid | Not Ideal | Not Ideal | Not Ideal | Ideal | Not Ideal | Not Ideal | Not Ideal | Ideal | Ideal | Ideal | Poor | Ideal |
| [49] | Colorectal | Not Ideal | Not Ideal | Ideal | Ideal | Not Ideal | Ideal | Not Ideal | Ideal | Not Ideal | Ideal | Good | Not Ideal |
| [92] | Lung | Ideal | Not Ideal | Ideal | Ideal | Not Ideal | Ideal | Not Ideal | Ideal | Not Ideal | Ideal | Okay | Not Ideal |
| [81] | Lung | Not Ideal | Ideal | Not Ideal | Ideal | Ideal | Ideal | Not Ideal | Not Ideal | Not Ideal | Not Ideal | Poor | Not Ideal |
| [156] | Thyroid | Not Ideal | Ideal | Ideal | Ideal | Ideal | Not Ideal | Not Ideal | Ideal | Ideal | Ideal | Okay | Ideal |
| [149] | Thyroid | Not Ideal | Ideal | Ideal | Ideal | Not Ideal | Not Ideal | Not Ideal | Ideal | Ideal | Ideal | Good | Ideal |
| [40] | Colorectal | Not Ideal | Not Ideal | Ideal | Ideal | Not Ideal | Not Ideal | Not Ideal | Ideal | Ideal | Ideal | Poor | Ideal |
| [159] | Thyroid | Not Ideal | Ideal | Ideal | Ideal | Ideal | Not Ideal | Not Ideal | Not Ideal | Ideal | Ideal | Good | Ideal |
| [129] | Paediatric | Not Ideal | Ideal | Ideal | Not Ideal | Ideal | Not Ideal | Not Ideal | Ideal | Ideal | Ideal | Poor | Not Ideal |
| [129] | Thyroid | Not Ideal | Not Ideal | Ideal | Ideal | Ideal | Not Ideal | Not Ideal | Ideal | Ideal | Ideal | Okay | Not Ideal |
| [102] | Skin/Melanoma | Not Ideal | Ideal | Ideal | Ideal | Ideal | Ideal | Not Ideal | Ideal | Ideal | Ideal | Poor | Not Ideal |
| [76] | Lung | Not Ideal | Not Ideal | Ideal | Ideal | Not Ideal | Ideal | Not Ideal | Not Ideal | Ideal | Ideal | Poor | Ideal |
| [160] | Thyroid | Not Ideal | Ideal | Ideal | Ideal | Ideal | Not Ideal | Not Ideal | Ideal | Ideal | Ideal | Okay | Ideal |
| [67] | Lung | Not Ideal | Not Ideal | Ideal | Ideal | Not Ideal | Not Ideal | Not Ideal | Ideal | Ideal | Ideal | Good | Ideal |
| [67] | Lung | Not Ideal | Not Ideal | Ideal | Ideal | Ideal | Not Ideal | Not Ideal | Ideal | Ideal | Ideal | Poor | Ideal |
| [161] | Thyroid | Not Ideal | Ideal | Ideal | Ideal | Ideal | Ideal | Ideal | Ideal | Ideal | Ideal | Good | Ideal |
| [54] | Colorectal | Not Ideal | Not Ideal | Ideal | Ideal | Not Ideal | Ideal | Not Ideal | Ideal | Ideal | Not Ideal | Okay | Not Ideal |
| [168] | Other | Not ideal | Not ideal | Not ideal | Ideal | Not ideal | Ideal | Ideal | Not ideal | Ideal | Ideal | Poor | Ideal |
| [82] | Lung | Not Ideal | Not Ideal | Ideal | Ideal | Not Ideal | Not Ideal | Not Ideal | Not Ideal | Ideal | Ideal | Okay | Ideal |
| [30] | Pan | Not Ideal | Ideal | Ideal | Ideal | Not Ideal | Ideal | Not Ideal | Ideal | Not Ideal | Ideal | Okay | Not Ideal |
| [43] | Colorectal | Not Ideal | Not Ideal | Ideal | Ideal | Not Ideal | Not Ideal | Not Ideal | Ideal | Ideal | Ideal | Okay | Not Ideal |
| [89] | Lung | Not Ideal | Ideal | Ideal | Ideal | Not Ideal | Ideal | Ideal | Ideal | Ideal | Ideal | Poor | Ideal |
| [80] | Lung | Not Ideal | Ideal | Ideal | Not Ideal | Not Ideal | Ideal | Ideal | Ideal | Ideal | Not Ideal | Poor | Not Ideal |
| [104] | Skin/Melanoma | Not Ideal | Ideal | Ideal | Ideal | Ideal | Ideal | Ideal | Ideal | Ideal | Ideal | Poor | Not Ideal |
| [125] | Paediatric | Not Ideal | Ideal | Ideal | Ideal | Ideal | Ideal | Ideal | Ideal | Ideal | Not Ideal | Poor | Ideal |
| [56] | Colorectal | Not Ideal | Not Ideal | Not Ideal | Ideal | Not Ideal | Ideal | Not Ideal | Ideal | Not Ideal | Ideal | Poor | Ideal |
| [103] | Skin/Melanoma | Not Ideal | Ideal | Not Ideal | Ideal | Not Ideal | Ideal | Ideal | Ideal | Not Ideal | Not Ideal | Poor | Ideal |
| [134] | Paediatric | Not Ideal | Ideal | Ideal | Ideal | Ideal | Ideal | Ideal | Ideal | Not Ideal | Ideal | Poor | Ideal |
| [117] | Paediatric | Not Ideal | Ideal | Ideal | Not Ideal | Not Ideal | Not Ideal | Not Ideal | Not Ideal | Ideal | Not Ideal | Poor | Ideal |
| [17] | Brain/CNS | Not Ideal | Ideal | Ideal | Ideal | Ideal | Ideal | Ideal | Not Ideal | Ideal | Ideal | Okay | Ideal |
| [157] | Thyroid | Not Ideal | Ideal | Not Ideal | Ideal | Ideal | Not Ideal | Not Ideal | Ideal | Ideal | Not Ideal | Good | Ideal |
| [139] | Sarcoma | Ideal | Ideal | Not Ideal | Ideal | Not Ideal | Ideal | Ideal | Ideal | Not Ideal | Not Ideal | Poor | Ideal |
| [23] | Pan - Paediatric | Not Ideal | Not Ideal | Ideal | Ideal | Ideal | Ideal | Ideal | Ideal | Ideal | Ideal | Good | Not Ideal |
| [23] | Pan | Not Ideal | Not Ideal | Ideal | Ideal | Ideal | Ideal | Ideal | Ideal | Ideal | Ideal | Good | Not Ideal |
| [69] | Lung | Not Ideal | Ideal | Not Ideal | Not Ideal | Ideal | Ideal | Ideal | Ideal | Ideal | Ideal | Okay | Ideal |
| [53] | Colorectal | Not Ideal | Ideal | Not Ideal | Ideal | Ideal | Ideal | Ideal | Ideal | Ideal | Ideal | Poor | Ideal |
| [171] | Pan - Paediatric | Not Ideal | Not Ideal | Not Ideal | Ideal | Not Ideal | Ideal | Ideal | Ideal | Ideal | Ideal | Good | Not Ideal |
| [130] | Paediatric | Not Ideal | Ideal | Ideal | Ideal | Ideal | Ideal | Ideal | Ideal | Ideal | Ideal | Okay | Ideal |
| [118] | Paediatric | Ideal | Not Ideal | Ideal | Ideal | Ideal | Ideal | Ideal | Not Ideal | Ideal | Ideal | Poor | Ideal |
| [132] | Paediatric | Not Ideal | Ideal | Ideal | Ideal | Ideal | Ideal | Ideal | Not Ideal | Ideal | Ideal | Poor | Ideal |
| [124] | Paediatric | Not Ideal | Ideal | Ideal | Not Ideal | Not Ideal | Ideal | Ideal | Not Ideal | Ideal | Ideal | Poor | Ideal |
| [113] | Paediatric | Not Ideal | Ideal | Ideal | Ideal | Ideal | Ideal | Not Ideal | Not Ideal | Ideal | Not Ideal | Okay | Ideal |
| [105] | Skin/Melanoma | Not Ideal | Ideal | Ideal | Ideal | Ideal | Ideal | Ideal | Ideal | Ideal | Not Ideal | Okay | Not Ideal |
| [107] | Skin/Melanoma | Not Ideal | Ideal | Ideal | Ideal | Ideal | Ideal | Ideal | Ideal | Ideal | Ideal | Poor | Ideal |
| [33] | Breast | Not Ideal | Ideal | Ideal | Ideal | Not Ideal | Ideal | Not Ideal | Ideal | Ideal | Ideal | Poor | Not Ideal |
| [127] | Paediatric | Not Ideal | Ideal | Not Ideal | Ideal | Ideal | Ideal | Ideal | Ideal | Ideal | Ideal | Poor | Not Ideal |
| [18] | Pan | Ideal | Not Ideal | Not Ideal | Not Ideal | Not ideal | Ideal | Ideal | Ideal | Ideal | Ideal | Good | Ideal |
| [34] | Breast | Ideal | Ideal | Ideal | Ideal | Ideal | Ideal | Ideal | Ideal | Ideal | Ideal | Good | Ideal |
| [61] | Head and Neck | Not Ideal | Not Ideal | Ideal | Ideal | Not Ideal | Ideal | Not Ideal | Ideal | Ideal | Ideal | Okay | Not Ideal |
| [107] | Upper Gastrointestinal | Not Ideal | Not Ideal | Ideal | Ideal | Ideal | Ideal | Not Ideal | Ideal | Not Ideal | Ideal | Poor | Ideal |
| [126] | Paediatric | Ideal | Ideal | Not Ideal | Not Ideal | Ideal | Ideal | Not Ideal | Not Ideal | Ideal | Not Ideal | Good | Ideal |
| [154] | Thyroid | Not Ideal | Ideal | Ideal | Ideal | Not Ideal | Not Ideal | Not Ideal | Ideal | Ideal | Ideal | Okay | Ideal |
| [70] | Lung | Not Ideal | Not Ideal | Ideal | Ideal | Ideal | Ideal | Not Ideal | Not Ideal | Not Ideal | Not Ideal | Poor | Ideal |
| [19] | Brain/CNS | Not Ideal | Not Ideal | Not Ideal | Not Ideal | Ideal | Ideal | Ideal | Ideal | Ideal | Not Ideal | Poor | Not Ideal |
| [136] | Sarcoma | Not Ideal | Not Ideal | Ideal | Ideal | Not Ideal | Ideal | Not Ideal | Ideal | Ideal | Ideal | Poor | Not Ideal |
| [87] | Lung | Not Ideal | Not Ideal | Not Ideal | Not Ideal | Ideal | Ideal | Ideal | Not Ideal | Not Ideal | Ideal | Poor | Not Ideal |
| [96] | Lung | Not Ideal | Not Ideal | Ideal | Ideal | Not Ideal | Ideal | Not Ideal | Ideal | Ideal | Ideal | Good | Ideal |
| [167] | Other | Not Ideal | Not Ideal | Ideal | Ideal | Not Ideal | Ideal | Not Ideal | Ideal | Not Ideal | Not Ideal | Good | Not Ideal |
| [62] | Head and Neck | Not Ideal | Ideal | Ideal | Ideal | Not Ideal | Ideal | Ideal | Ideal | Ideal | Ideal | Okay | Ideal |
| [27] | Pan | Not Ideal | Not Ideal | Ideal | Ideal | Not Ideal | Ideal | Not Ideal | Not Ideal | Ideal | Ideal | Good | Not Ideal |
| [20] | Pan | Ideal | Not Ideal | Ideal | Ideal | Not ideal | Ideal | Ideal | Ideal | Ideal | Ideal | Good | Not Ideal |
| [119] | Paediatric | Not Ideal | Not Ideal | Ideal | Not Ideal | Ideal | Ideal | Ideal | Not Ideal | Ideal | Not Ideal | Poor | Ideal |
| [74] | Lung | Not Ideal | Ideal | Not Ideal | Ideal | Not Ideal | Not Ideal | Ideal | Ideal | Ideal | Ideal | Poor | Ideal |
| [90] | Lung | Not Ideal | Not Ideal | Ideal | Ideal | Not Ideal | Not Ideal | Not Ideal | Ideal | Not Ideal | Not Ideal | Poor | Not Ideal |
| [21] | Brain/CNS | Not Ideal | Ideal | Ideal | Ideal | Ideal | Not Ideal | Not Ideal | Ideal | Ideal | Ideal | Okay | Ideal |
| [29] | Pan | Ideal | Not Ideal | Ideal | Ideal | Ideal | Ideal | Ideal | Ideal | Ideal | Not Ideal | Poor | Not Ideal |
| [66] | Lung | Not Ideal | Not Ideal | Not Ideal | Ideal | Not Ideal | Not Ideal | Not Ideal | Not Ideal | Not Ideal | Not Ideal | Poor | Ideal |
| [150] | Thyroid | Ideal | Not Ideal | Ideal | Ideal | Not Ideal | Ideal | Ideal | Ideal | Not Ideal | Ideal | Poor | Ideal |
| [106] | Skin/Melanoma | Not Ideal | Ideal | Ideal | Ideal | Ideal | Ideal | Ideal | Ideal | Ideal | Ideal | Poor | Not Ideal |
| [86] | Lung | Not ideal | Ideal | Ideal | Not ideal | Not ideal | Ideal | Ideal | Ideal | Ideal | Ideal | Good | Not Ideal |
| [35] | Breast | Not Ideal | Not Ideal | Not Ideal | Ideal | Ideal | Ideal | Not Ideal | Ideal | Not Ideal | Not Ideal | Poor | Ideal |
| [55] | Colorectal | Not Ideal | Ideal | Ideal | Ideal | Not Ideal | Ideal | Not Ideal | Ideal | Ideal | Ideal | Okay | Not Ideal |
| [109] | Paediatric | Not Ideal | Ideal | Ideal | Ideal | Ideal | Not Ideal | Not Ideal | Ideal | Ideal | Ideal | Okay | Not Ideal |
| [114] | Paediatric | Not Ideal | Not Ideal | Not Ideal | Ideal | Not Ideal | Not Ideal | Not Ideal | Not Ideal | Not Ideal | Ideal | Okay | Not Ideal |
| [163] | Upper Gastrointestinal | Not Ideal | Ideal | Ideal | Not Ideal | Not Ideal | Ideal | Not Ideal | Ideal | Not Ideal | Ideal | Poor | Ideal |
| [108] | Skin/Melanoma | Not Ideal | Not Ideal | Ideal | Ideal | Ideal | Not Ideal | Not Ideal | Not Ideal | Not Ideal | Ideal | Okay | Ideal |
| [15] | Brain/CNS | Not Ideal | Not Ideal | Ideal | Ideal | Ideal | Ideal | Ideal | Ideal | Ideal | Ideal | Poor | Ideal |
| [111] | Paediatric | Not Ideal | Not Ideal | Not Ideal | Ideal | Ideal | Ideal | Ideal | Not Ideal | Not Ideal | Not Ideal | Poor | Ideal |
| [36] | Breast | Not Ideal | Ideal | Ideal | Ideal | Ideal | Not Ideal | Not Ideal | Ideal | Ideal | Ideal | Poor | Ideal |
| [42] | Colorectal | Not Ideal | Ideal | Not Ideal | Ideal | Ideal | Ideal | Ideal | Ideal | Ideal | Ideal | Poor | Ideal |
| [75] | Lung | Not Ideal | Not Ideal | Ideal | Ideal | Not Ideal | Ideal | Not Ideal | Not ideal | Ideal | Ideal | Good | Not Ideal |
| [99] | Lung | Not Ideal | Not Ideal | Ideal | Not Ideal | Ideal | Ideal | Not Ideal | Ideal | Ideal | Not Ideal | Poor | Ideal |
| [64] | Head and Neck | Not Ideal | Ideal | Ideal | Ideal | Not Ideal | Ideal | Not Ideal | Ideal | Ideal | Not Ideal | Poor | Not Ideal |
| [71] | Lung | Not Ideal | Not Ideal | Ideal | Ideal | Ideal | Ideal | Not Ideal | Not Ideal | Ideal | Not Ideal | Good | Not Ideal |
| [141] | Sarcoma | Not Ideal | Not Ideal | Not Ideal | Ideal | Not Ideal | Ideal | Not Ideal | Ideal | Ideal | Ideal | Poor | Not Ideal |
| [39] | Colorectal | Not Ideal | Ideal | Ideal | Ideal | Not Ideal | Ideal | Not Ideal | Not Ideal | Ideal | Ideal | Okay | Ideal |
| [65] | Salivary Gland | Not Ideal | Ideal | Ideal | Ideal | Ideal | Ideal | Not Ideal | Ideal | Ideal | Ideal | Poor | Ideal |
| [45] | Colorectal Cancer | Not Ideal | Ideal | Not Ideal | Ideal | Not Ideal | Ideal | Not Ideal | Ideal | Ideal | Ideal | Okay | Not Ideal |
| [151] | Thyroid | Not Ideal | Ideal | Not Ideal | Ideal | Ideal | Ideal | Ideal | Ideal | Ideal | Ideal | Okay | Ideal |
| [145] | Thyroid | Not Ideal | Ideal | Not Ideal | Ideal | Not Ideal | Not Ideal | Not ideal | Not Ideal | Not Ideal | Ideal | Poor | Ideal |
| [22] | Pan | Not Ideal | Not Ideal | Not Ideal | Ideal | Ideal | Ideal | Ideal | Ideal | Ideal | Ideal | Good | Not Ideal |
| [26] | Pan | Ideal | Not Ideal | Not Ideal | Ideal | Ideal | Ideal | Ideal | Ideal | Ideal | Ideal | Good | Ideal |
| [26] | Pan - Paediatric | Ideal | Not Ideal | Not Ideal | Ideal | Ideal | Ideal | Ideal | Ideal | Ideal | Ideal | Good | Ideal |
| [25] | Pan | Not Ideal | Not Ideal | Ideal | Not Ideal | Not Ideal | Ideal | Not Ideal | Not Ideal | Ideal | Not Ideal | Good | Not Ideal |
| [31] | Pan | Not Ideal | Not Ideal | Ideal | Not Ideal | Ideal | Ideal | Not Ideal | Ideal | Ideal | Not Ideal | Good | Not Ideal |
| [115] | Paediatric | Not Ideal | Ideal | Ideal | Ideal | Ideal | Ideal | Ideal | Ideal | Ideal | Ideal | Poor | Not Ideal |
| [112] | Pan - Paediatric | Not Ideal | Not Ideal | Ideal | Ideal | Not Ideal | Ideal | Ideal | Ideal | Ideal | Ideal | Okay | Not Ideal |
| [83] | Lung | Not Ideal | Not Ideal | Ideal | Ideal | Ideal | Ideal | Not Ideal | Not Ideal | Ideal | Not Ideal | Okay | Ideal |
| [142] | Sarcoma | Not Ideal | Not Ideal | Ideal | Ideal | Not Ideal | Ideal | Ideal | Ideal | Ideal | Not Ideal | Poor | Not Ideal |
| [147] | Thyroid | Not Ideal | Not Ideal | Not Ideal | Ideal | Not Ideal | Ideal | Not Ideal | Not Ideal | Ideal | Ideal | Good | Not Ideal |
| [88] | Lung | Not Ideal | Ideal | Ideal | Not Ideal | Not Ideal | Ideal | Ideal | Ideal | Ideal | Ideal | Poor | Ideal |

# References

1. Kundra, R., et al., *OncoTree: A Cancer Classification System for Precision Oncology.* JCO Clin Cancer Inform, 2021. **5**: p. 221-230.

2. Higgins JPT, T.J., Chandler J, Cumpston M, Li T, Page MJ, Welch VA *Cochrane Handbook for Systematic Reviews of Interventions; version 6.2*. 2021 [cited 2021 20/03/2021]; Available from: www.training.cochrane.org/handbook.

3. Munn, Z., et al., *Methodological guidance for systematic reviews of observational epidemiological studies reporting prevalence and cumulative incidence data.* Int J Evid Based Healthc, 2015. **13**(3): p. 147-53.

4. Hoy, D., et al., *Assessing risk of bias in prevalence studies: modification of an existing tool and evidence of interrater agreement.* J Clin Epidemiol, 2012. **65**(9): p. 934-9.

5. Migliavaca, C.B., et al., *Quality assessment of prevalence studies: a systematic review.* J Clin Epidemiol, 2020. **127**: p. 59-68.

6. Solomon, J.P., et al., *NTRK fusion detection across multiple assays and 33,997 cases: diagnostic implications and pitfalls.* Mod Pathol, 2020. **33**(1): p. 38-46.

7. Solomon, J.P. and J.F. Hechtman, *Detection of NTRK fusions: Merits and limitations of current diagnostic platforms.* Cancer Research, 2019. **79**(13): p. 3163-3168.

8. Hechtman, J.F., *NTRK insights: best practices for pathologists.* Modern Pathology, 2021.

9. Naing, L., T. Winn, and R. Nordin, *Pratical Issues in Calculating the Sample Size for Prevalence Studies.* Archives of Orofacial Sciences, 2006. **1**.

10. Forsythe, A., et al., *A systematic review and meta-analysis of neurotrophic tyrosine receptor kinase gene fusion frequencies in solid tumors.* Therapeutic Advances in Medical Oncology, 2020. **12**(no pagination).

11. Hsiao, S.J., et al., *Detection of Tumor NTRK Gene Fusions to Identify Patients Who May Benefit from Tyrosine Kinase (TRK) Inhibitor Therapy.* Journal of Molecular Diagnostics, 2019. **21**(4): p. 553-571.

12. Jonsson, P., et al., *Genomic Correlates of Disease Progression and Treatment Response in Prospectively Characterized Gliomas.* Clinical cancer research : an official journal of the American Association for Cancer Research, 2019. **25**(18): p. 5537-5547.

13. Ferguson, S.D., et al., *Targetable gene fusions associate with the IDH wild-type astrocytic lineage in adult gliomas.* Journal of Neuropathology and Experimental Neurology, 2018. **77**(6): p. 437-442.

14. Deng, M.Y., et al., *Molecularly defined diffuse leptomeningeal glioneuronal tumor (DLGNT) comprises two subgroups with distinct clinical and genetic features.* Acta Neuropathologica, 2018. **136**(2): p. 239-253.

15. Woo, H.Y., et al., *Glioblastomas harboring gene fusions detected by next-generation sequencing.* Brain Tumor Pathology, 2020. **37**(4): p. 136-144.

16. Frattini, V., et al., *The integrated landscape of driver genomic alterations in glioblastoma.* Nature genetics, 2013. **45**(10): p. 1141-1149.

17. Noroxe, D.S., et al., *Genomic profiling of newly diagnosed glioblastoma patients and its potential for clinical utility - a prospective, translational study.* Molecular Oncology, 2020. **14**(11): p. 2727-2743.

18. Rosen, E.Y., et al., *Trk fusions are enriched in cancers with uncommon histologies and the absence of canonical driver mutations.* Clinical Cancer Research, 2020. **26**(7): p. 1624-1632.

19. Shah, N., et al., *Exploration of the gene fusion landscape of glioblastoma using transcriptome sequencing and copy number data.* BMC Genomics, 2013. **14**(1).

20. Stransky, N., et al., *The landscape of kinase fusions in cancer.* Nat Commun, 2014. **5**: p. 4846.

21. Trevisan, P., et al., *Clinical and molecular characterization of adult glioblastomas in Southern Brazil.* Journal of Neuropathology and Experimental Neurology, 2019. **78**(4): p. 297-304.

22. Yoshihara, K., et al., *The landscape and therapeutic relevance of cancer-associated transcript fusions.* Oncogene, 2015. **34**(37): p. 4845-54.

23. Okamura, R., et al., *Analysis of NTRK alterations in pan-cancer adult and pediatric malignancies: Implications for NTRK-targeted therapeutics.* JCO Precision Oncology, 2018. **2**: p. 1-20.

24. Gatalica, Z., et al., *Molecular characterization of cancers with NTRK gene fusions.* Modern Pathology, 2019. **32**(1): p. 147-153.

25. Zehir, A., et al., *Mutational landscape of metastatic cancer revealed from prospective clinical sequencing of 10,000 patients.* Nat Med, 2017. **23**(6): p. 703-713.

26. Yoshino, T., et al., *JSCO-ESMO-ASCO-JSMO-TOS: international expert consensus recommendations for tumour-agnostic treatments in patients with solid tumours with microsatellite instability or NTRK fusions.* Annals of Oncology, 2020. **31**(7): p. 861-872.

27. Solomon, J.P., et al., *NTRK fusion detection across multiple assays and 33,997 cases: diagnostic implications and pitfalls.* Modern Pathology, 2020. **33**(1): p. 38-46.

28. Bolkestein, M., et al., *Chromothripsis in Human Breast Cancer.* Cancer research, 2020. **80**(22): p. 4918-4931.

29. Tsang, E.S., et al., *Uncovering clinically relevant gene fusions with integrated genomic and transcriptomic profiling of metastatic cancers.* Clinical Cancer Research, 2021. **27**(2): p. 522-531.

30. Marchetti, A., et al., *An innovative diagnostic strategy for the detection of rare molecular targets to select cancer patients for tumor-agnostic treatments.* Oncotarget, 2020. **10**(68): p. 6957-6968.

31. Zhang, Y., et al., *Pan-cancer circulating tumor DNA detection in over 10,000 Chinese patients.* Nature communications, 2021. **12**(1): p. 11.

32. Kim, J., et al., *Recurrent fusion transcripts detected by whole-transcriptome sequencing of 120 primary breast cancer samples.* Genes Chromosomes and Cancer, 2015. **54**(11): p. 681-691.

33. Remoue, A., et al., *Non-secretory breast carcinomas lack NTRK rearrangements and TRK protein expression.* Pathology International, 2019. **69**(2): p. 94-96.

34. Ross, D.S., et al., *Enrichment of kinase fusions in ESR1 wild-type, metastatic breast cancer revealed by a systematic analysis of 4854 patients.* Annals of Oncology, 2020. **31**(8): p. 991-1000.

35. Vranic, S., et al., *Potential Novel Therapy Targets in Neuroendocrine Carcinomas of the Breast.* Clinical Breast Cancer, 2019. **19**(2): p. 131-136.

36. Wu, N., et al., *Clinicopathologic and molecular characteristics of 44 patients with pure secretory breast carcinoma.* Cancer Biology and Medicine, 2019. **16**(1): p. 139-146.

37. Huang, W., et al., *Characterization of genomic alterations in Chinese colorectal cancer patients.* Japanese journal of clinical oncology, 2021. **51**(1): p. 120-129.

38. Creancier, L., et al., *Chromosomal rearrangements involving the NTRK1 gene in colorectal carcinoma.* Cancer Letters, 2015. **365**(1): p. 107-111.

39. Yaeger, R., et al., *Clinical Sequencing Defines the Genomic Landscape of Metastatic Colorectal Cancer.* Cancer Cell, 2018. **33**(1): p. 125-136.e3.

40. Lee, S.J., et al., *NTRK1 rearrangement in colorectal cancer patients: Evidence for actionable target using patient-derived tumor cell line.* Oncotarget, 2015. **6**(36): p. 39028-39035.

41. Choi, Y., et al., *Cytoplasmic TrkA Expression as a Screen for Detecting NTRK1 Fusions in Colorectal Cancer.* Translational Oncology, 2018. **11**(3): p. 764-770.

42. Wu, S.-M., et al., *Comprehensive transcriptome profiling of Taiwanese colorectal cancer implicates an ethnic basis for pathogenesis.* Scientific reports, 2020. **10**(1): p. 4526.

43. Mauri, G., et al., *TRKA expression and NTRK1 gene copy number across solid tumours.* Journal of Clinical Pathology, 2018. **71**(10): p. 926-931.

44. Guo, Y., et al., *Genomic Alterations of NTRK, POLE, ERBB2, and Microsatellite Instability Status in Chinese Patients with Colorectal Cancer.* Oncologist, 2020. **25**(11): p. e1671-e1680.

45. Yonemaru, J., et al., *NTRK fusion-positive colorectal cancer in Japanese population.* Pathology International, 2021.

46. Cocco, E., et al., *Colorectal Carcinomas Containing Hypermethylated MLH1 Promoter and Wild-Type BRAF/KRAS Are Enriched for Targetable Kinase Fusions.* Cancer research, 2019. **79**(6): p. 1047-1053.

47. Clifton, K., et al., *Identification of actionable fusions as an anti-EGFR resistance mechanism using a circulating tumor DNA assay.* JCO Precision Oncology, 2019. **3**(no pagination).

48. Kloosterman, W.P., et al., *A systematic analysis of oncogenic gene fusions in primary colon cancer.* Cancer Research, 2017. **77**(14): p. 3814-3822.

49. Lasota, J., et al., *Colonic Adenocarcinomas Harboring NTRK Fusion Genes: A Clinicopathologic and Molecular Genetic Study of 16 Cases and Review of the Literature.* The American journal of surgical pathology, 2020. **44**(2): p. 162-173.

50. Kondo, T., et al., *Comprehensive genomic profiling for patients with chemotherapy-naive advanced cancer.* Cancer science, 2021. **112**(1): p. 296-304.

51. Kwon, D., et al., *Cancer Panel Assay for Precision Oncology Clinic: Results from a 1-Year Study.* Translational Oncology, 2019. **12**(11): p. 1488-1495.

52. Chou, A., et al., *NTRK gene rearrangements are highly enriched in MLH1/PMS2 deficient, BRAF wild-type colorectal carcinomas-a study of 4569 cases.* Modern Pathology, 2020. **33**(5): p. 924-932.

53. Park, D.Y., et al., *NTRK1 fusions for the therapeutic intervention of Korean patients with colon cancer.* Oncotarget, 2016. **7**(7): p. 8399-8412.

54. Lieu, C.H., et al., *Comprehensive Genomic Landscapes in Early and Later Onset Colorectal Cancer.* Clinical Cancer Research, 2019. **25**(19): p. 5852-5858.

55. Wang, J., et al., *Prevalence of recurrent oncogenic fusion in mismatch repair-deficient colorectal carcinoma with hypermethylated MLH1 and wild-type BRAF and KRAS.* Modern Pathology, 2019. **32**(7): p. 1053-1064.

56. Morano, F., et al., *Negative hyperselection of patients with RAS and BRAF wild-type metastatic colorectal cancer who received panitumumab-based maintenance therapy.* Journal of Clinical Oncology, 2019. **37**(33): p. 3099-3110.

57. Cremolini, C., et al., *Negative hyper-selection of metastatic colorectal cancer patients for anti-EGFR monoclonal antibodies: The PRESSING case-control study.* Annals of Oncology, 2017. **28**(12): p. 3009-3014.

58. Ikeda, S., et al., *Next-generation sequencing of prostate cancer: genomic and pathway alterations, potential actionability patterns, and relative rate of use of clinical-grade testing.* Cancer biology & therapy, 2019. **20**(2): p. 219-226.

59. Catic, A., et al., *KANK1-NTRK3 fusions define a subset of BRAF mutation negative renal metanephric adenomas.* BMC Medical Genetics, 2020. **21**(1).

60. Cimic, A., et al., *Molecular Profiling Reveals Limited Targetable Biomarkers in Neuroendocrine Carcinoma of the Cervix.* Applied Immunohistochemistry and Molecular Morphology., 2020.

61. Ross, J.S., et al., *Comprehensive genomic profiles of metastatic and relapsed salivary gland carcinomas are associated with tumor type and reveal new routes to targeted therapies.* Ann Oncol, 2017. **28**(10): p. 2539-2546.

62. Skalova, A., et al., *Expanding the Molecular Spectrum of Secretory Carcinoma of Salivary Glands with a Novel VIM-RET Fusion.* American Journal of Surgical Pathology, 2020. **44**(10): p. 1295-1307.

63. Bell, D., et al., *Pan-Trk immunohistochemistry reliably identifies ETV6-NTRK3 fusion in secretory carcinoma of the salivary gland.* Virchows Archiv, 2020. **476**(2): p. 295-305.

64. Xu, B., et al., *Pan-Trk immunohistochemistry is a sensitive and specific ancillary tool for diagnosing secretory carcinoma of the salivary gland and detecting ETV6-NTRK3 fusion.* Histopathology, 2020. **76**(3): p. 375-382.

65. Yamamoto, H., et al., *Pan-tropomyosin receptor kinase immunoreactivity, ETV6-NTRK3 fusion subtypes, and RET rearrangement in salivary secretory carcinoma.* Human Pathology, 2021. **109**: p. 37-44.

66. Vaishnavi, A., et al., *Oncogenic and drug-sensitive NTRK1 rearrangements in lung cancer.* Nature Medicine, 2013. **19**(11): p. 1469-1472.

67. Li, W., et al., *Intergenic Breakpoints Identified by DNA Sequencing Confound Targetable Kinase Fusion Detection in NSCLC.* Journal of thoracic oncology : official publication of the International Association for the Study of Lung Cancer, 2020. **15**(7): p. 1223-1231.

68. Helman, E., et al., *Cell-Free DNA Next-Generation Sequencing Prediction of Response and Resistance to Third-Generation EGFR Inhibitor.* Clinical Lung Cancer, 2018. **19**(6): p. 518-530.e7.

69. Pan, Y., et al., *Detection of Novel NRG1, EGFR, and MET Fusions in Lung Adenocarcinomas in the Chinese Population.* Journal of Thoracic Oncology: Official Publication of the International Association for the Study of Lung Cancer, 2019. **14**(11): p. 2003-2008.

70. Schoenfeld, A.J., et al., *Tumor Analyses Reveal Squamous Transformation and Off-Target Alterations As Early Resistance Mechanisms to First-line Osimertinib in EGFR-Mutant Lung Cancer.* Clinical cancer research : an official journal of the American Association for Cancer Research, 2020. **26**(11): p. 2654-2663.

71. Xu, H., et al., *Characterization of acquired receptor tyrosine-kinase fusions as mechanisms of resistance to EGFR tyrosine-kinase inhibitors.* Cancer Management and Research, 2019. **11**: p. 6343-6351.

72. Chan, A.W., et al., *The Landscape of Actionable Molecular Alterations in Immunomarker-Defined Large-Cell Carcinoma of the Lung.* J Thorac Oncol, 2019. **14**(7): p. 1213-1222.

73. George, J., et al., *Integrative genomic profiling of large-cell neuroendocrine carcinomas reveals distinct subtypes of high-grade neuroendocrine lung tumors.* Nature communications, 2018. **9**(1): p. 1048-1048.

74. Takeda, M., et al., *Clinical application of amplicon-based next-generation sequencing to therapeutic decision making in lung cancer.* Annals of Oncology, 2015. **26**(12): p. 2477-2482.

75. Xia, H., et al., *Evidence of NTRK1 Fusion as Resistance Mechanism to EGFR TKI in EGFR+ NSCLC: Results From a Large-Scale Survey of NTRK1 Fusions in Chinese Patients With Lung Cancer.* Clinical Lung Cancer, 2020. **21**(3): p. 247-254.

76. Li, H., et al., *Analysis of NTRK mutation and clinicopathologic factors in lung cancer patients in northeast China.* International Journal of Biological Markers, 2020. **35**(3): p. 36-40.

77. Haynes, B.C., et al., *An Integrated Next-Generation Sequencing System for Analyzing DNA Mutations, Gene Fusions, and RNA Expression in Lung Cancer.* Transl Oncol, 2019. **12**(6): p. 836-845.

78. Chen, J., et al., *Genomic landscape of lung adenocarcinoma in East Asians.* Nature genetics, 2020. **52**(2): p. 177-186.

79. Ding, Y., et al., *Comparative study on the mutational profile of adenocarcinoma and squamous cell carcinoma predominant histologic subtypes in Chinese non-small cell lung cancer patients.* Thoracic cancer, 2020. **11**(1): p. 103-112.

80. Miller, T.E., et al., *Clinical utility of reflex testing using focused next-generation sequencing for management of patients with advanced lung adenocarcinoma.* J Clin Pathol, 2018. **71**(12): p. 1108-1115.

81. Lee, J.J., et al., *Tracing Oncogene Rearrangements in the Mutational History of Lung Adenocarcinoma.* Cell, 2019. **177**(7): p. 1842-1857.e21.

82. Mambetsariev, I., et al., *Precision medicine and actionable alterations in lung cancer: A single institution experience.* PloS one, 2020. **15**(2): p. e0228188.

83. Zhou, J., et al., *Analysis of Tumor Genomic Pathway Alterations Using Broad-Panel Next-Generation Sequencing in Surgically Resected Lung Adenocarcinoma.* Clinical Cancer Research, 2019. **25**(24): p. 7475-7484.

84. Caso, R., et al., *The Underlying Tumor Genomics of Predominant Histologic Subtypes in Lung Adenocarcinoma.* Journal of thoracic oncology : official publication of the International Association for the Study of Lung Cancer, 2020. **15**(12): p. 1844-1856.

85. Benayed, R., et al., *High yield of RNA sequencing for targetable kinase fusions in lung adenocarcinomas with no mitogenic driver alteration detected by DNA sequencing and low tumor mutation burden.* Clinical Cancer Research, 2019. **25**(15): p. 4712-4722.

86. Volckmar, A.L., et al., *Targeting rare and non-canonical driver variants in NSCLC - An uncharted clinical field.* Lung Cancer, 2021. **154**: p. 131-141.

87. Shim, H.S., et al., *Unique genetic and survival characteristics of invasive mucinous adenocarcinoma of the lung.* Journal of Thoracic Oncology, 2015. **10**(8): p. 1156-1162.

88. Kunimasa, K., et al., *Improvement strategies for successful next-generation sequencing analysis of lung cancer.* Future Oncol, 2020. **16**(22): p. 1597-1606.

89. Mehta, A., et al., *Non-amplification genetic alterations of HER2 gene in non-small cell lung carcinoma.* Journal of Clinical Pathology, 2021. **74**(2): p. 106-110.

90. Tatematsu, T., et al., *Investigation of neurotrophic tyrosine kinase receptor 1 fusions and neurotrophic tyrosine kinase receptor family expression in non-small-cell lung cancer and sensitivity to AZD7451 in vitro.* Molecular and Clinical Oncology, 2014. **2**(5): p. 725-730.

91. Chen, Y., et al., *Lung cancer family history and exposure to occupational/domestic coal combustion contribute to variations in clinicopathologic features and gene fusion patterns in non-small cell lung cancer.* Thoracic Cancer, 2019. **10**(4): p. 695-707.

92. Leal, J.L., et al., *NTRK and ALK rearrangements in malignant pleural mesothelioma, pulmonary neuroendocrine tumours and non-small cell lung cancer.* Lung Cancer, 2020. **146**: p. 154-159.

93. Elfving, H., et al., *Evaluation of NTRK immunohistochemistry as a screening method for NTRK gene fusion detection in non-small cell lung cancer.* Lung Cancer, 2021. **151**: p. 53-59.

94. Farago, A.F., et al., *Clinicopathologic Features of Non-Small-Cell Lung Cancer Harboring an NTRK Gene Fusion.* JCO Precision Oncology, 2018.

95. Hu, J., et al., *Characterization of genomic alterations and the significance of PI3K/mTOR pathway mutations and tumor mutational burden in non-small cell lung cancer.* Oncology reports, 2020. **43**(6): p. 2053-2061.

96. Si, X., et al., *Genomic characteristics of driver genes in Chinese patients with non-small cell lung cancer.* Thoracic Cancer, 2021. **12**(3): p. 357-363.

97. Furuya, N., et al., *Suitability of transbronchial brushing cytology specimens for next-generation sequencing in peripheral lung cancer.* Cancer science, 2021. **112**(1): p. 380-387.

98. Lamberti, G., et al., *Clinicopathological and genomic correlates of programmed cell death ligand 1 (PD-L1) expression in nonsquamous non-small-cell lung cancer.* Annals of oncology : official journal of the European Society for Medical Oncology, 2020. **31**(6): p. 807-814.

99. Xie, F., et al., *Next-Generation Sequencing for Genotyping of Endobronchial Ultrasound-Guided Transbronchial Needle Aspiration Samples in Lung Cancer.* Ann Thorac Surg, 2019. **108**(1): p. 219-226.

100. Hu, J., et al., *Comprehensive genomic profiling of small cell lung cancer in Chinese patients and the implications for therapeutic potential.* Cancer medicine, 2019. **8**(9): p. 4338-4347.

101. Couts, K.L., et al., *Acral lentiginous melanoma harboring a ROS1 gene fusion with clinical response to entrectinib.* JCO Precision Oncology, 2017. **2017**(1): p. 1-7.

102. Lezcano, C., et al., *Primary and Metastatic Melanoma With NTRK Fusions.* The American journal of surgical pathology, 2018. **42**(8): p. 1052-1058.

103. Newell, F., et al., *Whole-genome sequencing of acral melanoma reveals genomic complexity and diversity.* Nature communications, 2020. **11**(1): p. 5259.

104. Miller, T.I., et al., *A genomic survey of sarcomas on sun-exposed skin reveals distinctive candidate drivers and potentially targetable mutations.* Human pathology, 2020. **102**: p. 60-69.

105. Quan, V.L., et al., *Integrating Next-Generation Sequencing with Morphology Improves Prognostic and Biologic Classification of Spitz Neoplasms.* The Journal of investigative dermatology, 2020. **140**(8): p. 1599-1608.

106. VandenBoom, T., et al., *Genomic Fusions in Pigmented Spindle Cell Nevus of Reed.* The American journal of surgical pathology, 2018. **42**(8): p. 1042-1051.

107. Raghavan, S.S., et al., *Spitz melanoma is a distinct subset of spitzoid melanoma.* Modern Pathology, 2020. **33**(6): p. 1122-1134.

108. Wiesner, T., et al., *Kinase fusions are frequent in Spitz tumours and spitzoid melanomas.* Nature communications, 2014. **5**: p. 3116.

109. Wang, L., et al., *Identification of NTRK3 Fusions in Childhood Melanocytic Neoplasms.* The Journal of molecular diagnostics : JMD, 2017. **19**(3): p. 387-396.

110. Chmielecki, J., et al., *Genomic profiling of a large set of diverse pediatric cancers identifies known and novel mutations across tumor spectra.* Cancer Research, 2017. **77**(2): p. 509-519.

111. Wu, G., et al., *The genomic landscape of diffuse intrinsic pontine glioma and pediatric non-brainstem high-grade glioma.* Nature Genetics, 2014. **46**(5): p. 444-450.

112. Zhao, X., et al., *NTRK fusions identified in pediatric tumors: The frequency, fusion partners, and clinical outcome.* JCO Precision Oncology, 2021(5): p. 204-214.

113. Qaddoumi, I., et al., *Genetic alterations in uncommon low-grade neuroepithelial tumors: BRAF, FGFR1, and MYB mutations occur at high frequency and align with morphology.* Acta Neuropathol, 2016. **131**(6): p. 833-45.

114. Wegert, J., et al., *Recurrent intragenic rearrangements of EGFR and BRAF in soft tissue tumors of infants.* Nature Communications, 2018. **9**(1): p. 2378.

115. Zhao, M., et al., *Congenital mesoblastic nephroma is characterised by kinase mutations including EGFR internal tandem duplications, the ETV6-NTRK3 fusion, and the rare KLHL7-BRAF fusion.* Histopathology, 2020. **77**(4): p. 611-621.

116. Alzahrani, A.S., et al., *Genetic alterations in pediatric thyroid cancer using a comprehensive childhood cancer gene panel.* Journal of Clinical Endocrinology and Metabolism, 2020. **105**(10): p. 1-11.

117. Nies, M., et al., *Distant metastases from childhood differentiated thyroid carcinoma: clinical course and mutational landscape.* The Journal of clinical endocrinology and metabolism., 2020. **31**.

118. Pfaff, E., et al., *Brainstem biopsy in pediatric diffuse intrinsic pontine glioma in the era of precision medicine: the INFORM study experience.* European Journal of Cancer, 2019. **114**: p. 27-35.

119. Surrey, L.F., et al., *Genomic Analysis of Dysembryoplastic Neuroepithelial Tumor Spectrum Reveals a Diversity of Molecular Alterations Dysregulating the MAPK and PI3K/mTOR Pathways.* Journal of neuropathology and experimental neurology, 2019. **78**(12): p. 1100-1111.

120. Franco, A.T., et al., *miRNA expression can classify pediatric thyroid lesions and increases the diagnostic yield of mutation testing.* Pediatric Blood and Cancer, 2020. **67**(6).

121. Lake, J.A., et al., *Targeted fusion analysis can aid in the classification and treatment of pediatric glioma, ependymoma, and glioneuronal tumors.* Pediatr Blood Cancer, 2020. **67**(1): p. e28028.

122. Clarke, M., et al., *Infant High-Grade Gliomas Comprise Multiple Subgroups Characterized by Novel Targetable Gene Fusions and Favorable Outcomes.* Cancer discovery, 2020. **10**(7): p. 942-963.

123. Guerreiro Stucklin, A.S., et al., *Alterations in ALK/ROS1/NTRK/MET drive a group of infantile hemispheric gliomas.* Nature Communications, 2019. **10**(1).

124. Preobrazhenskaya, E.V., et al., *Gene rearrangements in consecutive series of pediatric inflammatory myofibroblastic tumors.* Pediatric Blood & Cancer, 2020. **67**(5): p. e28220.

125. Mobark, N.A., et al., *Clinical management and genomic profiling of pediatric low-grade gliomas in Saudi Arabia.* PLoS ONE, 2020. **15**(1).

126. Ryall, S., et al., *Integrated Molecular and Clinical Analysis of 1,000 Pediatric Low-Grade Gliomas.* Cancer Cell, 2020. **37**(4): p. 569-583.e5.

127. Ricarte-Filho, J.C., et al., *Identification of kinase fusion oncogenes in post-Chernobyl radiation-induced thyroid cancers.* Journal of Clinical Investigation, 2013. **123**(11): p. 4935-4944.

128. Cordioli, M.I.C.V., et al., *Fusion Oncogenes Are the Main Genetic Events Found in Sporadic Papillary Thyroid Carcinomas from Children.* Thyroid, 2017. **27**(2): p. 182-188.

129. Leeman-Neill, R.J., et al., *ETV6-NTRK3 is a common chromosomal rearrangement in radiation-associated thyroid cancer.* Cancer, 2014. **120**(6): p. 799-807.

130. Pekova, B., et al., *RET, NTRK, ALK, BRAF, and MET Fusions in a Large Cohort of Pediatric Papillary Thyroid Carcinomas.* Thyroid, 2020. **30**(12): p. 1771-1780.

131. Iwadate, M., et al., *The Clinicopathological Results of Thyroid Cancer With BRAFV600E Mutation in the Young Population of Fukushima.* The Journal of Clinical Endocrinology & Metabolism, 2020. **105**(12): p. e4328-e4336.

132. Prasad, M.L., et al., *NTRK fusion oncogenes in pediatric papillary thyroid carcinoma in northeast United States.* Cancer, 2016. **122**(7): p. 1097-1107.

133. Jones, D.T.W., et al., *Recurrent somatic alterations of FGFR1 and NTRK2 in pilocytic astrocytoma.* Nature Genetics, 2013. **45**(8): p. 927-932.

134. Newman, S., et al., *Clinical genome sequencing uncovers potentially targetable truncations and fusions of MAP3K8 in spitzoid and other melanomas.* Nature Medicine, 2019. **25**(4): p. 597-602.

135. Chow, W.A., et al., *Recurrent secondary genomic alterations in desmoplastic small round cell tumors.* BMC medical genetics, 2020. **21**(1): p. 101.

136. Shi, E., et al., *FGFR1 and NTRK3 actionable alterations in "Wild-Type" gastrointestinal stromal tumors.* Journal of Translational Medicine, 2016. **14**(1).

137. Yamamoto, H., et al., *Diagnostic utility of pan-Trk immunohistochemistry for inflammatory myofibroblastic tumours.* Histopathology, 2020. **76**(5): p. 774-778.

138. Antonescu, C.R., et al., *Molecular characterization of inflammatory myofibroblastic tumors with frequent ALK and ROS1 gene fusions and rare novel RET rearrangement.* The American journal of surgical pathology, 2015. **39**(7): p. 957-967.

139. Ogura, K., et al., *Integrated genetic and epigenetic analysis of myxofibrosarcoma.* Nature communications, 2018. **9**(1): p. 2765.

140. Ameline, B., et al., *NTRK fusions in osteosarcoma are rare and non-functional events.* Journal of Pathology: Clinical Research, 2020. **6**(2): p. 107-112.

141. Xu, L., et al., *Potential application of genomic profiling for the diagnosis and treatment of patients with sarcoma.* Oncology Letters, 2021. **21**(5): p. 353.

142. Zhu, G., et al., *Diagnosis of known sarcoma fusions and novel fusion partners by targeted RNA sequencing with identification of a recurrent ACTB-FOSB fusion in pseudomyogenic hemangioendothelioma.* Mod Pathol, 2019. **32**(5): p. 609-620.

143. Chang, J.C., et al., *Expanding the Molecular Characterization of Thoracic Inflammatory Myofibroblastic Tumors beyond ALK Gene Rearrangements.* Journal of Thoracic Oncology, 2019. **14**(5): p. 825-834.

144. Duan, H., et al., *Mutational profiling of poorly differentiated and anaplastic thyroid carcinoma by the use of targeted next-generation sequencing.* Histopathology, 2019. **75**(6): p. 890-899.

145. Yoo, S.-K., et al., *Integrative analysis of genomic and transcriptomic characteristics associated with progression of aggressive thyroid cancer.* Nature Communications, 2019. **10**(1): p. 2764.

146. Borre, P.V., et al., *Pediatric, adolescent, and young adult thyroid carcinoma harbors frequent and diverse targetable genomic alterations, including kinase fusions.* Oncologist, 2017. **22**(3): p. 255-263.

147. Pozdeyev, N., et al., *Genetic Analysis of 779 Advanced Differentiated and Anaplastic Thyroid Cancers.* Clin Cancer Res, 2018. **24**(13): p. 3059-3068.

148. Bastos, A.U., A.C. de Jesus, and J.M. Cerutti, *ETV6-NTRK3 and STRN-ALK kinase fusions are recurrent events in papillary thyroid cancer of adult population.* European Journal of Endocrinology, 2018. **178**(1): p. 83-91.

149. Lee, S.E., et al., *Molecular profiling of papillary thyroid carcinoma in korea with a high prevalence of BRAF<sup>V600E</sup> mutation.* Thyroid, 2017. **27**(6): p. 802-810.

150. van der Tuin, K., et al., *Targetable gene fusions identified in radioactive iodine refractory advanced thyroid carcinoma.* European Journal of Endocrinology, 2019. **180**(4): p. 235-241.

151. Yoo, S.K., et al., *Comprehensive Analysis of the Transcriptional and Mutational Landscape of Follicular and Papillary Thyroid Cancers.* PLoS Genet, 2016. **12**(8): p. e1006239.

152. Labourier, E. and T.J. Fahey, *Preoperative molecular testing in thyroid nodules with Bethesda VI cytology: Clinical experience and review of the literature.* Diagnostic Cytopathology, 2021. **49**(4): p. E175-E180.

153. Lan, X., et al., *Genomic landscape of metastatic papillary thyroid carcinoma and novel biomarkers for predicting distant metastasis.* Cancer science, 2020. **111**(6): p. 2163-2173.

154. Sassolas, G., et al., *Oncogenic alterations in papillary thyroid cancers of young patients.* Thyroid, 2012. **22**(1): p. 17-26.

155. Colombo, C., et al., *Impact of Mutation Density and Heterogeneity on Papillary Thyroid Cancer Clinical Features and Remission Probability.* Thyroid, 2019. **29**(2): p. 237-251.

156. Lee, M.Y., et al., *Genetic alterations and their clinical implications in high-recurrence risk papillary thyroid cancer.* Cancer Research and Treatment, 2017. **49**(4): p. 906-914.

157. Nozaki, Y., et al., *Clinicopathological features and immunohistochemical utility of NTRK-, ALK-, and ROS1-rearranged papillary thyroid carcinomas and anaplastic thyroid carcinomas.* Human Pathology, 2020. **106**: p. 82-92.

158. Agrawal, N., et al., *Integrated Genomic Characterization of Papillary Thyroid Carcinoma.* Cell, 2014. **159**(3): p. 676-690.

159. Lee, Y.C., et al., *Detection of NTRK1/3 Rearrangements in Papillary Thyroid Carcinoma Using Immunohistochemistry, Fluorescent In Situ Hybridization, and Next-Generation Sequencing.* Endocrine Pathology, 2020. **31**(4): p. 348-358.

160. Li, M., et al., *Genomic characterization of high-recurrence risk papillary thyroid carcinoma in a southern Chinese population.* Diagnostic pathology, 2020. **15**(1): p. 49.

161. Liang, J., et al., *Genetic landscape of papillary thyroid carcinoma in the Chinese population.* Journal of Pathology, 2018. **244**(2): p. 215-226.

162. Iniguez-Ariza, N.M., et al., *Foundation One Genomic Interrogation of Thyroid Cancers in Patients With Metastatic Disease Requiring Systemic Therapy.* The Journal of clinical endocrinology and metabolism, 2020. **105**(7).

163. Westphalen, C.B., et al., *Neurotrophic tropomyosin receptor kinase (NTRK) and nerve growth factor (NGF) are not expressed in Caucasian patients with biliary tract cancers: pooled data from three independent cohorts.* Clinical and Translational Oncology, 2019. **21**(8): p. 1108-1111.

164. Arnold, A., et al., *Analysis of NTRK expression in gastric and esophageal adenocarcinoma (AGE) with pan-TRK immunohistochemistry.* Pathology Research and Practice, 2019. **215**(11).

165. Candia, J., et al., *The genomic landscape of Mongolian hepatocellular carcinoma.* Nature communications, 2020. **11**(1): p. 4383.

166. Choi, S., et al., *Clinical Significance of Trk Receptor Expression as a New Therapeutic Target in Hepatocellular Carcinoma.* Pathology and Oncology Research, 2020. **26**(4): p. 2587-2595.

167. Sigal, D.S., et al., *Comprehensive genomic profiling identifies novel NTRK fusions in neuroendocrine tumors.* Oncotarget, 2018. **9**(88): p. 35809-35812.

168. Lu, M., et al., *Efficacy, Safety, and Biomarkers of Toripalimab in Patients with Recurrent or Metastatic Neuroendocrine Neoplasms: A Multiple-Center Phase Ib Trial.* Clinical cancer research : an official journal of the American Association for Cancer Research, 2020. **26**(10): p. 2337-2345.

169. Ding, Y., et al., *Genetic characterisation of sarcomatoid carcinomas reveals multiple novel actionable mutations and identifies KRAS mutation as a biomarker of poor prognosis.* Journal of Medical Genetics., 2020.

170. Westphalen, C.B., et al., *Genomic context of NTRK1/2/3 fusion-positive tumours from a large real-world population.* NPJ Precis Oncol, 2021. **5**(1): p. 69.

171. Pavlick, D., et al., *Identification of NTRK fusions in pediatric mesenchymal tumors.* Pediatric Blood and Cancer, 2017. **64**(8).
